# 6,6′-Biazulenic core as a platform for unlocking Hammett constants *via* electrochemical free-energy relationships

**DOI:** 10.1039/d6ra00120c

**Published:** 2026-02-11

**Authors:** Joseph A. Mandigo, Shaun R. Kelsey, Jason C. Applegate, Rene C. Sabala, Monisola K. Dairo, Carben R. Weghorn, Raina Fair, Cindy L. Berrie, Ward H. Thompson, Daron E. Janzen, Mikhail V. Barybin

**Affiliations:** a Department of Chemistry, University of Kansas Lawrence KS 66045 USA mbarybin@ku.edu cberrie@ku.edu wthompson@ku.edu; b Department of Chemistry and Biochemistry, St. Catherine University St. Paul MN 55105 USA

## Abstract

Quantitative appreciation of electronic substituent effects is beneficial across the chemical sciences, from reaction kinetics and catalysis to functional materials design and enzymatic processes. While Hammett parameters (*σ* constants) constitute the most widely invoked paradigm, their determination for structurally complex functional groups is often impractical by traditional empirical means. Herein, we demonstrate that the reversible one-step, 2-e^−^ reduction of linearly functionalized 1,1′,3,3′-tetraethoxycarbonyl-6,6′-biazulene provides a simple electrochemical readout of effective Hammett constants. By design, the values obtained align closely with the conventional *σ*_p_ descriptors pertaining to benzenoid systems. This approach not only helps reevaluate previously reported Hammett values but also quantifies the role of intramolecular hydrogen bonding (*e.g.*, C=O⋯H–S) and enables determining effective *σ* constants (*σ*_eff_) for “designer” functional groups, such as –SAuPPh_3_ and –NCCr(CO)_5_. Moreover, the long-range net electron donor/acceptor influence of the substituents –S^−^, –SAuPPh_3_, –SH, –SCH_2_CH_2_CO_2_CH_2_CH_3_, and –NCCr(CO)_5_ on the [(–NC)Cr(CO)_5_] ^13^C NMR reporter across the 6,6′-biazulenic π-linker was unveiled through inverse-linear *δ*(^13^**C**O_trans_) *vs. δ*(^13^**C**N) and *δ*(^13^**C**O_cis_) *vs. δ*(^13^**C**N) correlations. The π-communication along the molecular axis of the 6,6′-biazulenic scaffold was further confirmed *via* Reflection-Absorption Infrared (RAIR) spectroscopic analysis of [(OC)_5_Cr(η^1^-2-isocyano-2′-mercapto-1,1′,3,3′-tetraethoxycarbonyl-6,6′-biazulene)] self-assembled on the Au(111) surface. By uniting redox tunability with rigorous linear free-energy correlations, this work offers both a versatile molecular platform and a straightforward electrochemical strategy for expanding and refining the Hammett parameter domain.

## Abstract 2

Functionalization along the molecular axis of 6,6′-biazulene enables electrochemical Hammett analysis, precise redox tunability, and probing of long-range π-communication.

## Graphical abstract

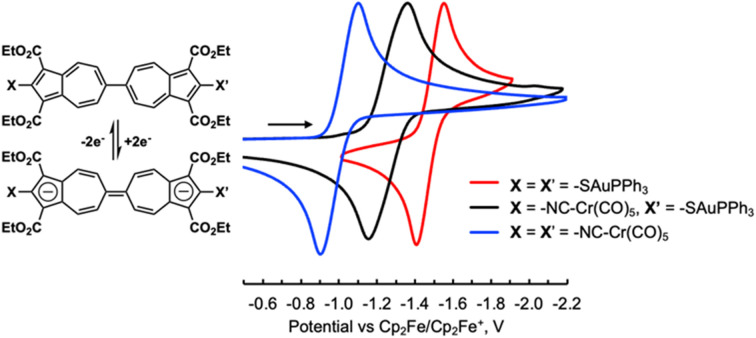

## Introduction

Hammett parameters have long provided a quantitative language for describing how functional groups modulate the electronic landscape of molecular scaffolds across diverse areas of chemistry, from reaction kinetics^1–3^ and catalysis^4–6^ to enzymatic processes^7–9^ and design of functional materials^6,10–12^ and pharmaceuticals.^13,14^ Also referred to as *σ* constants, these parameters were originally conceived to quantify the influence of *para*- or *meta*-ring substituents on the ionization equilibria of benzoic acids and remain the most widely applied metric of substituent electronic effects in aromatic compounds.^1–14^ The recent resurgence of experimental^1,6,15–17^ and computational efforts^18–25^ aimed at broadening and reevaluating this intuitively appealing chemical parameter space underscores the need for robust methodologies capable of addressing “designer” substituents, for which *σ* descriptors are currently unavailable. There are also some persistent ambiguities in the Hammett constant values for certain substituents.^1,26^ For example, there is little consensus on the *σ*_p_ constant for the –NMe_2_ group, with various authors suggesting values in the range of −0.21 to −1.05.^27^ The most frequently cited value of −0.83 was derived by McDaniel^28^ in 1957 from the classical ionization data for *para*-dimethylaminobenzoic acid reported by Johnston^29^ in 1906 (31 years before introduction of the Hammett equation).

**Fig. 1:**
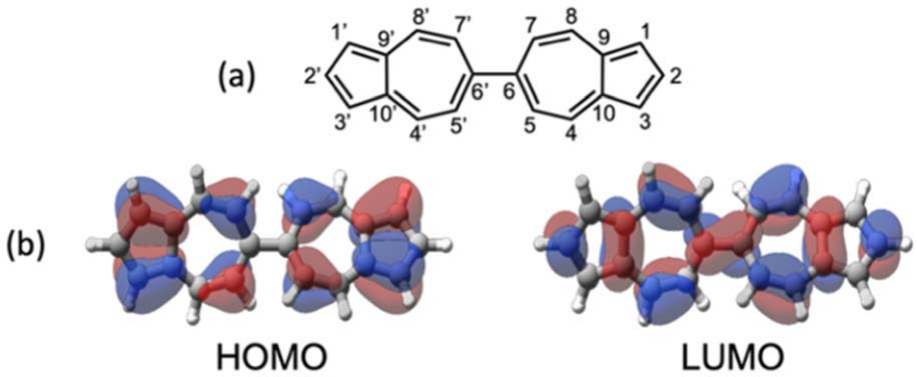
(a) 6,6′-Biazulene and its atom numbering scheme. (b) Highest occupied and lowest unoccupied molecular orbitals of 6,6′-biazulene.

To address the above challenges, our group has turned to 6,6′-biazulene—a π-extended, nonbenzenoid aromatic framework featuring two linearly connected azulenyl units—as a molecular platform for quantifying substituent effects. 6,6′-Biazulene (Fig. 1a) exhibits complementary orbital density distributions in its frontier molecular orbitals: the Highest Occupied Molecular Orbital (HOMO) is distributed over the odd-numbered carbon atoms, whereas the Lowest Unoccupied Molecular Orbital (LUMO) has a π* topology with non-zero orbital density at the even-numbered positions (Fig. 1b). Consequently, functionalization at the 2,2′-carbon atoms of the 6,6′-biazulenic scaffold selectively perturbs the energy of the LUMO whilst leaving the HOMO largely unaffected, therefore enabling tunability of the platform's reduction potential.

**Fig. 2:**
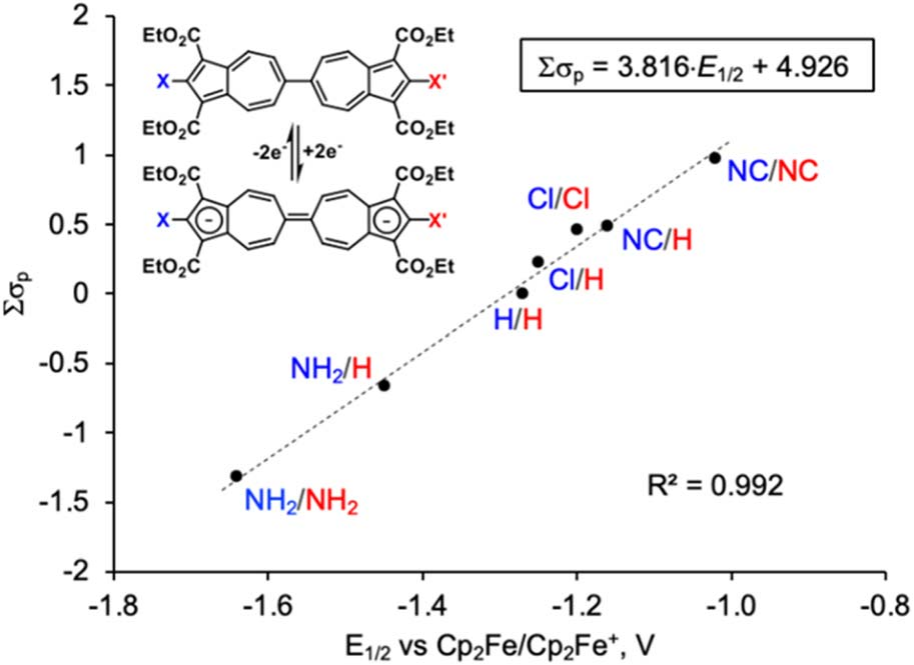
A plot of the combined *σ*_p_ Hammett parameters of the substituents *X* and *X*′ *vs.* the half-wave potential (*E*_1/2_) for the 2-e^−^ reduction of 2,2′-functionalized 6,6′-biazulenes.

Of special note, the one-step, two-electron reduction of the 6,6′-biazulenic core^30–35^ proceeds under a rare regime of reduction potential inversion.^36–38^ Such distinctive behaviour not only simplifies the electrochemical readout by yielding a single, well-defined redox couple but also enhances the sensitivity of the system to even subtle substituent-induced perturbations in electronic structure. To the best of our knowledge, the 6,6′-biazulenic scaffold is the only one among currently accessible positional biazulene isomers^39^ that supports a one-step, two-electron reduction profile. Fig. 2 shows that the sum of literature *σ*_p_ values for our original series^40^ of seven symmetrically and unsymmetrically substituted derivatives of 1,1′,3,3′-tetraethoxycarbonyl-6,6′-biazulene varies linearly with the half-wave potentials (*E*_1/2_), providing a reliable calibration plot that anchors the study reported herein. The intrinsic boundaries of the relationship are determined by the CH_2_Cl_2_/^*n*^Bu_4_NPF_6_ electrolyte solution window used in our electrochemical studies (*ca*. −2 to +2 V *vs.* SCE with *E*_1/2_(Cp_2_Fe^0^/Cp_2_Fe^+^) = 0.46 V).^41,42^

Notably, the *σ*_p_ values used in this plot were determined from the experiments conducted in aqueous media, such that the *σ*_p_ constant of −0.66 (ref. 26) for the –NH_2_ substituent inherently includes the contribution from solvation-induced hydrogen bonding. On the other hand, 2,2′-diamino-1,1′,3,3′-tetraethoxy-carbonyl-6,6′-biazulene engages in intramolecular H-bonding interactions between its amino and carboxylic ester groups, as evidenced by a markedly deshielded ^1^H NMR resonance at *δ*(NH_2_) = 7.88 ppm^34^ compared to 4.58 ppm for 2-aminoazulene in CDCl_3_ (Fig. S29). Thus, the H-bonding in this molecule may be regarded as a structural surrogate for solvent-mediated stabilization affecting the –NH_2_ functional group in aqueous environments.

Building on this recently established linear free-energy relationship, we set out to implement the reverse Hammett analysis by considering electrochemical signatures of the 1,1′,3,3′-tetraethoxycarbonyl-6,6′-biazulenic core to extract effective *σ* values (*σ*_eff_) for electronically and structurally complex functional groups such as –SAuPPh_3_ and –NCCr(CO)_5_ that should map directly onto the conventional *σ*_p_ scale.

## Results and discussion

### Synthesis and characterization of symmetrically and asymmetrically 2,2′-functionalized 6,6′-biazulenes

**Scheme 1:**
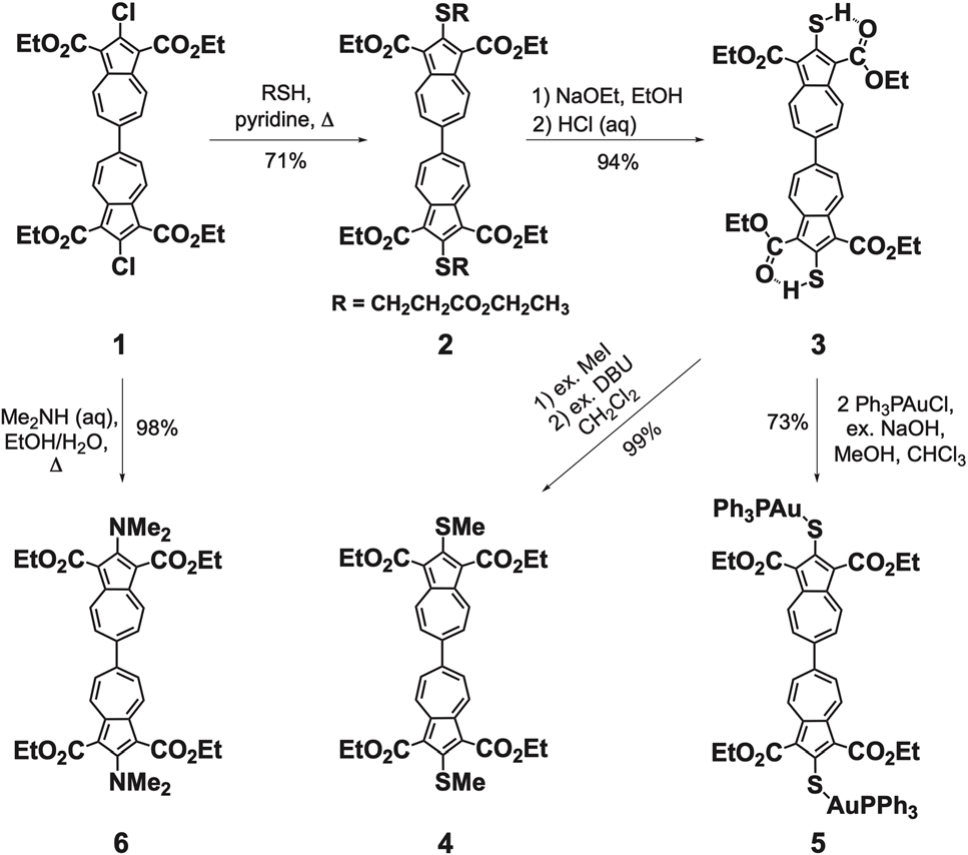
Syntheses of 6,6′-biazulenes symmetrically functionalized along their molecular axis.

Recognizing the untapped synthetic potential of 2,2′-dichloro-1,1′,3,3′-tetraethoxycarbonyl-6,6′-biazulene (1)^40^ for 2,2′-functionalization, we targeted the corresponding bis-mercapto-terminated derivative, a structural motif well-suited for interfacing π-conjugated linkers with metal atoms and surfaces. Refluxing 1 with ethyl 3-mercaptopropionate in pyridine displaced both chlorine substituents, furnishing the isolable, albeit somewhat oily, dark purple-brown intermediate 2 (Scheme 1). Subsequent base hydrolysis of 2, followed by acidification, afforded 2,2′-dimercapto-1,1′,3,3′-tetraethoxy-carbonyl-6,6′-biazulene (3) as a brick-red microcrystalline solid. Similar to 2-mercapto-1,3-diethoxycarbonylazulene,^43^ compound 3 engages in intramolecular C=O⋯H–S hydrogen bonding in both solution and solid state, as evidenced by a substantially deshielded mercapto ^1^H NMR resonance (*δ* = 7.78 ppm in CDCl_3_, Table S2), a low energy *ν*_SH_ stretch at 2474 cm^−1^, and two distinct *ν*_C=O_ bands at 1689 and 1653 cm^−1^ (KBr pellet). Deprotonation of 3 with excess 1,8-diazabicyclo[5.4.0]undec-7-ene (DBU) generated the deeply purple-coloured dianionic intermediate 3*, which underwent clean methylation to give microcrystalline, dark red 2,2′-methylthio-1,1′,3,3′-tetraethoxy-carbonyl-6,6′-biazulene (4) in quantitative yield.

**Fig. 3:**
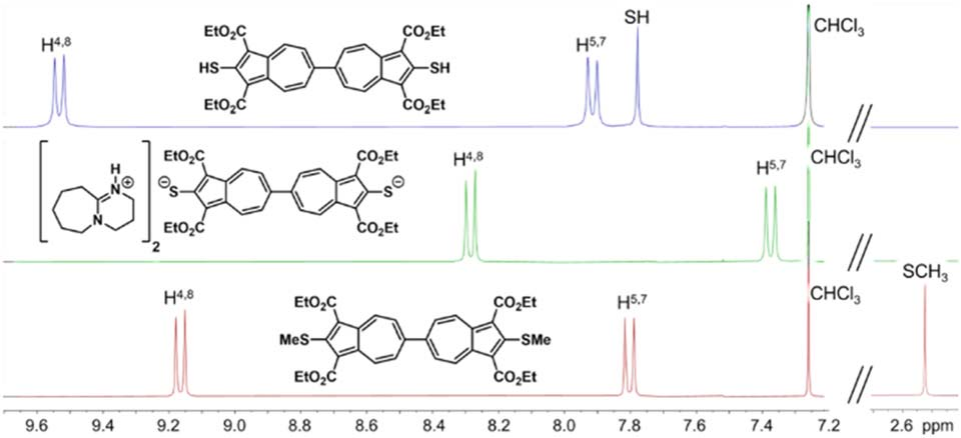
^1^H NMR spectra of 3, 3*, and 4 in CDCl_3_ at 25 °C.

The ^1^H NMR resonances for H^4,4′,8,8′^ and H^5,5′,7,7′^ in 3* appear substantially upfield relative to those of either 3 or 4 (Fig. 3). Although the direct synthesis of 4*via* nucleophilic substitution of the Cl termini in 1 using NaSMe (1 : 2) is conceptually feasible,^44^ the operational drawbacks associated with the notoriously malodorous NaSMe reagent render the three-step, high-yielding sequence 1 → 2 → 3 → 4 the more practical alternative. In contrast to the methylation, treatment of *in situ*-generated 3* with 2 equiv. of Ph_3_PAuCl produced a bright red solution of the bimetallic complex 5, which crystallized as a lustrous dark yellow solid (Scheme 1).

**Fig. 4:**
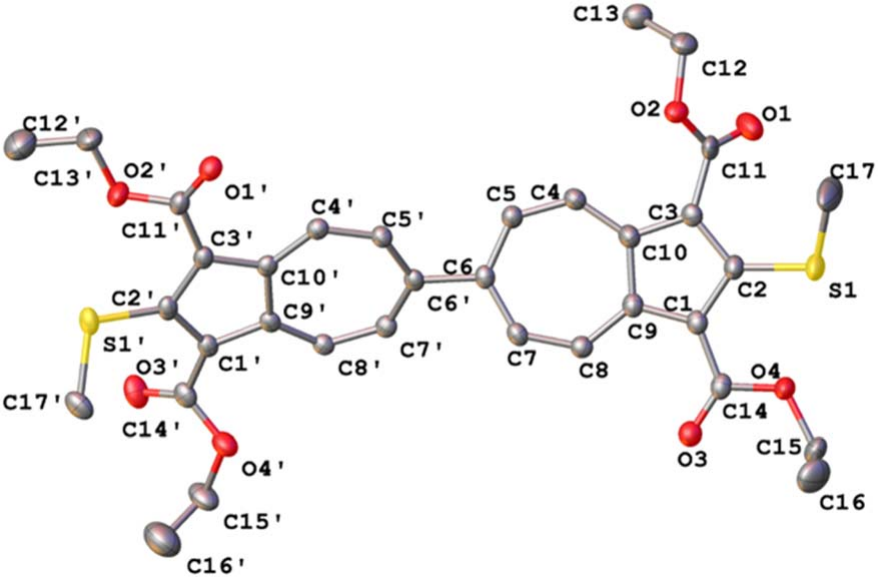
Molecular structure of 4 (50% thermal ellipsoids). This is the major contributor to the three-component disordered structure illustrated in Fig. S57. Selected bond distances (Å) and angles (°): S1′–C2′ 1.745(3), S1–C2 1.756(7), C6′–C6 1.502(3), C5–C6–C6′–C5′ 39.7(3), C17′–S1′–C2′–C1′ 26.9(3).

The solid-state structure of 4 reveals a three-component disorder of one of its SMe substituents with 50 : 31 : 19 positional occupancies (Fig. S57). The major conformer is depicted in Fig. 4. The interplanar angle between the two azulenic units of 39.7(3)° in 4 is the most acute among such parameters documented for all other crystallographically characterized 6,6′-biazulenes (typical range: 43.5–66.9°).^40^ This comparison, however, should be interpreted with caution, as such torsional parameters are inherently influenced by crystal-packing forces. The SMe groups themselves are mildly twisted relative to the adjoining 2-azulenyl units, with dihedral angles of *ca.* 26 ± 1° in the major conformer.

Inspired by Nozoe's classical preparation of 2-(*N*,*N*-dimethylamino)-1,3-diethoxycarbonylazulene,^45^ refluxing a purple solution of 1 with dimethylamine in EtOH/H_2_O cleanly delivered 2,2′-(*N*,*N*-dimethylamino)-1,1′,3,3′-tetraethoxy-carbonyl-6,6′-biazulene (6) upon workup. Unlike many other 6,6′-biazulenes, this blood-red, air- and thermally-stable solid displays surprisingly good solubility in most organic solvents.

**Scheme 2:**
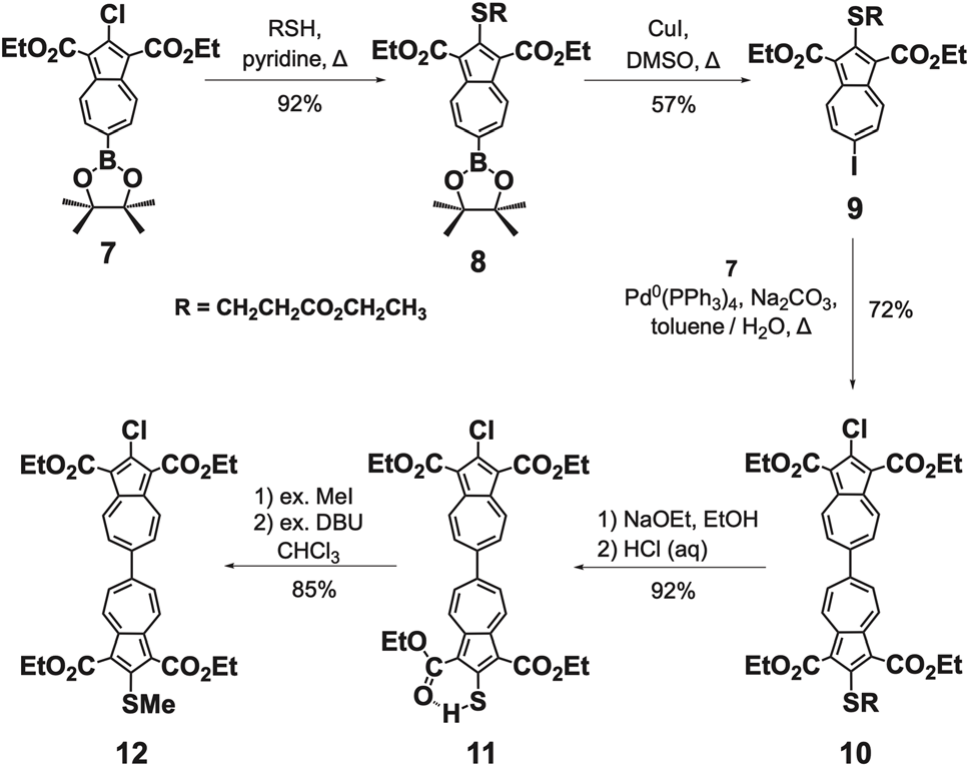
Syntheses of 6,6′-biazulenes asymmetrically functionalized along their molecular axis.

**Scheme 3:**
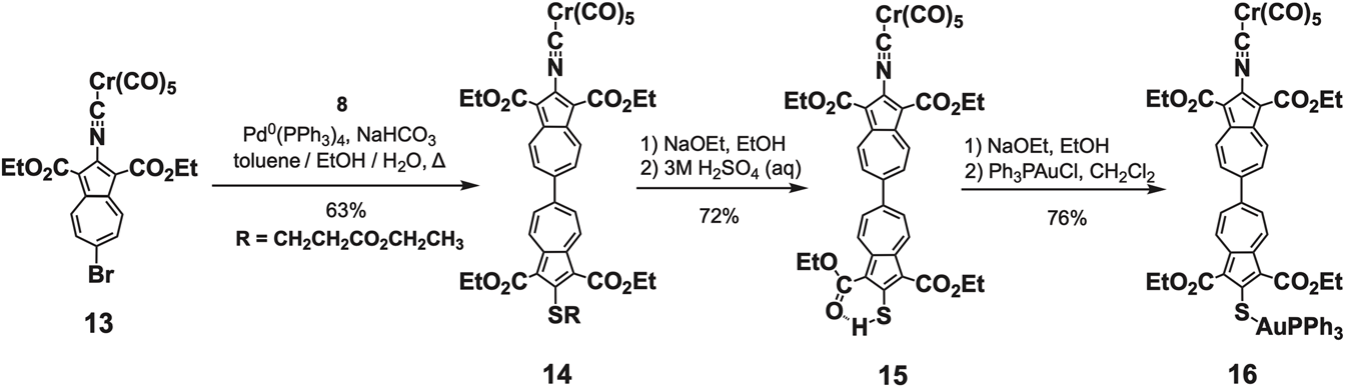
Synthesis and metalation of the 2-isocyano-2′-mercapto-6,6′-biazulenic motif.

Schemes 2 and 3 outline our modular access to asymmetrically 2,2′-functionalized 6,6′-biazulenes that capitalizes on the Suzuki–Miyaura cross-coupling approach.^46^ Purple-coloured 2-chloro-1,3-diethoxycarbonyl-6-pinacolato-borylazulene (7)^47^ served as a convenient precursor to access scarlet 2-ethoxycarbonylethylthio-1,3-diethoxycarbonyl-6-iodoazulene (9) *via* isolable dark purple 2-ethoxycarbonyl-ethylthio-1,3-diethoxycarbonyl-6-pinacolatoborylazulene intermediate (8). Coupling of 7 and 9 under Pd(PPh_3_)_4_ catalysis provided dark red 10, bearing SCH_2_CH_2_CO_2_Et and Cl substituents along the molecular axis. Further, sand-brown 2-mercapto-2′-chloro-6,6′-biazulene 11 and dark maroon 2-methylthio-2′-chloro-6,6′-biazulene 12 were prepared from 10 using the methods established above for the syntheses of 3 and 4, respectively (Scheme 2).

Syntheses of 6,6′-biazulenes featuring the [(–NC)Cr(CO)_5_] spectroscopic reporter at one of their termini are shown in Scheme 3. The Pd^0^-catalyzed cross coupling of bright orange (OC)_5_Cr(2-isocyano-1,3-diethoxycarbonyl-6-bromoazulene)^48^ with 8 afforded the corresponding deep brown biazulenic intermediate 14, which was then converted to brown 2-isocyano-2′-mercapto-6,6′-biazulene derivative 15. Species 15 represents an exceedingly scarce example of a compound containing both mercapto- and isocyano functionalities in the same molecule.^48^ Metalation of the SH terminus of 15 with Ph_3_PAuCl in the presence of NaOEt gave the plum-coloured heterobimetallic Cr^0^/Au^I^ complex 16.

### Electrochemical mapping of substituent effects

We begin by examining the electrochemical consequences of mercapto substitution. The –SH functional group is especially attractive because it provides access to thiolate junctions relevant to molecular scale charge transport.^49^ The half-wave potential of −1.31 V associated with the two-electron reduction of dimercaptobiazulene 3 translates, *via* the calibration plot in Fig. 2, to an effective Hammett constant *σ*_eff_ = −0.04 for the –SH substituent (Tables 1 and S4). This value represents a significant negative shift relative to the classical *σ*_p_(SH) = +0.15 reported in the literature,^26,28^ implying that the mercapto groups in 3 display subtle net electron-donating behavior rather than the weak electron-withdrawing character traditionally ascribed to S–H substituents. We attribute this discrepancy to intramolecular S–H⋯O=C hydrogen-bonding interactions^43,50^ present in 3. Consistent with this hypothesis, our DFT calculations indicate that disrupting such H-bonding interactions (modeled by rotating each S–H bond out of the plane of the corresponding azulenyl unit) lowers the energy of the biazulene LUMO (Fig. S59). To probe the role of the above hydrogen bonding experimentally, we replaced the hydrogen atoms of the –SH groups with methyl substituents. The resulting bis(methylthio)-substituted biazulene 4 undergoes a two-electron reduction at *E*_1/2_ = −1.30 V, corresponding to *σ*_eff_(SMe) = −0.03. This value closely matches *σ*_p_(SMe) = 0.0 originally reported by McDaniel^28^ and later compiled in the comprehensive Hammett parameter tables by Hansch,^26^ lending further support to our interpretation.

**Table 1:** Predicted Hammett parameters for –NMe_2_, –SH, –SAuPPh_3_, and –NCCr(CO)_5_ substituents based on the experimental *E*_1/2_ values for the 2-e^−^ reduction of 6,6′-biazulenes 3, 4, 5, 6, and 17 a

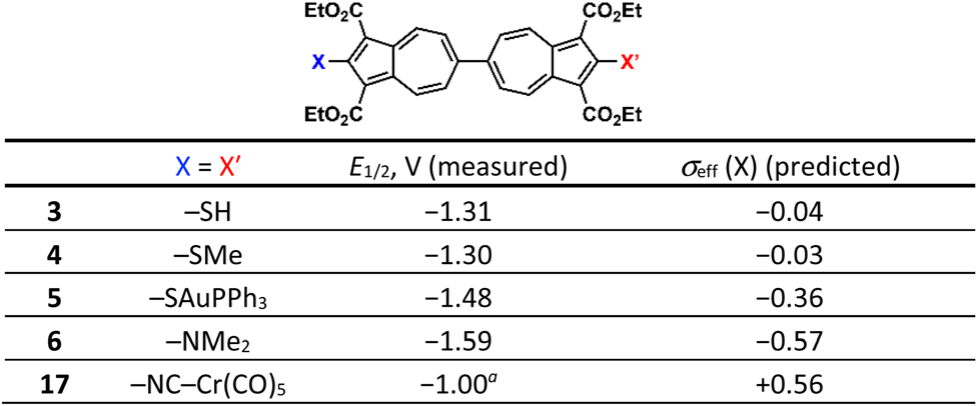

a Ref. 35.

**Fig. 5:**
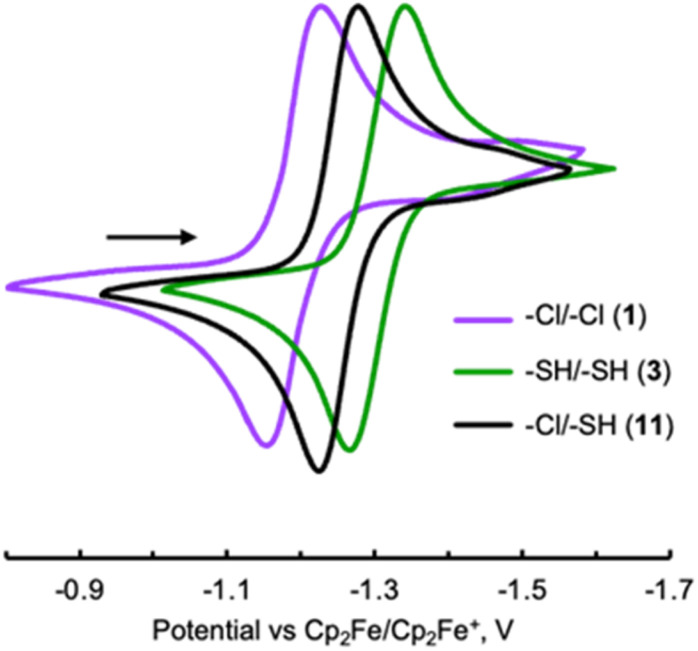
Cyclic voltammograms of 1 (purple), 3 (green), and 11 (black) in 0.1 M [^*n*^Bu_4_N]^+^[PF_6_]^−^/CH_2_Cl_2_*vs.* external Cp_2_Fe^0/+^ at 22 °C. Scan rate 100 mV s^−1^.

Importantly, the *E*_1/2_ potential associated with the 2-e^−^ reduction of asymmetrically –SH/–Cl functionalized biazulene 11 constitutes the average of the *E*_1/2_ values determined for the symmetrically substituted 2,2′-dimercapto and 2,2′-dichloro derivatives 1 and 3 (Fig. 5, Tables 1, 2, and S4). Similarly, the reduction of 12 featuring asymmetric –SMe/–Cl substitution is exactly half-way between those of 1 and 4 (Tables 1 and 2).

**Table 2:** Measured *vs.* predicted *E*_1/2_ values for the 2-e^−^ reduction of asymmetrically functionalized 6,6′-biazulenes 11, 12, 15, 16, and 18 a^,^^b^

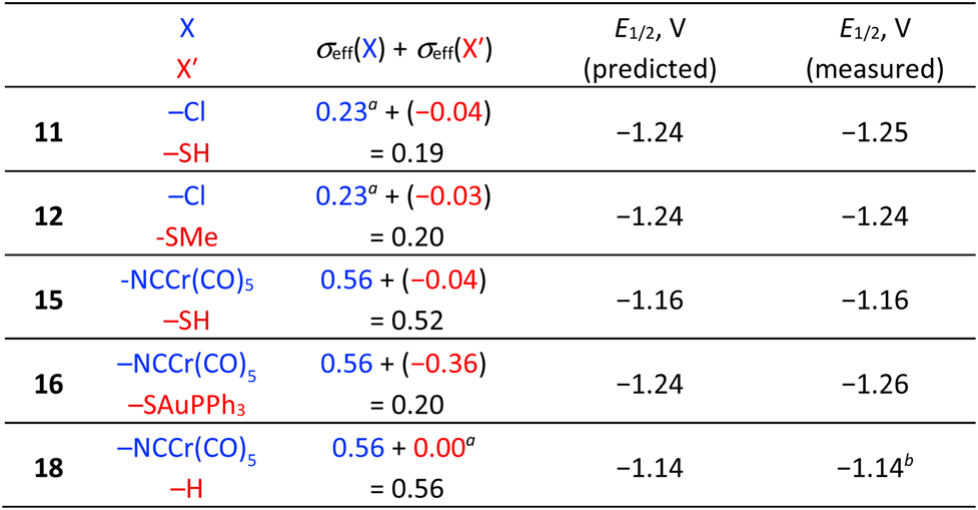

a Ref. 26.

b Ref. 35.

Linear free-energy relationships are rarely stress-tested against substituents that fall well outside the classical functional group palette. To assess the limits and transferability of the correlation uncovered in Fig. 2, we therefore extended our electrochemical Hammett analysis to 6,6′-biazulenes bearing “designer” substituents –SAuPPh_3_ and –NCCr(CO)_5_. These moieties are anticipated to exert very different net electron donating/withdrawing influences. Notably, our previous X-ray crystallographic analyses of several complexes featuring the (OC)_5_Cr(2-isocyano-1,3-diethoxycarbonylazulene) motif indicate that the C–N–C–Cr unit invariably assumes a nearly linear geometry, with minimal steric hindrance between the CO_2_Et and Cr(CO)_5_ fragments.^35,48,51–53^ The 6,6′-biazulene 5 featuring –SAuPPh_3_ termini undergoes reduction at *E*_1/2_ = −1.48 V, which allowed to extract the *σ*_eff_ value of −0.36 for the –SAuPPh_3_ substituent. This quantitative observation reflects the significantly enhanced net electron-donating character of –SAuPPh_3_*vs.* –SH (Tables 1 and S4). On the other hand, reduction of the 6,6′-biazulenic core terminated with –NCCr(CO)_5_ groups along its molecular axis in 17 occurs at *E*_1/2_ = −1.00 V,^35^ which yields *σ*_eff_[–NCCr(CO)_5_] = +0.56. This is consistent with the Cr(CO)_5_ fragment exerting a net electron withdrawing effect on the isocyano substituent (*cf*. *σ*_p_ = +0.49 (ref. 26) for the uncomplexed isocyano group). The 9 cm^−1^ hypsochromic shift of the *ν*_NC_ IR band upon complexation of free 2,2′-diisocyano-1,1′,3,3′-tetraethoxy-carbonyl-6,6′-biazulene (*ν*_NC_ = 2127 cm^−1^ in CH_2_Cl_2_) to form homobimetallic 17 (*ν*_NC_ = 2136 cm^−1^ in CH_2_Cl_2_) nicely mirrors the above conclusion as well.^34,35^

To test whether the *σ*_eff_ constants for –SAuPPh_3_ and –NCCr(CO)_5_ would abide the linear free-energy paradigm established by the calibration plot in Fig. 2, we considered the 2-e^−^ reduction behaviour of asymmetrically-functionalized 6,6′-biazulenes 15, 16, and 18 (Tables 2 and S4). The one-step, 2-e^−^ reduction of 15 bearing the –NCCr(CO)_5_ and –SH groups along its molecular axis occurs at −1.16 V. Gratifyingly, this value is precisely the average of the *E*_1/2_ potentials that we documented for the reductions of symmetrically-substituted congeners 3 and 17. Similarly, 18 undergoes reduction at *E*_1/2_ = −1.14 V, which is exactly in between the *E*_1/2_ values observed for the reductions of 17 and 1,1′,3,3′-tetraethoxycarbonyl-6,6′-biazulene.^40^ Finally, the heterobimetallic species 16 is reduced at *E*_1/2_ = −1.24 V—quite close to the predicted value of −1.26 V inferred from the redox profiles of homobimetallic 5 and 17 (Tables 1 and 2).

**Fig. 6:**
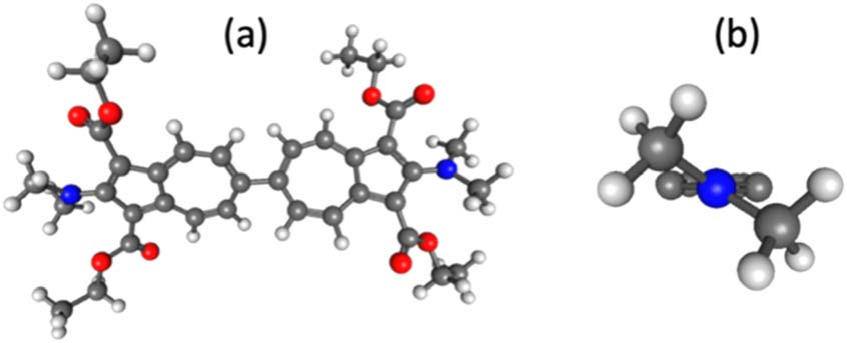
(a) DFT-optimized molecular structure of 6 (B3LYP/6-31+G*, CH_2_Cl_2_ medium). (b) A Newman projection of a Me_2_N-azulenyl moiety with the CO_2_Et groups truncated.

**Fig. 7:**
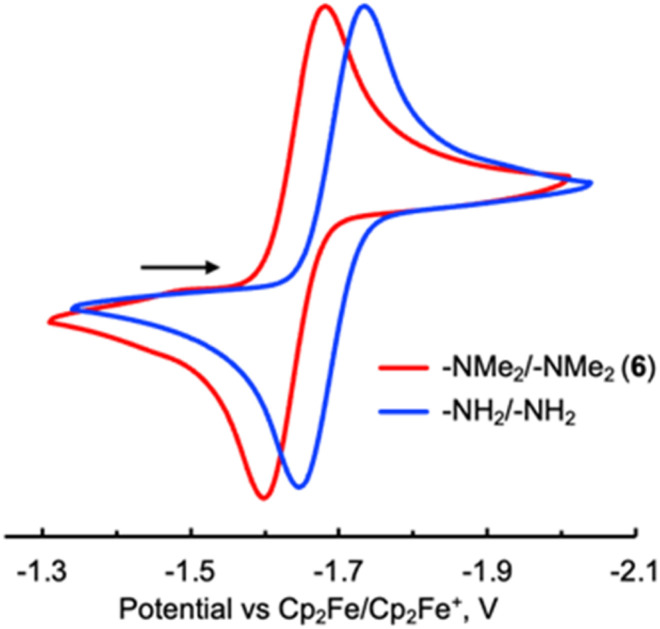
Cyclic voltammograms of 6 (red) and 2,2′-diamino-1,1′,3,3′-tetraethoxycarbonyl-6,6′-biazulene (blue) in 0.1 M [^*n*^Bu_4_N]^+^[PF_6_]^−^/CH_2_Cl_2_*vs.* external Cp_2_Fe^0/+^ at 22 °C. Scan rate 100 mV s^−1^.

We next turned to dimethylamino substitution, which represents a canonical class of strong π-electron donors in classical Hammett analyses.^26^ Examination of the –NMe_2_-functionalized biazulene allows a direct comparison between established substituent expectations and the experimentally observed redox response. This case is particularly intriguing because steric repulsion between the –NMe_2_ and –CO_2_Et groups in 6 twists the –NMe_2_ substituents out of optimal π-conjugation with the biazulenic core by *ca*. 32 ± 2° (Fig. 6), thereby likely diminishing their mesomeric electron-donating contributions to *σ*_eff_. The *E*_1/2_ potential associated with the 2-e^−^ reduction of the bis(*N*,*N*-dimethylamino) derivative 6 is 50 mV less negative than that documented for 2,2′-diamino-1,1′,3,3′-tetraethoxycarbonyl-6,6′-biazulene^34^ (Table 1 and Fig. 7). According to our calibration plot in Fig. 2, the effective Hammett *σ* constant for the –NMe_2_ substituent is predicted to be −0.57, which is identical to the *σ*_p_ value of −0.574 obtained by Gilman and Dunn^54^ through the alkaline hydrolysis of *p-N*,*N*-dimethylamino functionalized ethyl benzoate. However, the fact that 6 is easier to reduce than its diamino-functionalized congener may be perceived as counterintuitive in light of the typical view that –NMe_2_ is a stronger net electron donor than –NH_2_. This observation can be rationalized by two factors that affect *E*_1/2_ in opposite directions. First, intramolecular hydrogen-bonding interactions in 2,2′-diamino-1,1′,3,3′-tetraethoxycarbonyl-6,6′-biazulene render the –NH_2_ substituents more electron donating, which further raises the energy of this biazulene's LUMO. Second, the steric congestion illustrated in Fig. 6 somewhat weakens π-conjugation of the –NMe_2_ groups with the biazulenic scaffold. Such mode of steric and conformational attenuation of donor strength echoes Nozoe's observation that 2-(*N*,*N*-dimethylamino)-1,3-diethoxycarbonyl-azulene is inert toward electrophilic aromatic substitution with Br_2_ at the 6-position of the azulenic core, whereas 2-aminoazulene easily undergoes such bromination.^45,51^ Moreover, in the electrochemical context, Stahl, Hammes–Schiffer, and co-workers showed that bulky substituents at the 2,3- or 5,6-positions of quinones led to one-electron reduction potentials that are shifted to more positive values than anticipated from linear Hammett correlations.^6^

### Correlating ^13^C NMR signatures of the [(–NC)Cr(CO)_5_] reporter with remote substituent effects

In their earlier studies, Figueroa and co-workers explored the [Cr(CO)_5_] fragment as a spectroscopic handle for evaluating the *σ*-donor/π-acceptor balance of isocyanide ligands through inverse-linear correlations between the ^13^C chemical shifts of *trans*- or *cis*-carbonyl ligands and the corresponding *k*(*trans*-CO) or *k*(*cis*-CO) force constants in (OC)_5_Cr(CNR) complexes.^55^ Although this approach differentiated quite successfully between isocyanide ligands with strongly contrasting non-aryl-based substituents *R*, the authors acknowledged its limited utility in the context of functionalized aryl isocyanides, suggesting that the aryl moiety imposes a leveling effect on the substituent's intrinsic electronic character. Moreover, the precision of this analysis diminished when the variations in the nature of *R* were subtle, owing to inherent uncertainties in extracting *k*(CO) values from *ν*_CO_ vibrational data for *C*_4v_-symmetric M(CO_5_)L.^47,56^ More recently, we capitalized on the pronounced polarizability of azulene's π-system^57^ to develop a highly sensitive ^13^C NMR method that underpins the net *σ*-donor/π-acceptor character of functionalized 2-isocyano-azulenes from inverse-linear *δ*(^13^**C**O_trans_) or *δ*(^13^**C**O_cis_) *versus δ*(^13^**C**N) correlations for (OC)_5_Cr(2-isocyanoazulene) species.^48,52^ Building upon this foundation, we now demonstrate that the [(–NC)Cr(CO)_5_] unit serves as an exceptionally responsive ^13^C NMR reporter capable of discerning electron delocalization mediated by a linear 6,6′-biazulenic π-linker over *ca.* 2 nm range.

**Fig. 8:**
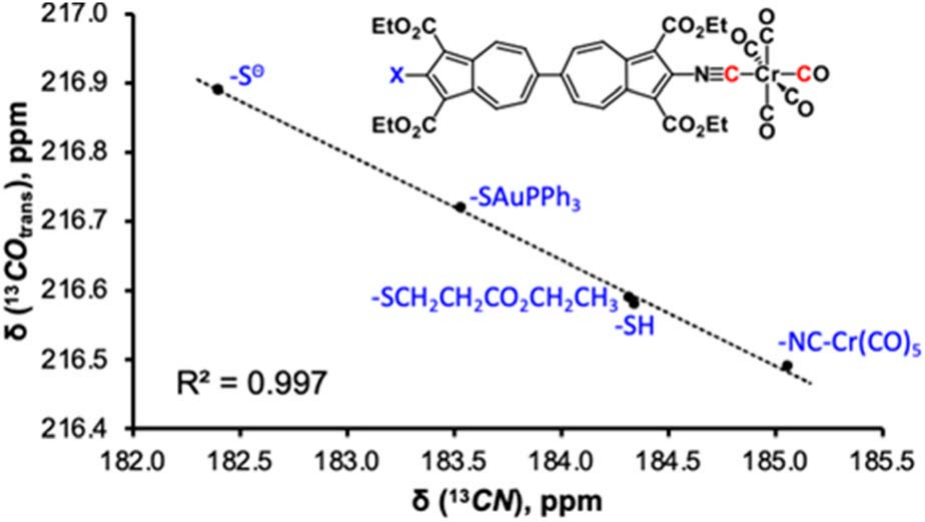
Plot of *δ*(^13^**C**O_trans_) *vs. δ*(^13^**C**N) chemical shifts (in CDCl_3_) for the [–NCCr(CO)_5_] moiety in complexes of functionalized 2-isocyanobiazulene ligands.

Table S3 and Fig. 8 and S31 illustrate how electronic perturbations introduced by substituents *X* (*X* = –S^−^, –SAuPPh_3_, –SCH_2_CH_2_CO_2_CH_2_CH_3_, –SH, –N≡CCr(CO)_5_) at one terminus of the linear 6,6′-biazulenic framework modulate the electron density at its other end featuring the [(–NC)Cr(CO)_5_] ^13^C NMR reporter. The ^13^C chemical shifts within the [(–NC)Cr(CO)_5_] moiety reflect electron richness of the Cr centre, which is influenced by the nature of the remote substituent *X*. A raise in the net electron-donating strength of *X* (–S^−^ > –SAuPPh_3_ > –SCH_2_CH_2_CO_2_CH_2_CH_3_ ≈ –SH > –N≡CCr(CO)_5_) decreases *δ*(^13^**C**N), with a concomitant increase in *δ*(^13^**C**O) due to enhanced Cr(dπ) → CO(pπ*) back-bonding.^55^ The *δ*(^13^**C**O_trans_) or *δ*(^13^**C**O_cis_) *versus δ*(^13^**C**N) inverse-linear correlations (Fig. 8 and S31, respectively) exhibit excellent linearity. Thus, the ^13^C NMR data quantitatively capture subtle, long-range substituent effects without relying on any vibrational metrics, establishing the [(–NC)Cr(CO)_5_] fragment as a highly sensitive reporter of π-mediated electron delocalization across the 6,6′-biazulenic scaffold.

### Self-assembly of mercaptobiazulene 15 on Au(111) surface

Immersion of four separate 1 × 1 cm^2^ gold-coated silicon substrates with predominant Au(111) surface orientation into 2 mM CHCl_3_ solutions of 15 for at least 24 hours produced self-assembled monolayers (SAMs) with ellipsometric thicknesses of 24.4 ± 2.1 Å, consistent with a terminal-upright molecular orientation and a “hollow-linear” Au–S binding mode^58^ (Fig. S60a). The calculated thickness of 23.7 Å for an idealized linear Au–S–C geometry agrees closely with this value.

Formation of the Au–S interface is evidenced by the disappearance of the weak *ν*_SH_ band of 15,^59^ and by pronounced changes in the *ν*_CO_ region. DFT calculations^48^ and a large body of experimental evidence^48,52,55^ suggests that for complexes [(OC)_5_Cr(CNR)] in an isotropic environment (*i.e.*, solution phase), one of the two IR-active *ν*_CO_ bands of *A*_1_ symmetry is typically obscured by a much more prominent *ν*_CO_ band of *E* symmetry. Upon approximately terminal-upright chemisorption, the *ν*_CO_(*E*) band, which reflects vibration of the *cis*-CO ligands within the *C*_4v_-symmetric [Cr(CO)_5_L] unit, diminishes markedly to uncover the *ν*_CO_(*A*_1_^(2)^) feature (Fig. S60b and c). The *ν*_CO_(*A*_1_^(2)^) band arises chiefly from vibration of the *trans*-CO unit.^48,52^ In addition, the relative intensity of the *ν*_CO_(*A*_1_^(1)^) band, which corresponds to the “breathing” mode of vibration of all five COs, decreases. These observations indicate preferential alignment of *cis*-CO oscillators parallel to the surface and suppression of the *ν*_CO_(*E*) absorption under the surface IR selection rules.^60^

**Fig. 9:**
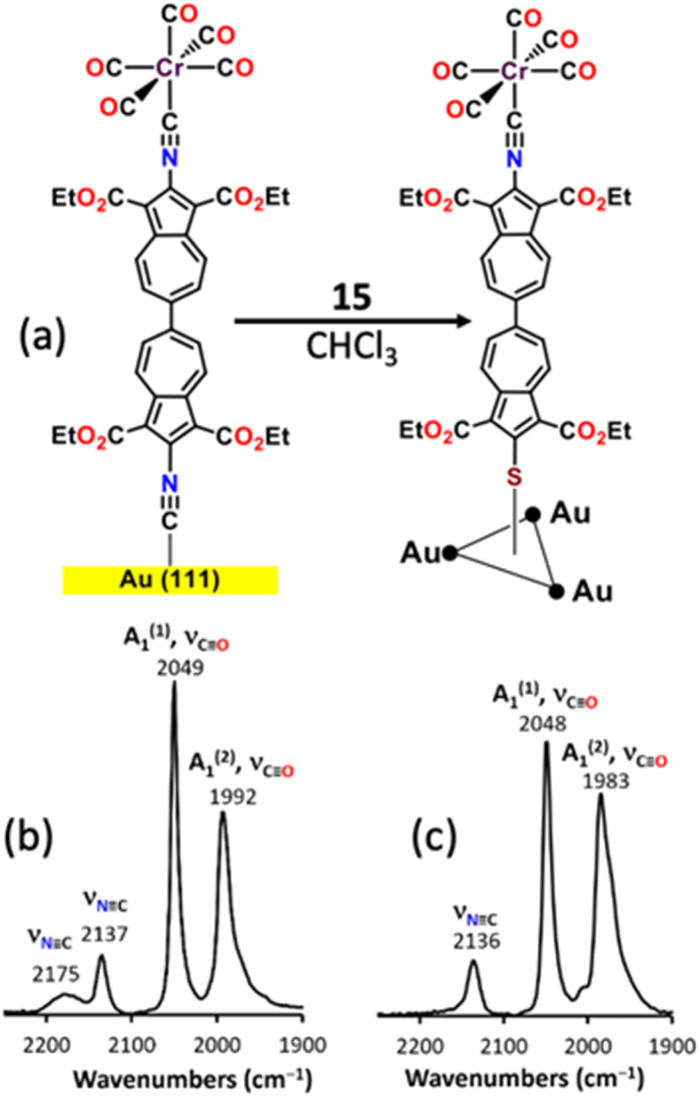
(a) Formation of the SAM film of 15 on the Au(111) surface *via* SAM displacement protocol. (b) RAIR spectrum of the SAM of the preformed isocyanide-anchored 6,6′-biazulenic monolayer on Au(111). (c) RAIR spectrum of the resulting S-anchored SAM film of 15 upon displacement.

When SAMs of 15 are instead generated *via* displacement of the isocyanide-anchored biazulenic film shown in Fig. 9a,^35^ the *ν*_CO_(*E*) band nearly disappears, and the broad *ν*_NC_ band at 2175 cm^−1^ (gold-bound isocyanide) vanishes altogether, confirming replacement of the chemisorbed isocyanobiazulene by mercaptobiazulene (Fig. 9b and c). Thus, the monolayer displacement route produces a more vertically-packed film, likely reflecting steric confinement of the vacated surface sites^61,62^ that are accessible only to vertically approaching molecules of 15.

Both *A*_1_-symmetric *ν*_CO_ bands of 15 undergo hypsochromic shifts upon SAM formation, with the *ν*_CO_(*A*_1_^(2)^) feature moving up by 23 cm^−1^ (Fig. S60b and c). This shift exceeds the experimental uncertainty in its *ν*_max_ value in the solution spectrum and suggests reduced Cr(dπ) → CO(pπ*) back-bonding in the SAM relative to free 15. The diminished electron richness of the Cr(0) centre arises from S(3p) → Au π-bonding, which enhances the net acceptor character^52^ of the isocyanobiazulene ligand. Thus, the thio-linked Au(111) surface acts as an electron-withdrawing moiety^35,48^ transmitting its effect through the biazulenic π-linker to the distal Cr(0) site, approximately 2 nm away. In contrast, the –SAuPPh_3_ fragment in the related heterobimetallic complex 16, with the anticipated bent C–S–Au angle,^50^ behaves as a net electron donor (Fig. 8). The above observations demonstrate that 6,6′-biazulene can serve as a molecular relay, communicating spectroscopically discernible electronic perturbations from a metallic surface to a remote metal centre.

## Conclusions

A family of previously inaccessible 6,6′-biazulenes, both symmetrically and asymmetrically functionalized along their molecular axis, was synthesized in this work. The 2,2′-dichloro-1,1′,3,3′-tetraethoxycarbonyl-6,6′-biazulene precursor proved to be a versatile synthon for accessing symmetrically functionalized derivatives through substitution at the 2,2′-positions. Of particular interest for the realm of π-conducting materials is the hitherto unknown 2,2′-dimercapto-1,1′,3,3′-tetraethoxycarbonyl-6,6′-biazulene 3, which combines extended π-conjugation with surface anchoring capability. On the other hand, Suzuki–Miyaura cross-coupling provided a modular route to asymmetrically-functionalized 6,6′-biazulenes, permitting installation of electronically distinct substituents at the 2,2′-termini of the 6,6′-biazulenic framework. The calibration plot correlating the half-wave potential (*E*_1/2_) for the one-step, 2-e^−^ reversible reduction of 2,2′-functionalized 6,6′-biazulenes with the sum of Hammett *σ*_p_ constants led to straightforward extraction of effective *σ* parameters for substituents lacking conventional *σ*_p_ descriptors. Using this calibration, a dual Hammett analysis was implemented: a reverse approach to derive effective *σ* constants electrochemically, followed by a direct correlation to predict reduction potentials of newly synthesized 6,6′-biazulenic derivatives. The excellent predictive accuracy of such bidirectional validation underscored the quantitative reliability of the linear free-energy relationships governing our 6,6′-biazulenic Hammett analyses. Our electrochemical analysis captured the effect of intramolecular C=O⋯H–S hydrogen bonding, which rendered the effective *σ* parameter for the mercapto group more negative than the commonly accepted *σ*_p_ value, reflecting its increased electron-donating capacity imparted through such non-Pauling H-bonding interaction.^63^ Furthermore, considering the –NMe_2_ substituent as a case study afforded direct access to its effective Hammett constant. A mild steric repulsion with the ethoxycarbonyl groups in 6 diminishes π-conjugative donation of –NMe_2_, surprisingly leading to *σ*_eff_(NMe_2_) > *σ*_eff_(NH_2_). Finally, we demonstrated how the [–NCCr(CO)_5_] fragment serves as a highly responsive ^13^C NMR and RAIR reporter, providing complementary insight into electron delocalization across the 6,6′-biazulenic π-linker.

Taken together, the synthetic, electrochemical, and spectroscopic findings reported herein offer an experimentally robust platform for systematically quantifying substituent effects. This platform transcends the classical benzenoid *σ*_p_ paradigm while remaining numerically anchored to it by design. By further enabling predictive mapping of electronic influence, our approach expands the palette for tailored design of redox-active molecules, π-linked materials, and electronically coupled interfaces. In a broader context, the 1,1′,3,3′-tetraethoxy-carbonyl-6,6′-biazulenic platform is most valuable for interrogating any substituents whose electronic influence is dominated by inductive effects rather than mesomeric π-conjugation, as well as for π-conjugating substituents that experience minimal steric interference. Our current efforts are directed toward the development of a second-generation 6,6′-biazulenic framework designed to avoid intramolecular hydrogen bonding and steric contributions, thereby further refining the extraction of effective Hammett *σ* values.

## Author contributions

Conceptualization: M. V. B.; synthesis, characterization, and data analysis: J. A. M., S. R. K, J. C. A., R. C. S., and C. R. W.; surface studies: M. K. D. and C. L. B.; crystal structure solution and refinement: D. E. J.; computational work: R. F. and W. H. T.; supervision: M. V. B., C. L. B., and W. H. T.; original draft preparation: M. V. B., J. A. M., and S. R. K. All authors were involved in reviewing and editing the manuscript and have agreed to its published version.

## Conflicts of interest

There are no conflicts to declare.

## Supplementary Material

RA-016-D6RA00120C-s001

RA-016-D6RA00120C-s002

## Data Availability

CCDC 2482367 (4) contains the supplementary crystallographic data for this paper.^64^ Full experimental details, characterization and computational data are provided in the supplementary information (SI). Supplementary information is available. See DOI: https://doi.org/10.1039/d6ra00120c.
