# Supplementary material for: 6,6′-Biazulenic core as a platform for unlocking Hammett constants via electrochemical free-energy relationships
Source: RSC Adv. 2026 Feb 11;16(9):8371–9. doi: 10.1039/d6ra00120c (PMC12893891; doi:10.1039/d6ra00120c)
Supplement: RA-016-D6RA00120C-s001 [file RA-016-D6RA00120C-s001.pdf]

This journal is © The Royal Society of Chemistry 2026

Supplementary Information for:

6,6'-Biazulenic core as a platform for unlocking Hammett constants via electrochemical free energy relationships

Joseph A. Mandigo,‡[a] Shaun R. Kelsey,‡[a] Jason C. Applegate,[a] Rene C. Sabala,[a] Monisola K. Dairo,[a] Carben R. Weghorn,[a] Raina Fair,[a] Cindy L. Berrie,[a] Ward H. Thompson,*[a] Daron E. Janzen,[b] and Mikhail V. Barybin*[a]*

[a]Department of Chemistry, University of Kansas, Lawrence, KS 66045, USA.

[b]Department of Chemistry and Biochemistry, St. Catherine University, St. Paul, MN 55105, USA

To whom correspondence should be addressed. E-mail: mbarybin@ku.edu (M.V.B.), cberrie@ku.edu (C.L.B.), wthompson@ku.edu (W.H.T.)

Table of Contents

A. Synthetic Procedures 7

A1. General Procedures, Starting Materials, and Equipment 7

A2. Synthesis of **2** 8

A3. Synthesis of **3** 9

A4. Generation of **3*** 10

A5. Synthesis of **4** 10

A6. Synthesis of **5** 11

A7. Synthesis of **6** 12

A8. Synthesis of **8** 12

A9. Synthesis of **9** 13

A10. Synthesis of **10** 14

A11. Synthesis of **11** 15

A12. Synthesis of **12** 16

A13. Synthesis of **14** 16

A14. Synthesis of **15** 17

A15. Generation of **15*** 18

A16. Synthesis of **16** 19

B. NMR Data 21

Figure S1. ^{1}H HMR (500 MHz, $CDCl_3$, 25°C) of **2** 21

Figure S2. ^{13}C HMR (126 MHz, $CDCl_3$, 25°C) of **2** 22

Figure S3. ^{1}H HMR (500 MHz, $CDCl_3$, 25°C) of **3** 23

Figure S4. ^{13}C HMR (126 MHz, $CDCl_3$, 25°C) of **3** 24

Figure S5. ^{1}H HMR (400 MHz, $CDCl_3$, 25°C) of **3*** 25

Figure S6. ^{1}H HMR (400 MHz, $CDCl_3$, 25°C) of **4** 26

Figure S7. ^{13}C HMR (126 MHz, $CDCl_3$, 25°C) of **4** 27

Figure S8. ^{1}H HMR (500 MHz, $CDCl_3$, 25°C) of **5** 28

Figure S9. ^{13}C HMR (126 MHz, $CDCl_3$, 25°C) of **5** 29

Figure S10. $^{31}P\{^{1}H\}$ NMR (202 MHz, $CDCl_3$) spectrum of **5** 30

Figure S11. ^{1}H HMR (500 MHz, $CDCl_3$, 25°C) of **6** 31

Figure S12. ^{13}C HMR (126 MHz, $CDCl_3$, 25°C) of **6** 32

Figure S13. ^{1}H HMR (500 MHz, $CDCl_3$, 25°C) of **8** 33

Figure S14. ^{13}C HMR (126 MHz, $CDCl_3$, 25°C) of **8** 34

Figure S15. ^{1}H HMR (400 MHz, $CDCl_3$, 25°C) of **9** 35

Figure S16. ^{13}C HMR (126 MHz, $CDCl_3$, 25°C) of **10** 36

Figure S17. ^{1}H HMR (500 MHz, $CDCl_3$, 25°C) of **11** 37

Figure S18. ^{13}C HMR (126 MHz, $CDCl_3$, 25°C) of **11** 38

Figure S19. ^{1}H HMR (500 MHz, $CDCl_3$, 25°C) of **12** 39

Figure S20. ^{13}C HMR (126 MHz, $CDCl_3$, 25°C) of **12** 40

Figure S21. ^{1}H HMR (400 MHz, $CDCl_3$, 25°C) of **14** 41

Figure S22. ^{13}C HMR (126 MHz, $CDCl_3$, 25°C) of **14** 42

Figure S23. ^{1}H HMR (400 MHz, $CDCl_3$, 25°C) of **15** 43

Figure S24. ^{13}C HMR (126 MHz, $CDCl_3$, 25°C) of **15** 44

Figure S25. ^{13}C HMR (126 MHz, $CDCl_3$, 25°C) of **15*** 45

Figure S26. ^{1}H HMR (400 MHz, $CDCl_3$, 25°C) of **16** 46

Figure S27. ^{13}C HMR (126 MHz, $CDCl_3$, 25°C) of **16** 47

Figure S28. $^{31}P\{^{1}H\}$ NMR (162 MHz, $CDCl_3$) spectrum of **16** 48

Figure S29. ^{1}H HMR (400 MHz, $CDCl_3$, 25°C) of 2-aminoazulene. 49

Figure S30. ^{1}H HMR (400 MHz, $CDCl_3$, 25°C) of 2-amino-1,3-diethoxycarbonylazulene. .. 50

Table S1. ^{1}H NMR chemical shifts for the amino group(s) of 2-aminoazulenes in $CDCl_3$. 51

Table S2. ^{1}H NMR chemical shifts for the mercapto group(s) of 2-mercaptoazulenes in $CDCl_3$. 51

Table S3. ^{13}C NMR chemical shifts for the [NC-Cr$(CO)_5$] moiety in **14**, **15**, **15***, **16**, and **17** in $CDCl_3$. 52

Figure S31. Plot of $\delta(^{13}CO_{cis})$ vs. $\delta(^{13}CN)$ chemical shifts (in $CDCl_3$) for the [NC-Cr$(CO)_5$] moiety in complexes of functionalized 2-isocyanobiazulene ligands. 53

C. HRMS 54

Figure S32. Positive-ion ESI mass spectrum of **2**. 54

Figure S33. Positive-ion ESI mass spectrum of **4**. 55

Figure S34. Positive-ion ESI mass spectrum of **5**. 56

Figure S35. Positive-ion ESI mass spectrum of **6**. 57

Figure S36. Positive-ion ESI mass spectrum of **8** 58

Figure S37. Positive-ion ESI mass spectrum of **9**. 59

Figure S38. Positive-ion ESI mass spectrum of **10**. 60

Figure S39. Negative-ion ESI mass spectrum of **11**. 61

Figure S40. Positive-ion ESI mass spectrum of **12**. 62

Figure S41. Positive-ion ESI mass spectrum of **14**. 63

Figure S42. Negative-ion ESI mass spectrum of **15**. 64

D. Electrochemistry 65

Figure S43. (a) Cyclic voltammogram of **3** in 0.1 M $[^nBu_4N]^+[PF_6]^-/CH_2Cl_2$ vs. external $Cp_2Fe^{0/+}$ at 22°C (scan rate = 100 mV/s). (b) Randles-Sevcik graph of peak currents versus square root of the scan rate for **3** in 0.1 M $[^nBu_4N]^+[PF_6]^-/CH_2Cl_2$. 65

Figure S44. (a) Cyclic voltammogram of **4** in 0.1 M $[^nBu_4N]^+[PF_6]^-/CH_2Cl_2$ vs. external $Cp_2Fe^{0/+}$ at 22°C (scan rate = 100 mV/s). (b) Randles-Sevcik graph of peak currents versus square root of the scan rate for **4** in 0.1 M $[^nBu_4N]^+[PF_6]^-/CH_2Cl_2$. 65

Figure S45. (a) Cyclic voltammogram of **5** in 0.1 M $[^nBu_4N]^+[PF_6]^-/CH_2Cl_2$ vs. external $Cp_2Fe^{0/+}$ at 22°C (scan rate = 100 mV/s). (b) Randles-Sevcik graph of peak currents versus square root of the scan rate for **5** in 0.1 M $[^nBu_4N]^+[PF_6]^-/CH_2Cl_2$. 66

Figure S46. (a) Cyclic voltammogram of **6** in 0.1 M $[^nBu_4N]^+[PF_6]^-/CH_2Cl_2$ vs. external $Cp_2Fe^{0/+}$ at 22°C (scan rate = 100 mV/s). (b) Randles-Sevcik graph of peak currents versus square root of the scan rate for **6** in 0.1 M $[^nBu_4N]^+[PF_6]^-/CH_2Cl_2$. 66

Figure S47. (a) Cyclic voltammogram of **11** in 0.1 M $[^nBu_4N]^+[PF_6]^-/CH_2Cl_2$ vs. external $Cp_2Fe^{0/+}$ at 22°C (scan rate = 100 mV/s). (b) Randles-Sevcik graph of peak currents versus square root of the scan rate for **11** in 0.1 M $[^nBu_4N]^+[PF_6]^-/CH_2Cl_2$. 67

Figure S48. (a) Cyclic voltammogram of **12** in 0.1 M $[^nBu_4N]^+[PF_6]^-/CH_2Cl_2$ vs. external $Cp_2Fe^{0/+}$ at 22°C (scan rate = 100 mV/s). (b) Randles-Sevcik graph of peak currents versus square root of the scan rate for **12** in 0.1 M $[^nBu_4N]^+[PF_6]^-/CH_2Cl_2$. 67

Figure S49. (a) Cyclic voltammogram of **15** in 0.1 M $[^nBu_4N]^+[PF_6]^-/CH_2Cl_2$ vs. external $Cp_2Fe^{0/+}$ at 22°C (scan rate = 100 mV/s). (b) Randles-Sevcik graph of peak currents versus square root of the scan rate for **15** in 0.1 M $[^nBu_4N]^+[PF_6]^-/CH_2Cl_2$. 68

Figure S50. (a) Cyclic voltammogram of **16** in 0.1 M $[^nBu_4N]^+[PF_6]^-/CH_2Cl_2$ vs. external $Cp_2Fe^{0/+}$ at 22°C (scan rate = 100 mV/s). (b) Randles-Sevcik graph of peak currents versus square root of the scan rate for **16** in 0.1 M $[^nBu_4N]^+[PF_6]^-/CH_2Cl_2$. 68

Figure S51. (a) Cyclic voltammogram of 2,2’-diamino-1,1’,3,3’-tetraethoxycarbonyl-6,6’-biazulene in 0.1 M $[^nBu_4N]^+[PF_6]^-/CH_2Cl_2$ vs. external $Cp_2Fe^{0/+}$ at 22°C (scan rate = 100 mV/s). (b) Randles-Sevcik graph of peak currents versus square root of the scan rate for 2,2’-diamino-1,1’,3,3’-tetraethoxycarbonyl-6,6’-biazulene in 0.1 M $[^nBu_4N]^+[PF_6]^-/CH_2Cl_2$. 69

Table S4. Cyclic voltametric data pertaining to the two-electron reduction of 2,2’-functionalized 6,6’-biazulenes. 70

E. Electronic Absorption Spectra 71

Figure S52. Electronic absorption spectra of **3**, **3***, **4**, and **5** in CH_2Cl_2 at 22°C. 71

Figure S53. Electronic absorption spectrum of **6** in CH_2Cl_2 at 22°C. 71

Figure S54. Electronic absorption spectra of **11** and **12** in CH_2Cl_2 at 22°C. 72

Figure S55. Electronic absorption spectrum of **14** in CH_2Cl_2 at 22°C. 72

Figure S56. Electronic absorption spectra of **15**, **15***, and **16** in CH_2Cl_2 at 22°C. 73

F. X-ray Crystallographic Studies 74

F1. Experimental and Refinement Model Description 74

Table S5. Crystal data and structure refinement for **4**. 77

Table S6. Fractional Atomic Coordinates ($\times10^4$) and Equivalent Isotropic Displacement Parameters ($Å^2\times10^3$) for **4**. U_{eq} is defined as 1/3 of the trace of the orthogonalised U_{IJ} tensor. 77

Table S7. Anisotropic Displacement Parameters ($Å^2\times10^3$) for **4**. The Anisotropic displacement factor exponent takes the form: $-2\pi^2[h^2a^{*2}U_{11}+2hka^*b^*U_{12}+\ldots]$. 79

Table S8. Bond Lengths for **4**. 81

Table S9. Bond Angles for **4**. 82

Table S10. Torsion Angles for **4**. 83

Table S11. Hydrogen Atom Coordinates ($Å\times10^4$) and Isotropic Displacement Parameters ($Å^2\times10^3$) for **4**. 85

Figure S57. (a) Positional disorder of one of the substituted five-membered rings within **4** modeled/refined by invoking 50%:31%:19% fragment occupancies. (b–d) individual conformers of the three-component disorder. 87

Figure S58. The unit cell of **4**........ 88

G. Computational Work 89

G1. Calculation Details........ 89

Table S12. Cartesian coordinates of the atoms in the DFT-optimized structure of **3**........ 89

Table S13. Cartesian coordinates of the atoms in the DFT-optimized structure of 3 in which the O…H–S hydrogen bonds were disrupted by restricting the H–S–C–C dihedral angles to 90°........ 91

Figure S59. (a) LUMO of the DFT-optimized molecular structure of **3**. (b) LUMO of the DFT-optimized molecular structure of **3** with each S–H bond restricted to be rotated 90° out of the plane of the corresponding azulenyl unit........ 93

Table S14. Cartesian coordinates (in Å) of the atoms in the DFT-optimized (B3LYP/6-31+G*) structure of **6**. 93

H. Surface Studies 96

H1. Self-assembled monolayer films (SAMs) of 15 on Au(111) surfaces........ 96

H2. Reflection Absorption Infrared (RAIR) spectroscopic measurements 96

H3. Optical ellipsometry........ 96

Figure S60. (a) Direct self-assembly of mercaptobiazulene **15** on the Au(111) surface from its solution in $CHCl_3$. (b) FTIR spectrum of **15** in $CHCl_3$. (c) RAIR spectrum of the resulting S-anchored SAM. 97

References........98

A. Synthetic Procedures

A1. General Procedures, Starting Materials, and Equipment

Unless otherwise stated, synthetic operations were performed without protection from air. Pyridine was distilled over KOH. Dimethyl sulfoxide (DMSO) and CH_2Cl_2 were distilled over CaH_2. $CHCl_3$ and $CDCl_3$ were distilled over P_2O_5. Methanol and ethanol were distilled over Mg turnings. Davisil (200–425 mesh, type 60 Å) silica gel was used for chromatographic purifications. Infrared spectra were recorded on a PerkinElmer Spectrum 100 FTIR spectrometer with solution samples sealed in 0.1 mm NaCl cells or solid-state samples as KBr pellets. NMR samples were analyzed on Bruker Avance III HD 400 MHz or Avance III 500 MHz spectrometers. ^{1}H and ^{13}C NMR chemical shifts are given with reference to residual solvent resonances relative to $SiMe_4$. ^{31}P NMR chemical shifts are referenced to 85% aqueous H_3PO_4 external standard. Electronic spectra were recorded at 22±3°C using a Shimadzu UV-3600 UV-Vis-NIR spectrophotometer or a Varian Cary 50 Bio spectrophotometer. Cyclic voltammetry experiments were conducted at room temperature using an EPSILON (Bioanalytical Systems Inc., West Lafayette, IN) electrochemical workstation inside a Vacuum Atmospheres dry box filled with argon. A solution of 0.1 M $[^nBu_4N][PF_6]$ in CH_2Cl_2 was used as the supporting electrolyte. A three-component system consisting of a glassy carbon working electrode, a platinum-wire auxiliary electrode, and a glass encased non-aqueous Ag/AgCl reference electrode was employed. The reported potentials were determined at a scan rate of 100 mV/s. IR compensation of 80% was implemented in processing the electrochemical data for **1, 3, 4, 6, 11,** and **12**. Melting points are uncorrected and were determined for samples sealed in capillary tubes. High-resolution electrospray ionization mass spectrometry (ESI-MS) experiments were performed using an LCT Premier MicroMass electrospray time-of-flight instrument. Elemental analyses were carried out by Micro-Analysis, Inc., Wilmington, Delaware, or by Dr. William Brennessel at the CENTC Elemental Analysis Facility at the University of Rochester.

2,2’-Dichloro-1,1',3,3'-tetraethoxycarbonyl-6,6’-biazulene (**1**),[1] 2-chloro-1,3-diethoxy carbonyl-6-pinacolatoborylazulene (**7**),[2] $[Cr(CO)_5]$(2-isocyano-6-bromo-1,3-diethoxy carbonylazulene) (**13**),[3] and $Au(PPh_3)Cl$[4] were synthesized according to literature procedures.

Commercial CuI was purified by dissolving the powder in a saturated aqueous solution of KI and filtering off impurities. All other reagents and solvents were obtained from commercial sources and used as received.

A2. Synthesis of **2**

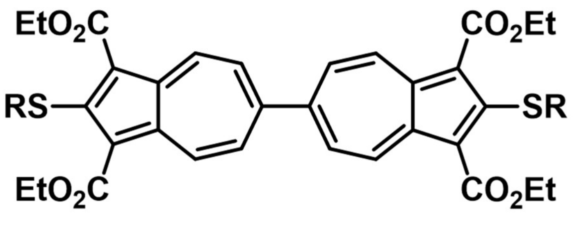

Neat ethyl-3-mercaptopropionate (1.81 mL, 14.2 mmol) was added to a bright purple solution of **1** (2.81 g, 4.59 mmol) in 150 mL of pyridine, and the resulting mixture was refluxed for 45 minutes with stirring. All solvent was then removed under vacuum and the oily residue was washed with hexanes (6 × 50 mL). The oil was subjected to column chromatography on silica gel (*ca*. 20 cm x 3 cm) using 30:1 CH_2Cl_2 / ethyl acetate eluent. The second band, dark brown in color, was collected. After solvent removal and drying of the residue at 10^{-2} torr, **2** (2.82 g, 3.48 mmol) was isolated as a viscous dark purple-brown solid in a 71% yield. HRMS (m/z, ES+): Calcd for $C_{42}H_{46}O_{12}S_2Na^+$: 829.2328, found 829.2408 $[M+Na]^+$. ^{1}H NMR (500 MHz, $CDCl_3$): δ 9.15 (d, $^3J_{HH}$ = 10.2 Hz, 4H, $H^{4,4',8,8'}$), 7.77 (d, $^3J_{HH}$ = 10.2 Hz, 4H, $H^{5,5',7,7'}$), 4.47 (q, $^3J_{HH}$ = 7.1 Hz, 8H, CH_2), 4.07 (q, $^3J_{HH}$ = 7.1 Hz, 4H, CH_2), 3.31 (t, $^3J_{HH}$ = 7.7 Hz, 4H, CH_2), 2.57 (t, $^3J_{HH}$ = 7.7 Hz, 4H, CH_2), 1.44 (t, $^3J_{HH}$ = 7.1 Hz, 12H, CH_3), 1.21 (t, $^3J_{HH}$ = 7.1 Hz, 6H, CH_3) ppm. $^{13}C\{^1H\}$ NMR (126 MHz, $CDCl_3$): δ 171.39 (C=O), 165.23 (C=O), 153.74, 152.16, 140.77, 134.71, 131.03, 119.27 (azulenic C-atoms), 61.12, 60.77, 34.56, 30.60 (CH_2), 14.49, 14.19 (CH_3) ppm.

A3. Synthesis of **3**

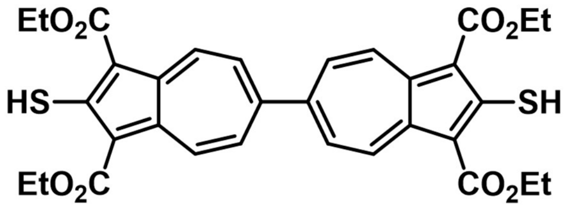

Sodium metal (0.82 g, 35.67 mmol), cut into small pieces, was carefully added to a solution of **2** (2.81 g, 3.48 mmol) in 200-proof ethanol (175 mL) at 0°C with vigorous stirring. Upon consumption of all sodium metal, the resulting purple mixture was warmed to 55°C and stirred for 2 hours. After cooling the reaction mixture to room temperature, 12 M aqueous HCl was added dropwise until acidic pH was established. The mixture was then poured into cold deionized water (800 mL), and the organic product was extracted with $CHCl_3$ (4 × 120 mL). The combined extracts were washed with water (5 × 80 mL) and dried over anhydrous Na_2SO_4. The drying agent was filtered off and the filtrate was concentrated to dryness under reduced pressure. The resulting red powder was washed with pentane and dried at 10^{-2} torr to afford **3** (2.02 g, 3.33 mmol) in a 94% yield. Even though **3** is sufficiently air-stable for reaction workup purposes, it should be stored under O_2-free atmosphere. Mp: 233–235°C. Anal. Calcd for $C_{32}H_{30}O_8S_2$: C, 63.35; H, 4.98; Found: C, 62.91; H, 4.82. IR (KBr): ν_{SH} 2474, $\nu_{C=O}$ 1689, 1653 (esters) cm^{-1}. IR (CH_2Cl_2): ν_{SH} 2583, $\nu_{C=O}$ 1687, 1662 (esters) cm^{-1}. ^{1}H NMR (500 MHz, $CDCl_3$): δ 9.53 (d, $^3J_{HH}$ = 11.1 Hz, 4H, $H^{4,4',8,8'}$), 7.91 (d, $^3J_{HH}$ = 11.1 Hz, 4H, $H^{5,5',7,7'}$), 7.77 (s, 2H), 4.55 (q, $^3J_{HH}$ = 7.1 Hz, 8H, CH_2), 1.53 (t, $^3J_{HH}$ = 7.1 Hz, 12H, CH_3) ppm. $^{13}C\{^1H\}$ NMR (126 MHz, $CDCl_3$): δ 165.95 (C=O), 157.13, 152.77, 143.01, 134.67, 132.50, 114.65 (azulenic C-atoms), 60.99 ($\underline{C}H_2CH_3$), 14.71 ($CH_2\underline{C}H_3$) ppm. UV-vis [CH_2Cl_2, λ_{max} (ε x 10^{-3} M^{-1} cm^{-1})]: 259 (38.90), 276 (34.62), 357 (69.66), 387 (21.31), 445 (33.10) nm.

A4. Generation of **3***

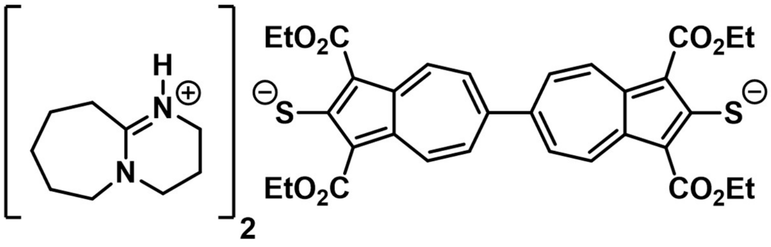

A deep purple solution of **3*** was generated by dissolving **3** (0.020 g, 0.033 mmol) and 1,8-diazabicyclo[5.4.0]undec-7-ene (DBU) (0.027 mL, 0.198 mmol) in 2.3 mL of dry $CDCl_3$ under argon atmosphere at ambient temperature. After 3 hours, an ^{1}H NMR spectrum of the reaction mixture sealed in an NMR tube under argon was collected. ^{1}H NMR (400 MHz, $CDCl_3$): δ 8.30 (d, $^3J_{HH}$ = 11.0 Hz, 4H, $H^{4,4',8,8'}$), 7.39 (d, $^3J_{HH}$ = 11.0 Hz, 4H, $H^{5,5',7,7'}$), 4.55 (q, $^3J_{HH}$ = 7.1 Hz, 8H, CH_2), 1.38 (t, $^3J_{HH}$ = 7.1 Hz, 12H, CH_3) ppm. The ^{1}H NMR spectrum of **3*** also features eight multiplets between 3.40 and 1.50 ppm corresponding to DBU/$DBUH^+$.

The UV-Vis sample of **3*** was prepared by adding excess DBU (0.5 μL, 0.003 mmol) to a 3 mL solution of 2.2 × 10^{-5} M **3** in dry CH_2Cl_2. Measurement was taken immediately, as the product decays fast in the presence of air. UV-vis [CH_2Cl_2, λ_{max} (ε × 10^{-3} M^{-1} cm^{-1})]: 343 (57.01), 414 (13.13), 582 (39.39) nm.

A5. Synthesis of **4**

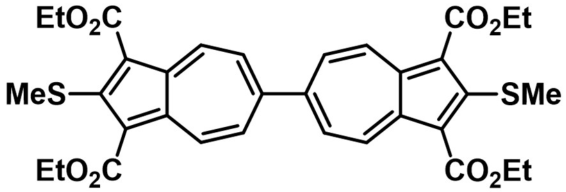

1,8-Diazabicyclo[5.4.0]undec-7-ene (DBU) (0.246 mL, 1.65 mmol) was added to an orange-red solution of **3** (0.100 g, 0.165 mmol) and CH_3I (0.103 mL, 1.65 mmol) in 10 mL of CH_2Cl_2. The mixture acquired an indigo color which then turned dark red within seconds. After stirring at room temperature for 10 minutes, the resulting solution was washed with deionized water (3 × 40 mL) and dried over anhydrous Na_2SO_4. The drying agent was filtered off and the filtrate was concentrated to dryness at 10^{-2} torr to afford a red oil. Triturating this oil with pentane afforded a

dark red solid of **4** (0.104 g, 0.164 mmol) in a 99% yield. Mp: 146–148°C. HRMS (m/z, ES+): Calcd for $C_{34}H_{35}O_8S_2^+$: 635.1773, found 635.1778 $[M+H]^+$. ^{1}H NMR (400 MHz, $CDCl_3$): δ 9.17 (d, $^3J_{HH}$ = 11.2 Hz, 4H, $H^{4,4',8,8'}$), 7.80 (d, $^3J_{HH}$ = 11.2 Hz, 4H, $H^{5,5',7,7'}$), 4.52 (q, $^3J_{HH}$ = 7.1 Hz, 8H, CH_2), 2.62 (s, 6H), 1.49 (t, $^3J_{HH}$ = 7.1 Hz, 12H, CH_3) ppm. $^{13}C\{^1H\}$ NMR (126 MHz, $CDCl_3$): δ 165.58 (C=O), 155.77, 153.28, 141.01, 133.96, 131.13, 118.30 (azulenic C-atoms), 61.16 ($\underline{C}H_2CH_3$), 19.01 ($S\underline{C}H_3$), 14.60 ($CH_2\underline{C}H_3$) ppm. UV-vis [CH_2Cl_2, λ_{max} (ε x 10^{-3} M^{-1} cm^{-1})]: 281 (29.19), 355 (63.49), 461 (37.29) nm.

A6. Synthesis of **5**

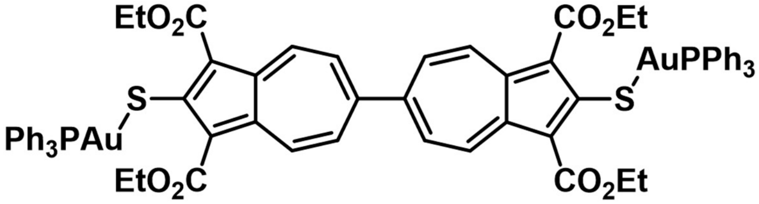

All manipulations described herein were conducted under argon atmosphere with protection from ambient laboratory lighting. A solid mixture of **3** (0.053 g, 0.088 mmol), Ph_3PAuCl (0.095 g, 0.195 mmol), and NaOH (0.035 g, 0.880 mmol) was suspended in dry methanol (10 mL) to afford a purple slurry, which was stirred for 30 min at 25°C. After the addition of dry $CHCl_3$ (10 mL), the mixture was stirred for 24 hours at room temperature. The reaction flask was then opened to air and its content was passed through a 3 cm plug of dry Celite. All solvent was removed under vacuum. The resulting residue was washed with pentane (2 × 15 mL) and then recrystallized from CH_2Cl_2 layered with pentane to afford dark yellow metallic crystals of **5** (0.097 g, 0.064 mmol) in a 73% yield. Mp: 181–183°C. Anal. Calcd for $C_{68}H_{58}Au_2O_8P_2S_2$: C, 53.62; H, 3.84; Found: C, 53.49; H, 3.75. HRMS (m/z, ES+): Calcd for $C_{68}H_{59}Au_2O_8P_2S_2^+$: 1523.2458, found 1523.2458 $[M+H]^+$. ^{1}H NMR (500 MHz, $CDCl_3$): δ 8.82 (d, $^3J_{HH}$ = 11.2 Hz, 4H, $H^{4,4',8,8'}$), 7.64 (d, $^3J_{HH}$ = 11.2 Hz, 4H, $H^{5,5',7,7'}$), 7.44–7.58 (m, 30H, PPh_3), 4.27 (q, $^3J_{HH}$ = 7.2 Hz, 8H, CH_2), 1.35 (t, $^3J_{HH}$ = 7.2 Hz, 12H, CH_3) ppm. $^{13}C\{^1H\}$ NMR (126 MHz, $CDCl_3$): δ 166.84 (C=O), 161.19, 151.64, 140.80 (azulenic C-atoms), 134.42 (d, $^1J_{CP}$ = 13.9 Hz, Ph), 131.80 (d, $^3J_{CP}$ = 2.0 Hz, Ph), 131.76 (Ph), 130.42, 129.85 (azulenic C-atoms), 129.32 (d, $^2J_{CP}$ = 11.6 Hz, Ph), 122.21 (azulenic C-atom), 60.53 ($\underline{C}H_2CH_3$), 14.66 ($CH_2\underline{C}H_3$) ppm. $^{31}P\{^1H\}$ NMR (202 MHz, $CDCl_3$): δ 36.61 ppm. UV-vis [CH_2Cl_2, λ_{max} (ε x 10^{-3} M^{-1} cm^{-1})]: 268 (42.05), 327 (53.58), 357 (79.40), 500 (54.96) nm.

A7. Synthesis of **6**

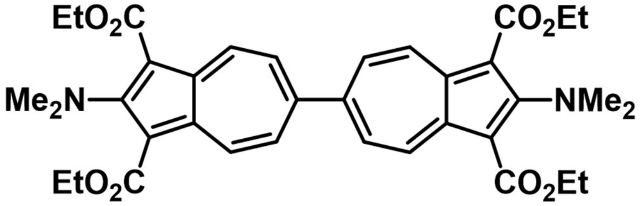

A bright purple solution of **1** (0.148 g, 0.242 mmol) in 200-proof ethanol (20 mL) was treated with 26% w/v aqueous dimethylamine solution (4.20 mL, 24.2 mmol). The reaction mixture was refluxed at 95°C for 4 hours with stirring, gradually acquiring a dark blood red color. The reaction flask content was then quenched with ice-cold deionized H_2O (200 mL). About 1 g of NaCl was added, and the aqueous phase was extracted with CH_2Cl_2 (3 × 100 mL). The combined organic extracts were dried over anhydrous Na_2SO_4. The drying agent was filtered off and the filtrate was concentrated to dryness at 10^{-2} torr to afford oily blood red **6** (0.149 g, 0.237 mmol) in a 98% yield. This oil turned into a dark brown-red solid after redissolution in CH_2Cl_2 and careful layering of the solution with pentane. Mp:104–105°C. HRMS (m/z, ES+): Calcd for $C_{36}H_{41}N_2O_8^+$: 629.2863, found 629.2874 $[M+H]^+$. ^1H NMR (500 MHz, $CDCl_3$): δ 8.68 (d, $^3J_{HH}$ = 11.3 Hz, 4H, $H^{4,4',8,8'}$), 7.64 (d, $^3J_{HH}$ = 11.3 Hz, 4H, $H^{5,5',7,7'}$), 4.47 (q, $^3J_{HH}$ = 7.2 Hz, 8H, CH_2), 3.18 (s, 12H) 1.46 (t, $^3J_{HH}$ = 7.2 Hz, 12H, CH_3) ppm. $^{13}C\{^1H\}$ NMR (126 MHz, $CDCl_3$): δ 166.32 (C=O), 160.76, 148.59, 142.43, 131.45, 128.99, 107.87 (azulenic C-atoms), 60.54 ($\underline{C}H_2CH_3$), 44.56 $\{N(\underline{C}H_3)_2\}$, 14.78 ($CH_2\underline{C}H_3$) ppm. UV-Vis [$CH_2Cl_2$, λ_{max} (ε x 10^{-3} M^{-1} cm^{-1})]: 266 (20.34), 301 (20.29), 350 (41.13), 499 (31.42) nm.

A8. Synthesis of **8**

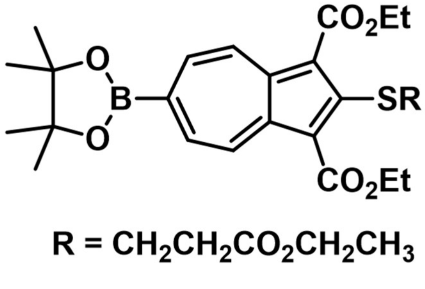

Neat ethyl-3-mercaptopropionate (0.260 mL, 2.05 mmol) was added to a purple solution of **7** (0.571 g, 1.32 mmol) in 35 mL of pyridine. The reaction mixture was refluxed for 3 hours with

stirring. All solvent was then removed under vacuum and the oily residue was re-dissolved in CH_2Cl_2 (20 mL). This solution was washed with deionized H_2O (3 × 30 mL) and then dried over anhydrous Na_2SO_4. The drying agent was filtered off and the filtrate was concentrated to dryness at 10^{-2} torr to afford a dark purple somewhat oily solid of **8** (0.645 g, 1.22 mmol) in a 92% yield. HRMS (m/z, ES+): Calcd for $C_{27}H_{35}BO_8SNa^+$: 553.2043, found 553.2026 $[M+Na]^+$. ^1H NMR (400 MHz, $CDCl_3$): δ 9.11 (d, $^3J_{HH}$ = 10.7 Hz, 2H, $H^{4,8}$), 8.11 (d, $^3J_{HH}$ = 10.7 Hz, 2H, $H^{5,7}$), 4.49 (q, $^3J_{HH}$ = 7.1 Hz, 4H, CH_2), 4.10 (q, $^3J_{HH}$ = 7.1 Hz, 2H, CH_2), 3.33 (t, $^3J_{HH}$ = 7.7 Hz, 2H, CH_2), 2.60 (t, $^3J_{HH}$ = 7.7 Hz, 2H, CH_2), 1.47 (t, $^3J_{HH}$ = 7.1 Hz, 6H, CH_3), 1.39 (s, 12H, CH_3), 1.21 (t, $^3J_{HH}$ = 7.1 Hz, 3H, CH_3) ppm. $^{13}C\{^1H\}$ NMR (126 MHz, $CDCl_3$): δ 171.59, 165.52 (C=O), 152.80, 143.00, 136.20, 134.59, 118.20, 85.13 (azulenic C-atoms), 61.03, 60.84 (CH_2), 34.70, 30.57 (CH_2CH_2S), 25.06, 14.56, 14.27 (CH_3) ppm.

A9. Synthesis of **9**

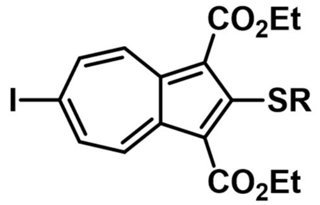

R = $CH_2CH_2CO_2CH_2CH_3$

A colorless slurry of CuI (0.298 g, 1.57 mmol) in 16 mL of DMSO was heated to 90°C and stirred for 5 minutes under air atmosphere, gradually acquiring a dark brown color. Then, **8** (0.166 g, 0.313 mmol) dissolved in 10 mL of DMSO was added to the reaction flask, and the resulting mixture was continued to be stirred at 90°C for 1 hour. After cooling to room temperature, the reaction was quenched with ice-cold deionized H_2O (375 mL) causing precipitation of a dark brown powder. The precipitate was filtered off. The filtercake was washed with deionized H_2O (4 × 40 mL) and then re-dissolved in ethyl acetate (30 mL). The ethyl acetate solution was dried over anhydrous Na_2SO_4. The drying agent was filtered off and the filtrate was concentrated to dryness at 10^{-2} torr to afford a dark red oil. This oil was re-dissolved in boiling heptane and hot-filtered through a plug of cotton. The filtrate was kept at –20°C overnight to afford a scarlet powder of **9** (0.095 g, 0.179 mmol) in a 57% yield. Mp: 87–88°C. HRMS (m/z, ES+): Calcd for

$C_{21}H_{23}IO_6SNa^+$: 553.0158, found 553.0153 $[M+Na]^+$. ^{1}H NMR (400 MHz, $CDCl_3$): δ 8.63 (d, $^3J_{HH}$ = 11.30 Hz, 2H, $H^{4,8}$), 8.14 (d, $^3J_{HH}$ = 11.30 Hz, 2H, $H^{5,7}$), 4.48 (q, $^3J_{HH}$ = 7.16 Hz, 4H, CH_2), 4.10 (q, $^3J_{HH}$ = 7.13 Hz, 2H, CH_2), 3.30 (t, $^3J_{HH}$ = 7.61 Hz, 2H, CH_2), 2.59 (t, $^3J_{HH}$ = 7.61 Hz, 2H, CH_2), 1.46 (t, $^3J_{HH}$ = 7.16 Hz, 6H, CH_3), 1.21 (t, $^3J_{HH}$ = 7.13 Hz, 3H, CH_3) ppm.

A10. Synthesis of **10**

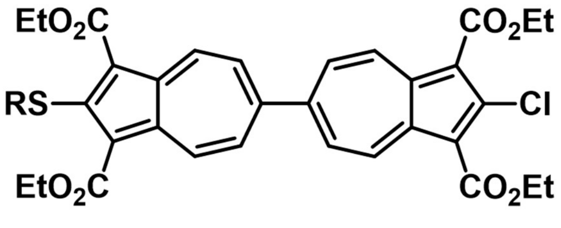

A dark red slurry of **7** (0.130 g, 0.300 mmol), **9** (0.106 g, 0.200 mmol), and Na_2CO_3 (0.042 g, 0.400 mmol) in 24 mL of toluene was treated with 7.5 mL of deionized H_2O. The resulting mixture was thoroughly degassed to remove O_2 and establish inert N_2 atmosphere. $Pd(PPh_3)_4$ (0.0240 g, 0.0208 mmol) was then added as a suspension in 2.5 mL of toluene under N_2 atmosphere. The mixture was refluxed at 115°C for 5 hours, gradually acquiring a dark orange color. The reaction was then quenched with 100 mL of 4:1 H_2O / brine and extracted with 75 mL of $CHCl_3$. The organic layer was separated and the aqueous layer was extracted with 50 mL of $CHCl_3$. The combined organic fractions were washed with deionized H_2O (100 mL) and dried over anhydrous Na_2SO_4. The drying agent was filtered off and the filtrate was concentrated to dryness at 10^{-2} torr to afford a dark red oil. This oil was subjected to column chromatography on silica gel (*ca*. 15 cm x 1.5 cm) using 30:1 CH_2Cl_2 / ethyl acetate eluent. A red-yellow band was collected and concentrated to dryness at 10^{-2} torr to give a somewhat oily dark red solid of **10** (0.102 g, 0.144 mmol) in a 72% yield. HRMS (m/z, ES+): Calcd for $C_{37}H_{37}ClO_{10}SNa^+$: 731.1694, found 731.1714 $[M+Na]^+$. ^{1}H NMR (400 MHz, $CDCl_3$): δ 9.59 (d, $^3J_{HH}$ = 11.2 Hz, 2H, $H^{4',8'}$), 9.23 (d, $^3J_{HH}$ = 11.2 Hz, 4H, $H^{4,8}$), 7.93 (d, $^3J_{HH}$ = 11.2 Hz, 2H, $H^{5',7'}$), 7.82 (d, $^3J_{HH}$ = 11.2 Hz, 2H, $H^{5,7}$), 4.53 (q, $^3J_{HH}$ = 7.1, Hz, 4H, CH_2), 4.52 (q, $^3J_{HH}$ = 7.1, Hz, 4H, CH_2), 4.12 (q, $^3J_{HH}$ = 7.1 Hz, 2H, CH_2), 3.36 (t, $^3J_{HH}$ = 7.4 Hz, 2H, CH_2), 2.63 (t, $^3J_{HH}$ = 7.4 Hz, 2H, CH_2), 1.50 (t, $^3J_{HH}$ = 7.2 Hz, 12H, CH_3), 1.23 (t, $^3J_{HH}$ = 7.1 Hz, 3H, CH_3) ppm.

A11. Synthesis of **11**

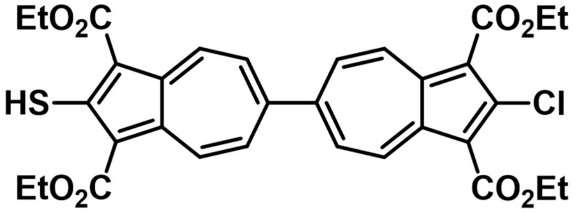

Sodium metal (0.0810 g, 3.52 mmol) was added to a solution of **10** (0.102 g, 0.144 mmol) in 200-proof ethanol (70 mL) with vigorous stirring. Upon consumption of all sodium metal, the resulting purple mixture was warmed to 35°C and stirred for 2 hours. The mixture was then cooled to room temperature and concentrated HCl was added dropwise until acidic pH was established. The resulting mixture was quenched with deionized H_2O (80 mL), and the product was extracted with CH_2Cl_2 (2 × 25 mL). After adding a few dashes of NaCl (s), the combined organic extracts were washed with RO H_2O (90 mL) and the resulting aqueous layer was extracted with CH_2Cl_2 (3 × 25 mL). The combined organic layers were dried over anhydrous Na_2SO_4. The drying agent was filtered off and the filtrate was concentrated to dryness under reduced pressure. The resulting sand-brown powder was washed with pentane and dried under reduced pressure to afford **11** (0.0810 g, 0.133 mmol) in a 92% yield. Mp: 158–159°C. HRMS (m/z, ES–): Calcd for $C_{32}H_{28}ClO_8S^-$: 607.1193, found 607.1164 $[M–H]^-$. ^1H NMR (500 MHz, $CDCl_3$): δ 9.59 (d, $^3J_{HH}$ = 11.0 Hz, 2H, $H^{4',8'}$), 9.53 (d, $^3J_{HH}$ = 11.1 Hz, 2H, $H^{4,8}$), 7.94 (d, $^3J_{HH}$ = 11.0 Hz, 2H, $H^{5',7'}$), 7.89 (d, $^3J_{HH}$ = 11.1 Hz, 2H, $H^{5,7}$), 7.78 (s, 1H), 4.56–4.50 (m, 8H, CH_2), 1.53 (t, $^3J_{HH}$ = 7.1 Hz, 6H, CH_3), 1.50 (t, $^3J_{HH}$ = 7.1 Hz, 6H, CH_3) ppm. $^{13}C\{^1H\}$ NMR (126 MHz, $CDCl_3$): δ 165.87, 164.32 (C=O), 157.46, 155.35, 152.44, 144.51, 143.03, 140.82, 137.25, 134.62, 132.45, 131.87, 116.41, 114.73 (azulenic C-atoms), 61.02, 61.01 ($\underline{C}H_2CH_3$), 14.69, 14.57 ($CH_2\underline{C}H_3$) ppm. UV-Vis [CH_2Cl_2, λ_{max} (ε x 10^{-3} M^{-1} cm^{-1})]: 266 (44.63), 351 (52.79), 365 (45.76), 433 (23.83) nm.

A12. Synthesis of **12**

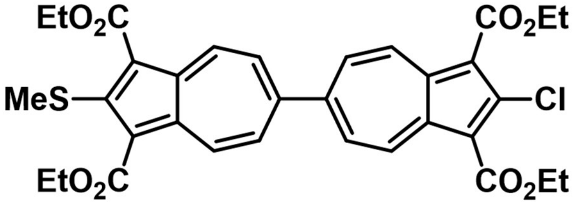

1,8-Diazabicyclo[5.4.0]undec-7-ene (DBU) (0.110 mL, 0.736 mmol) was added to a dark red-orange solution of **11** (0.054 g, 0.089 mmol) and CH_3I (0.046 mL, 0.736 mmol) in 16 mL of $CHCl_3$. The mixture acquired a navy-blue color, which transitioned to dark orange within seconds. After stirring at room temperature for 10 minutes, the resulting solution was diluted with 50 mL of CH_2Cl_2. This solution was washed with deionized water (6 × 80 mL) and dried over anhydrous Na_2SO_4. The drying agent was filtered off and the filtrate was concentrated to dryness at 10^{-2} torr to afford a dark red-orange oil. Triturating this oil with pentane afforded a dark maroon powder of **12** (0.047 g, 0.075 mmol) in an 85% yield. Mp: 138–140°C. HRMS (m/z, ES+): Calcd for $C_{33}H_{31}ClO_8SNa^+$: 645.1326, found 645.1345 $[M+Na]^+$. ^1H NMR (500 MHz, $CDCl_3$): δ 9.58 (d, $^3J_{HH}$ = 11.2 Hz, 2H, $H^{4',8'}$), 9.17 (d, $^3J_{HH}$ = 11.1 Hz, 2H, $H^{4,8}$), 7.93 (d, $^3J_{HH}$ = 11.2 Hz, 2H, $H^{5',7'}$), 7.80 (d, $^3J_{HH}$ = 11.1 Hz, 2H, $H^{5,7}$), 4.52 (q, $^3J_{HH}$ = 7.15 Hz, 8H, CH_2), 1.49 (t, $^3J_{HH}$ = 7.1 Hz, 12H, CH_3) ppm. $^{13}C\{^1H\}$ NMR (126 MHz, $CDCl_3$): δ 165.54, 164.35 (C=O), 156.25, 155.76, 152.57, 144.42, 141.09, 140.81, 137.26, 133.90, 131.90, 131.06, 118.44, 116.38 (azulenic C-atoms), 61.21, 61.02 ($\underline{C}H_2CH_3$), 18.99 ($S\underline{C}H_3$), 14.60, 14.57 ($CH_2\underline{C}H_3$) ppm. UV-Vis [CH_2Cl_2, λ_{max} (ε x 10^{-3} M^{-1} cm^{-1})]: 269 (36.00), 345 (44.40), 368 (34.50), 447 (19.83) nm.

A13. Synthesis of **14**

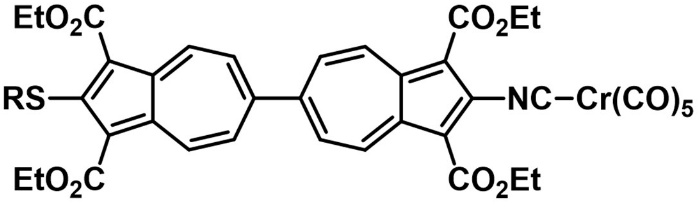

R = CH$_2$CH$_2$CO$_2$CH$_2$CH$_3$

A slurry of **13** (0.1056 g, 0.1858 mmol), **8** (0.0986 g, 0.1859 mmol), $Pd(PPh_3)_4$ (0.0066 g, 0.0057 mmol), and $NaHCO_3$ (0.098 g, 1.17 mmol) in 9.0 mL of toluene and 7.0 mL of 70% v/v

EtOH/H_2O was refluxed for 3 hours gradually acquiring a maroon color. The reaction mixture was then poured into 30 mL of deionized H_2O and extracted with 3 × 20 mL of CH_2Cl_2. The organic extracts were combined, washed with 3 × 50 mL of H_2O, and dried over anhydrous Na_2SO_4. The drying agent was filtered off and the filtrate was concentrated to dryness at 10^{-2} torr. The residue was subjected to column chromatography on silica gel (*ca.* 10 cm x 1 cm) using 4:1 hexanes / ethyl acetate eluent. A brown-orange band was collected. After solvent removal under reduced pressure, the deep brown oily residue was re-dissolved in CH_2Cl_2 and the solution was layered with pentane to crash out microcrystalline **14** (0.1050 g, 0.1177 mmol) in a 63% yield. Mp: 84-87°C. Anal. Calcd for $C_{43}H_{37}CrNO_{15}S$: C, 57.91; H, 4.13; N, 1.57, Found: C, 57.41; H, 4.18; N, 1.57. IR (CH_2Cl_2): $\nu_{N\equiv C}$ 2138, $\nu_{C\equiv O}$ 2046 ($A_1^{(1)}$), $\nu_{C\equiv O}$ 1960 ($A_1^{(2)}$ + E), $\nu_{C=O}$ 1731, 1693 (ester) cm^{-1}. HRMS (m/z, ES+): Calcd for $C_{43}H_{38}CrNO_{15}S^+$: 892.1367, found 892.1387 $[M+H]^+$. ^1H NMR (400 MHz, $CDCl_3$): δ 9.91 (d, $^3J_{HH}$ = 11.3 Hz, 2H, $H^{4,8}$), 9.27 (d, $^3J_{HH}$ = 11.2 Hz, 2H, $H^{4',8'}$), 8.05 (d, $^3J_{HH}$ = 11.3 Hz, 2H, $H^{5,7}$), 7.85 (d, $^3J_{HH}$ = 11.2 Hz, 2H, $H^{5',7'}$), 4.62 (q, $^3J_{HH}$ = 7.2 Hz, 4H, CH_2), 4.55 (q, $^3J_{HH}$ = 7.2 Hz, 4H, CH_2), 4.15 (q, $^3J_{HH}$ = 7.1 Hz, 2H, CH_2), 3.39 (t, $^3J_{HH}$ = 7.6 Hz, 2H, CH_2), 2.66 (t, $^3J_{HH}$ = 7.6 Hz, 2H, CH_2), 1.54 (t, $^3J_{HH}$ = 7.2 Hz, 6H, CH_3), 1.52 (t, $^3J_{HH}$ = 7.2 Hz, 6H, CH_3), 1.26 (t, $^3J_{HH}$ = 7.1 Hz, 3H, CH_3) ppm. $^{13}C\{^1H\}$ NMR (126 MHz, $CDCl_3$): δ 216.59 (CO_{trans}), 214.52 (CO_{cis}), 184.32 (NC), 171.48, 165.32, 163.39 (C=O), 157.20, 153.19, 152.68, 141.12, 141.03, 139.60, 134.70, 133.00, 132.29, 131.02, 119.53, 113.31 (azulenic C-atoms), 61.35, 61.22, 60.95 (CH_2), 34.61, 30.66 (CH_2CH_2S), 14.85, 14.58, 14.30 (CH_3) ppm. UV-Vis [CH_2Cl_2, λ_{max} ($\varepsilon \times 10^{-3}$ M^{-1} cm^{-1})]: 329 (60.6), 358 (66.4), 482 (42.4) nm.

A14. Synthesis of **15**

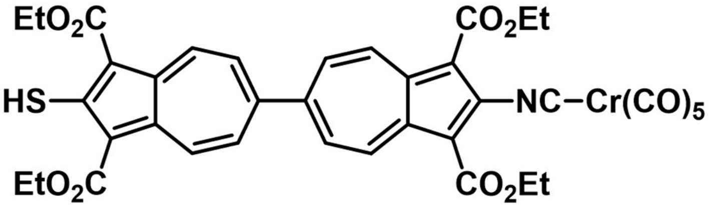

Deionized H_2O and 3M aqueous H_2SO_4 were both purged with argon for 1 hour prior to use in the following procedure. A solution of sodium ethoxide prepared by carefully dissolving sodium metal (0.0225g, 0.9790 mmol) in 10 mL of EtOH was transferred into a flask containing a

suspension of **14** (0.0616 g, 0.0691 mmol) in 50 mL EtOH. The reaction mixture was stirred for 2 hours at ambient temperature. During this period, the mixture slowly acquired a blue hue. Then the mixture was diluted with 250 mL of deionized H_2O and slowly acidified with 3M aqueous H_2SO_4 until a precipitate formed. At this point, the reaction flask was opened to air. The precipitate was filtered off and washed with H_2O. The brown-red solid was redissolved in a minimum amount of CH_2Cl_2 and the solution was passed through a 2 cm silica gel plug using neat CH_2Cl_2 eluent. The resulting orange solution was concentrated to dryness to give a residue which was then crystallized from CH_2Cl_2 layered with pentane. Upon solvent diffusion, brown microcrystals formed. These crystals were filtered off and dried at 10^{-2} torr to afford **15** (0.0392 g, 0.0495 mmol) in a 72% yield. Mp: 158°C (dec.) HRMS (m/z, ES-): Calcd for $C_{35}H_{28}CrNO_{13}S^-$: 790.0692, found 790.0700 $[M–H]^-$. IR (CH_2Cl_2): ν_{SH} 2586, $\nu_{N≡C}$ 2137, $\nu_{C≡O}$ 2046 ($A_1^{(1)}$), 1960 ($A_1^{(2)}$ + E), $\nu_{C=O}$ 1690 (ester) cm^{-1}. ^1H NMR (400 MHz, $CDCl_3$): δ 9.89 (d, $^3J_{HH}$= 11.3 Hz, 2H, $H^{4,8}$), 9.55 (d, $^3J_{HH}$ = 11.4 Hz, 2H, $H^{4',8'}$), 8.04 (d, $^3J_{HH}$ = 11.3 Hz, 2H, $H^{5,7}$), 7.90 (d, $^3J_{HH}$ = 11.4 Hz, 2H, $H^{5',7'}$), 7.80 (s, 1H, SH), 4.60 (q, $^3J_{HH}$= 7.1 Hz, 4H, CH_2), 4.55 (q, $^3J_{HH}$ =7.1 Hz, 4H, CH_2), 1.54 (t, $^3J_{HH}$ = 7.1 Hz, 6H, CH_3), 1.52 (t, $^3J_{HH}$ = 7.1 Hz, 6H, CH_3) ppm. $^{13}C\{^1H\}$ NMR (126 MHz, $CDCl_3$): δ 216.58 (CO_{trans}), 214.53 (CO_{cis}), 184.34 (NC), 165.85, 163.40 (C=O), 157.85, 156.94, 151.86, 143.08, 141.11, 139.58, 134.59, 132.99, 132.40, 132.29, 114.84, 113.31 (azulenic C-atoms), 61.23, 61.08 ($\underline{C}H_2CH_3$), 14.85, 14.68 ($CH_2\underline{C}H_3$) ppm. UV-Vis [CH_2Cl_2, λ_{max} ($\varepsilon \times 10^{-3}$ M^{-1} cm^{-1})]: 324 (51.6), 361 (68.9), 479 (38.9) nm.

A15. Generation of **15***

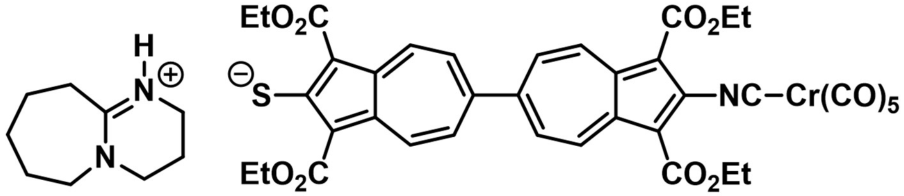

In a glove box filled with argon atmosphere, a navy-blue solution of **15*** was prepared in an NMR tube attached to a widget by adding a slight excess of DBU (2.6 μL, 0.017 mmol) to **15** (0.013 g, 0.016 mmol) dissolved in *ca.* 0.8 mL $CDCl_3$. The NMR tube was sealed off under argon. ^1H NMR (500 MHz, $CDCl_3$): δ 9.76 (d, $^3J_{HH}$ = 10.9 Hz, 2H, $H^{4,8}$), 8.38 (d, $^3J_{HH}$ = 10.8 Hz, 2H,

$H^{4',8'}$), 8.04 (d, $^3J_{HH}$ = 10.9 Hz, 2H, $H^{5,7}$), 7.44 (d, $^3J_{HH}$ = 10.8 Hz, 2H, $H^{5',7'}$), 4.57 (q, $^3J_{HH}$ = 7.1 Hz, 4H, CH_2), 4.45 (q, $^3J_{HH}$ = 7.3 Hz, 4H, CH_2), 1.50 (t, $^3J_{HH}$ = 7.1 Hz, 12H, CH_3), 1.44 (t, $^3J_{HH}$ = 7.0 Hz, 6H, CH_3) ppm. The ^1H NMR spectrum of **15*** also features multiplets between 3.40 and 1.50 ppm corresponding to DBU/$DBUH^+$.

$^{13}C\{^1H\}$ NMR (126 MHz, $CDCl_3$): δ 216.89 (CO_{trans}), 214.69 (CO_{cis}), 183.18 (azulenic C-atom), 182.40 (NC), 176.10 (C7), 167.61, 163.62 (C=O), 162.50 (C7*), 143.31, 142.39, 140.41, 139.35, 133.14, 131.02, 130.22, 128.94, 125.50, 123.19, 112.66 (azulenic C-atoms), 60.96, 60.03 ($\underline{C}H_2CH_3$), 53.24 (C2*), 49.56 (C2), 48.56 (C11*), 45.30 (C11), 43.31 (C9*), 39.11(C9), 37.42 (C6), 36.65 (C6*), 31.77 (C3), 30.16 (C5), 29.79 (C3*), 28.78 (C4), 28.40 (C5*), 25.80 (C4*), 23.64 (C10), 22.15 (C10*), 14.86 ($CH_2\underline{C}H_3$) ppm. The ^{13}C NMR chemical shifts of the resonances corresponding to excess DBU are indicated by an asterisk (*).

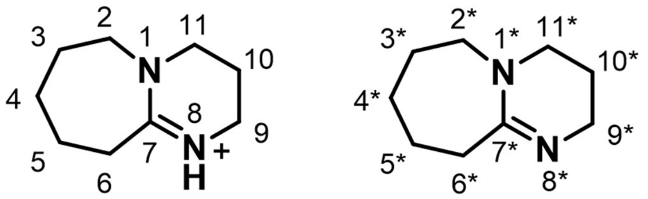

The UV-vis sample was prepared by adding excess DBU (2.6 μL, 0.017 mmol) to a 25 mL solution of 8.6 × 10^{-6} M **15** in dry CH_2Cl_2. Measurement was taken immediately, as the product decays fast in the presence of air. UV-vis [CH_2Cl_2, λ_{max} ($\varepsilon \times 10^{-3}$ M^{-1} cm^{-1})]: 335 (57.6), 455 (22.1), 661 (30.6) nm.

A16. Synthesis of **16**

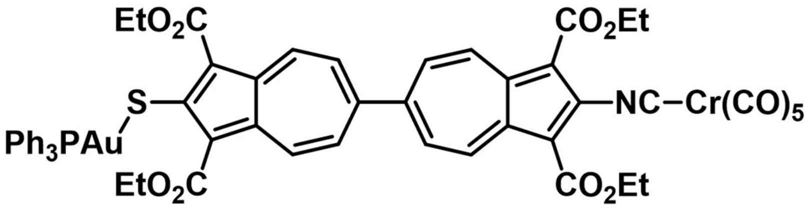

All manipulations in the following procedure were conducted with protection from ambient laboratory lighting. A solid mixture of Me_2SAuCl (0.0052 g, 0.0180 mmol) and PPh_3 (0.0046 g, 0.0180 mmol) was dissolved in 20 mL of dry CH_2Cl_2 and the resulting solution was stirred for 1 hour at room temperature. The solvent was removed under vacuum. The residue was washed with

5 mL of pentane and then redissolved in 10 mL of CH_2Cl_2. This solution was cannulated into a flask containing blue slurry formed from **15** (0.0137 g, 0.0173 mmol) and sodium ethoxide, which was prepared by dissolving sodium metal (0.0076 g, 0.34 mmol) in 10 mL of dry ethanol. Then, all solvent was removed under vacuum and the residue was redissolved in minimum CH_2Cl_2. This solution was sequentially passed through a short plug of Celite and a basic alumina column (*ca*. 8 cm x 1cm) using 10:1 CH_2Cl_2 / ethyl acetate eluent. A bright pink band was collected, and the solvent was removed under reduced pressure. The residue was crystallized by carefully layering pentane over its solution in CH_2Cl_2 to afford deep purple crystals of **16** (0.0164g, 0.0131mmol) in a 76% yield. Mp: 133-135°C. Anal. Calcd for $C_{56}H_{43}AuCrNO_{13}PS$: C, 53.81; H, 3.47; N, 1.12. Found: C, 53.70; H, 3.46; N, 1.11. IR (CH_2Cl_2): $\nu_{N\equiv C}$ 2138, $\nu_{C\equiv O}$ 2047 $A_1^{(1)}$, 1959 ($A_1^{(2)}$ + E), $\nu_{C=O}$ 1693 (ester) cm^{-1}. ^1H NMR (400 MHz, $CDCl_3$): δ 9.85 (d, $^3J_{HH}$ = 11.1 Hz, 2H, $H^{4,8}$), 8.84 (d, $^3J_{HH}$ = 11.1 Hz, 2H, $H^{4',8'}$), 8.03 (d, $^3J_{HH}$ = 11.1 Hz, 2H, $H^{5,7}$), 7.64 (d, $^3J_{HH}$ = 11.1 Hz, 2H, $H^{5',7'}$), 7.45–7.60 (m, 15H, PPh_3), 4.59 (q, $^3J_{HH}$ = 7.1 Hz, 4H, CH_2), 4.27 (q, $^3J_{HH}$ = 7.1 Hz, 4H, CH2), 1.51 (t, $^3J_{HH}$ = 7.1 Hz, 6H, CH_3), 1.36 (t, $^3J_{HH}$ = 7.1 Hz, 6H, CH_3) ppm. $^{13}C\{^1H\}$ NMR (126 MHz, $CDCl_3$): δ 216.72 (CO_{trans}), 214.58 (CO_{cis}), 183.53 (NC), 166.71, 163.48 (C=O), 163.33, 158.41, 149.32, 140.98, 140.89, 139.56 (azulenic C-atoms), 134.39 (d, $^2J_{CP}$ = 13.8 Hz, Ph), 133.24 (azulenic C-atom), 132.32 (Ph), 131.89, 131.43, 130.22 (azulenic C-atoms), 129.44 (d, $^3J_{CP}$ = 56.7 Hz, Ph), 129.36 (d, $^1J_{CP}$ = 11.6 Hz, Ph), 122.70, 113.02 (azulenic C-atoms), 61.13, 60.72 ($\underline{C}H_2CH_3$), 14.86, 14.62 ($CH_2\underline{C}H_3$) ppm. $^{31}P\{^1H\}$ NMR (162 MHz, $CDCl_3$): 36.63 ppm. UV-Vis [CH_2Cl_2, λ_{max} ($\varepsilon \times 10^{-3}$ M^{-1} cm^{-1})]: 338 (61.4), 369 (42.0), 517 (39.8) nm.

B. NMR Data

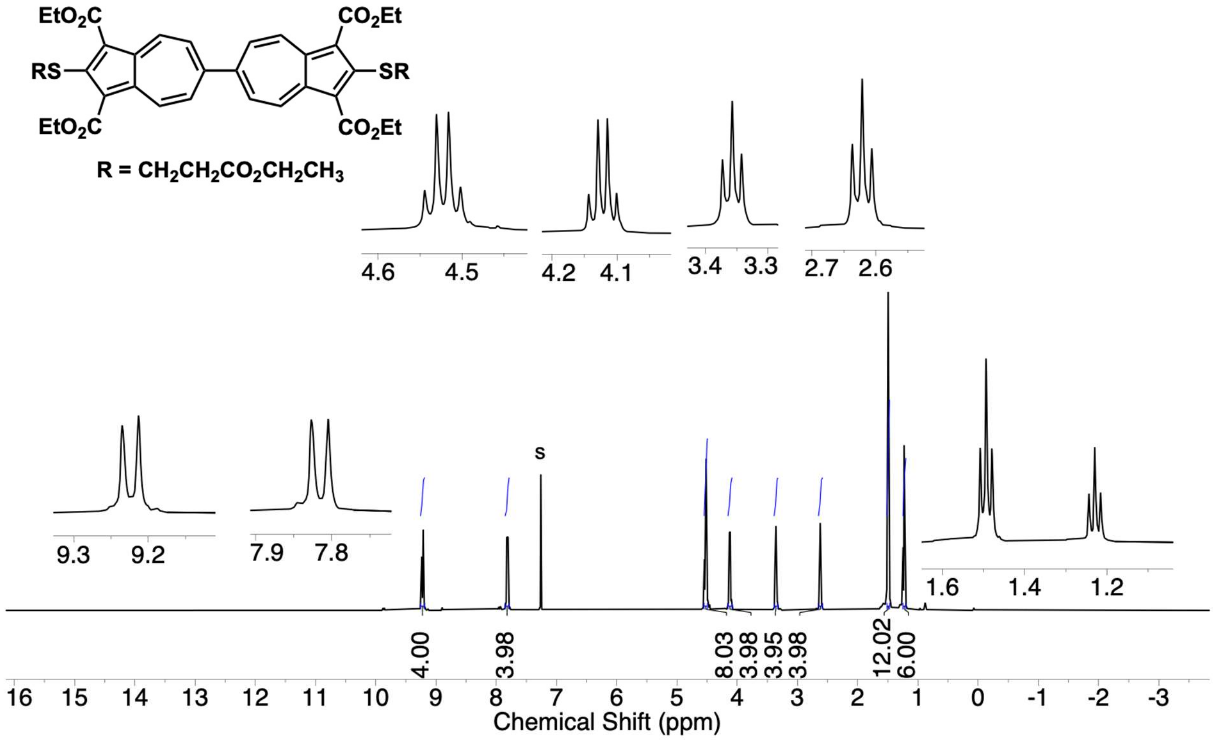

Figure S1. ^{1}H HMR (500 MHz, $CDCl_3$, 25°C) of **2**. S = $CHCl_3$ solvent residual.

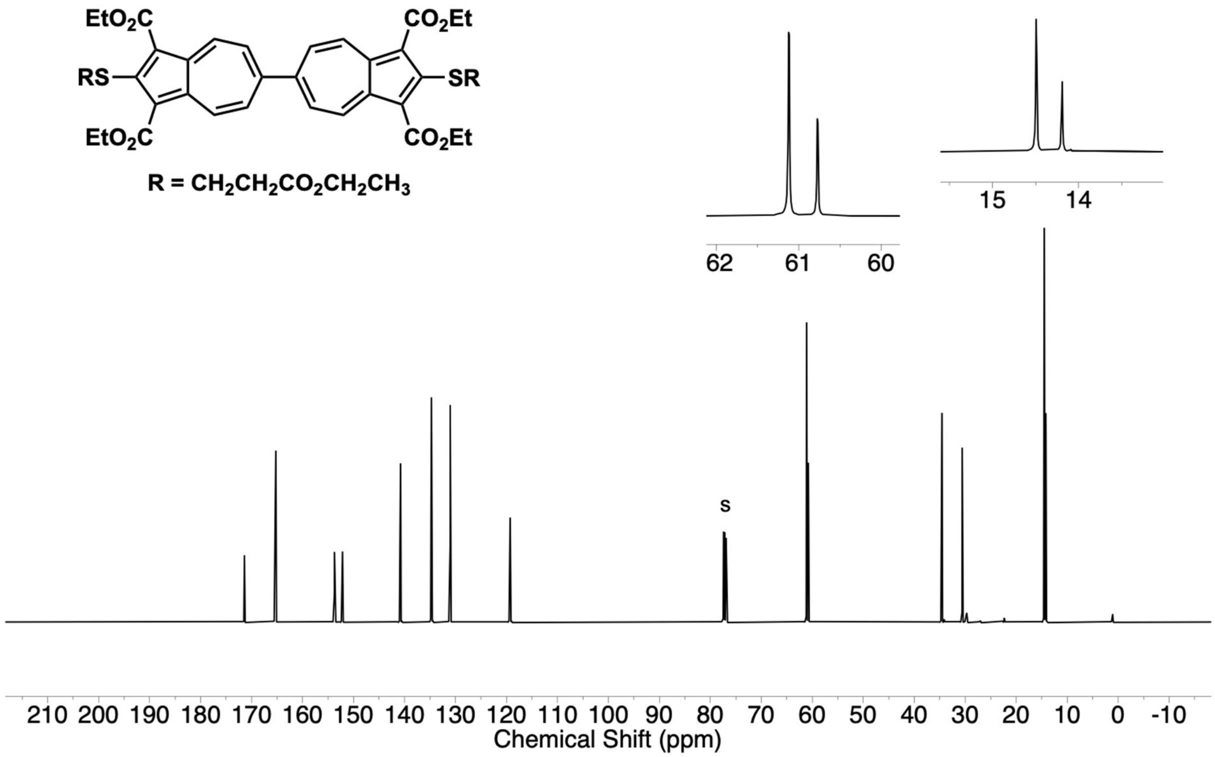

Figure S2. ^{13}C HMR (126 MHz, $CDCl_3$, 25°C) of **2**. S = $CHCl_3$ solvent residual.

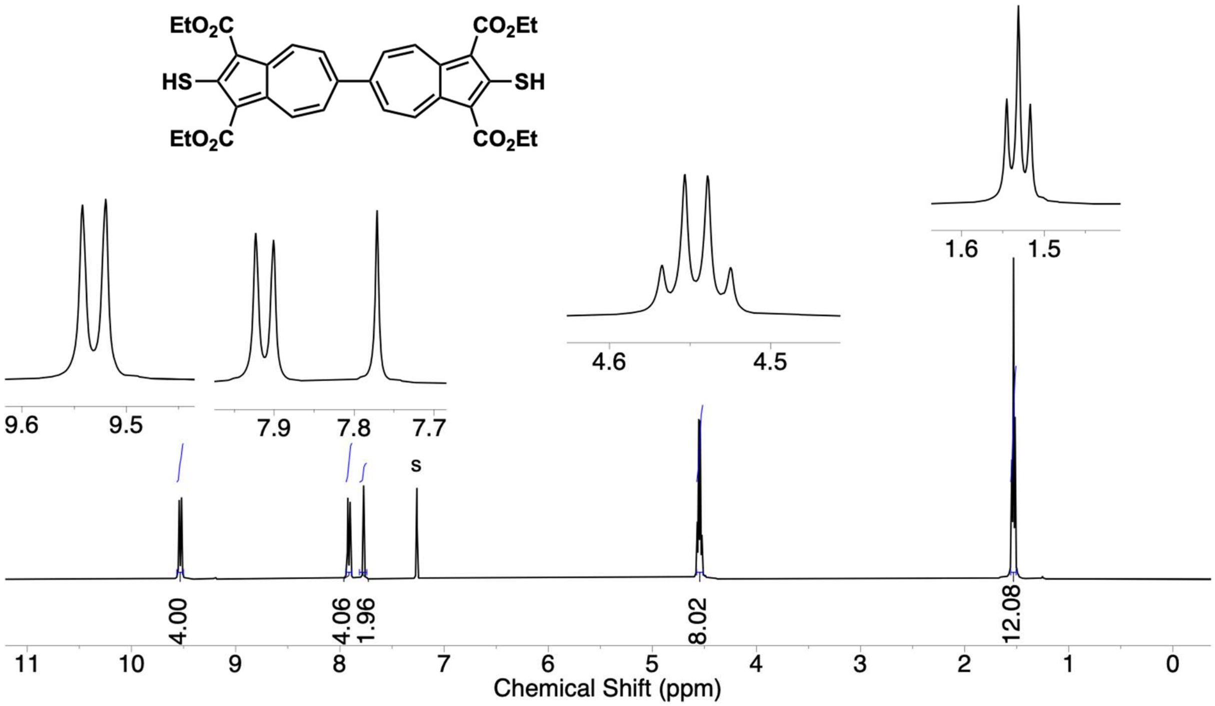

Figure S3. ^{1}H HMR (500 MHz, $CDCl_3$, 25°C) of **3**. S = $CHCl_3$ solvent residual.

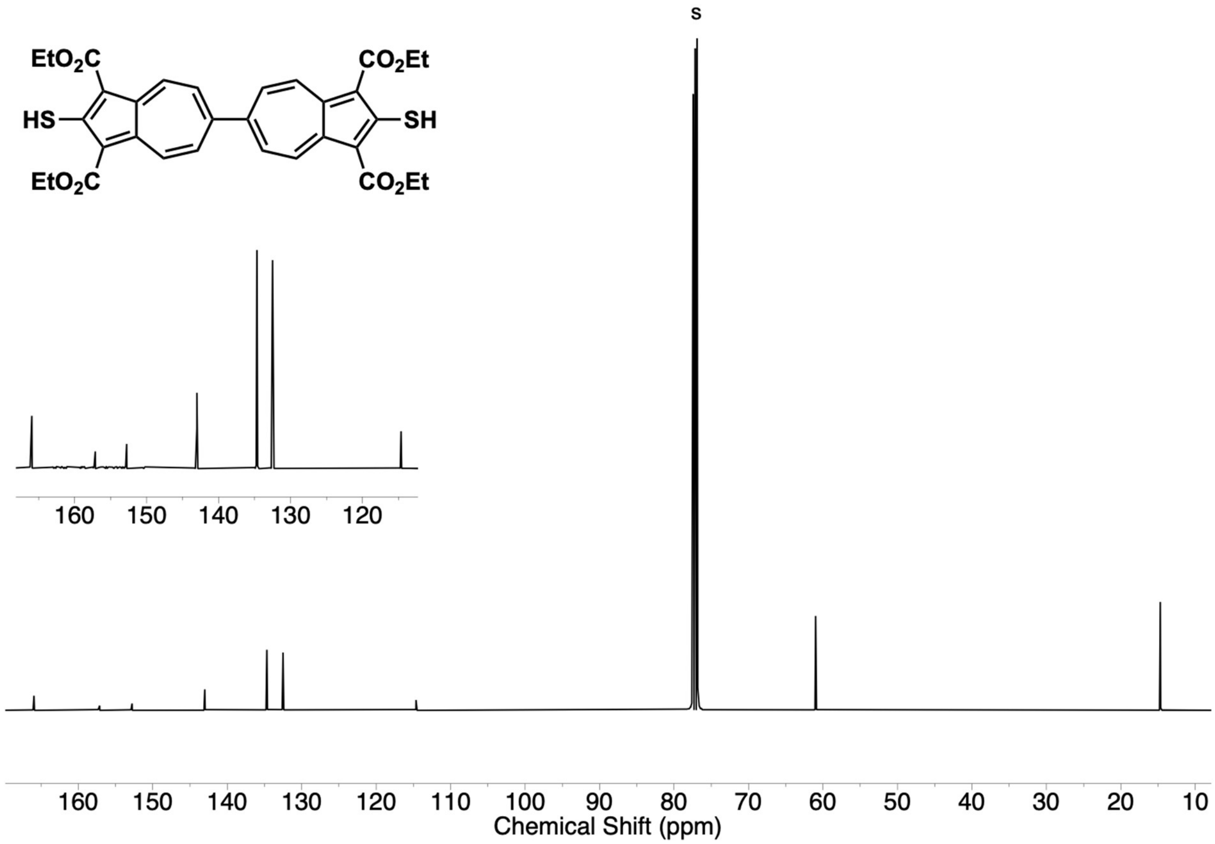

Figure S4. ^{13}C HMR (126 MHz, $CDCl_3$, 25°C) of **3**. S = $CHCl_3$ solvent residual.

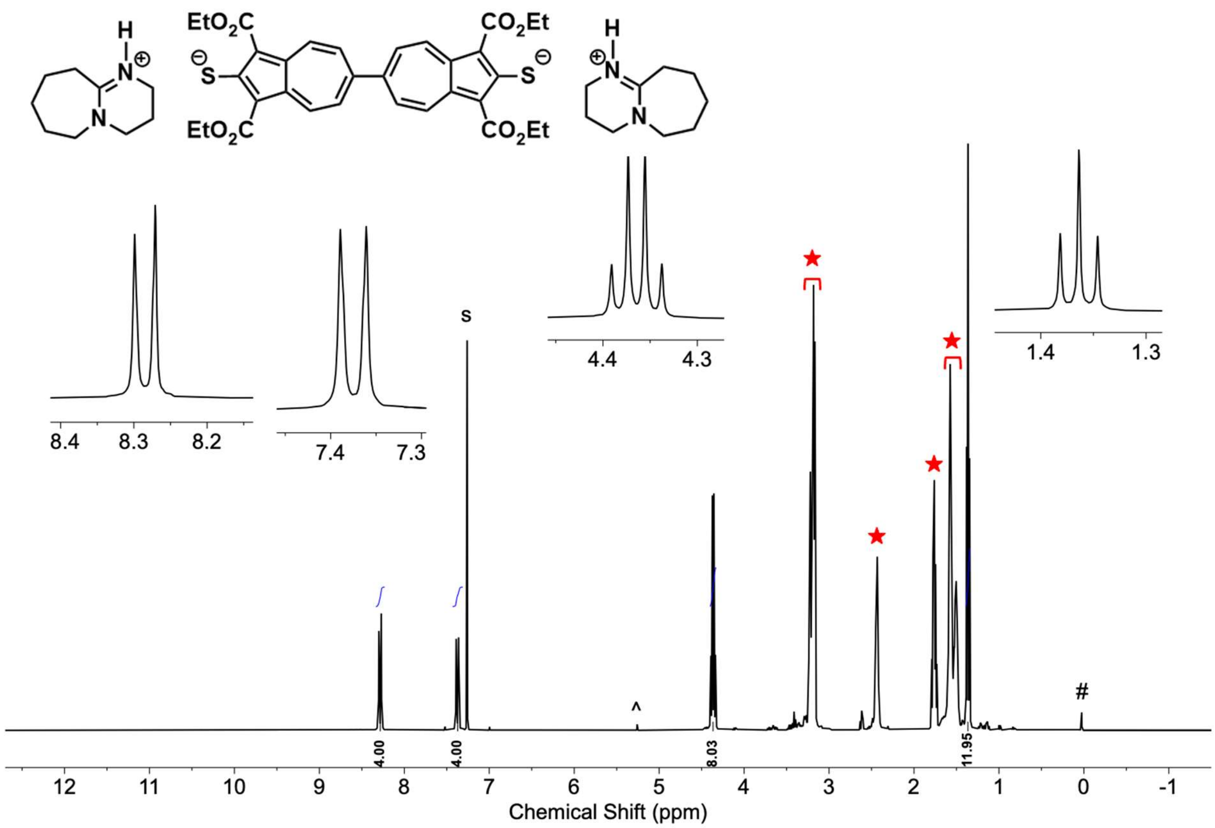

Figure S5. ^{1}H HMR (400 MHz, $CDCl_3$, 25°C) of **3***. (★) denotes $DBUH^+$/DBU resonances resulting from rapid $DBUH^+ \rightleftharpoons$ DBU exchange. S = $CHCl_3$ solvent residual; ^CH_2Cl_2 impurity; #silicone grease.

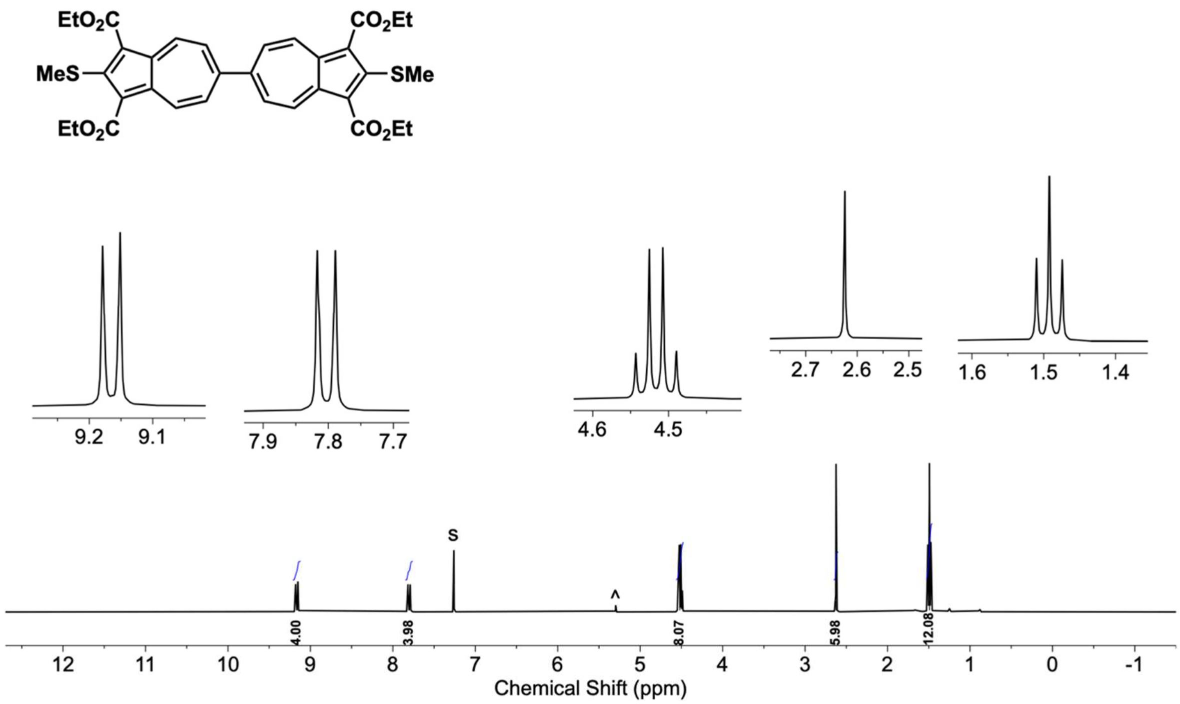

Figure S6. ^{1}H HMR (400 MHz, $CDCl_3$, 25°C) of **4**. S = $CHCl_3$ solvent residual; ^CH_2Cl_2 impurity.

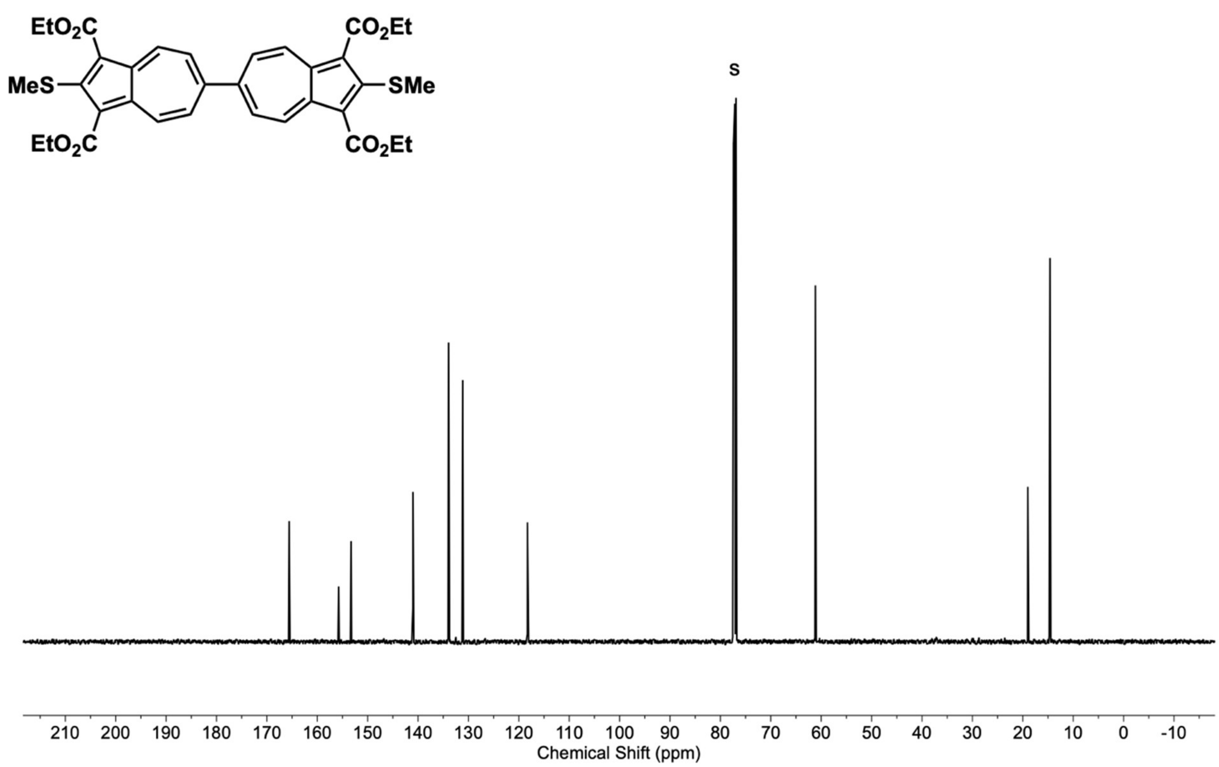

Figure S7. ^{13}C HMR (126 MHz, $CDCl_3$, 25°C) of **4**. S = $CHCl_3$ solvent residual.

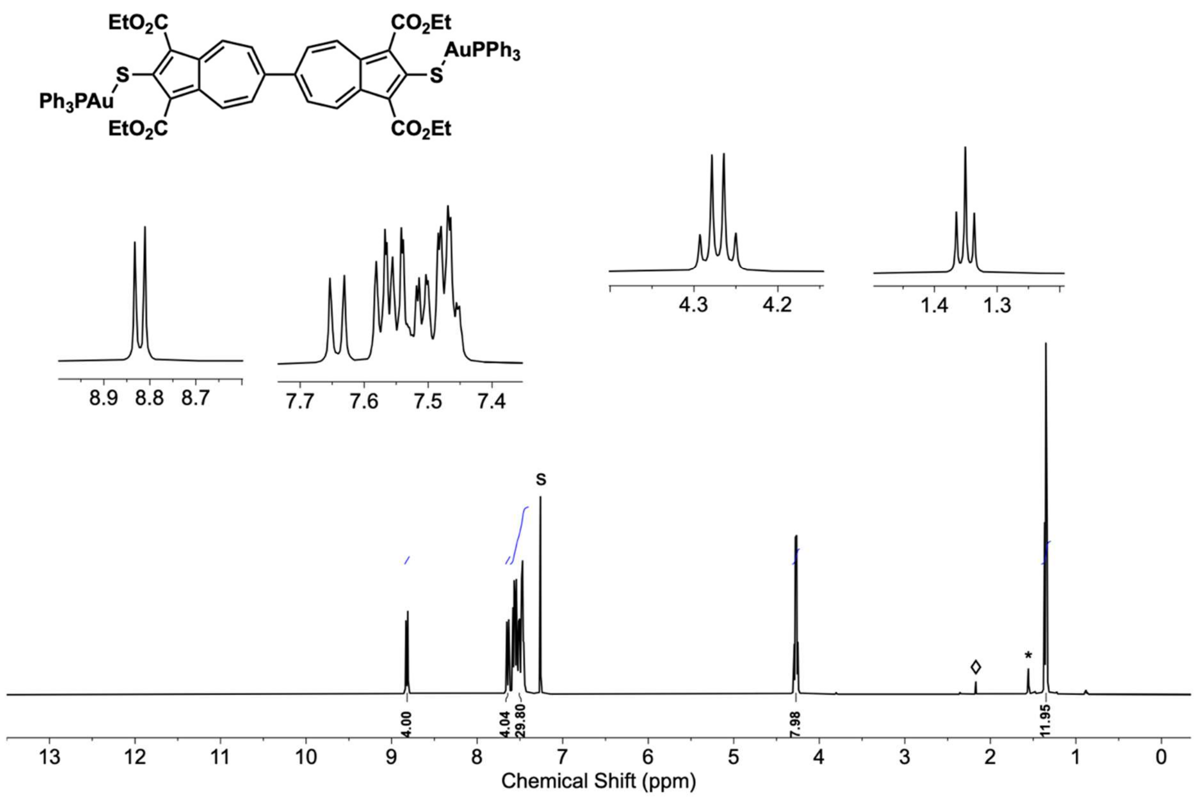

Figure S8. ^{1}H HMR (500 MHz, $CDCl_3$, 25°C) of **5**. S = $CHCl_3$ solvent residual; $^{\lozenge}$acetone impurity; $^{*}H_2O$ impurity in solvent.

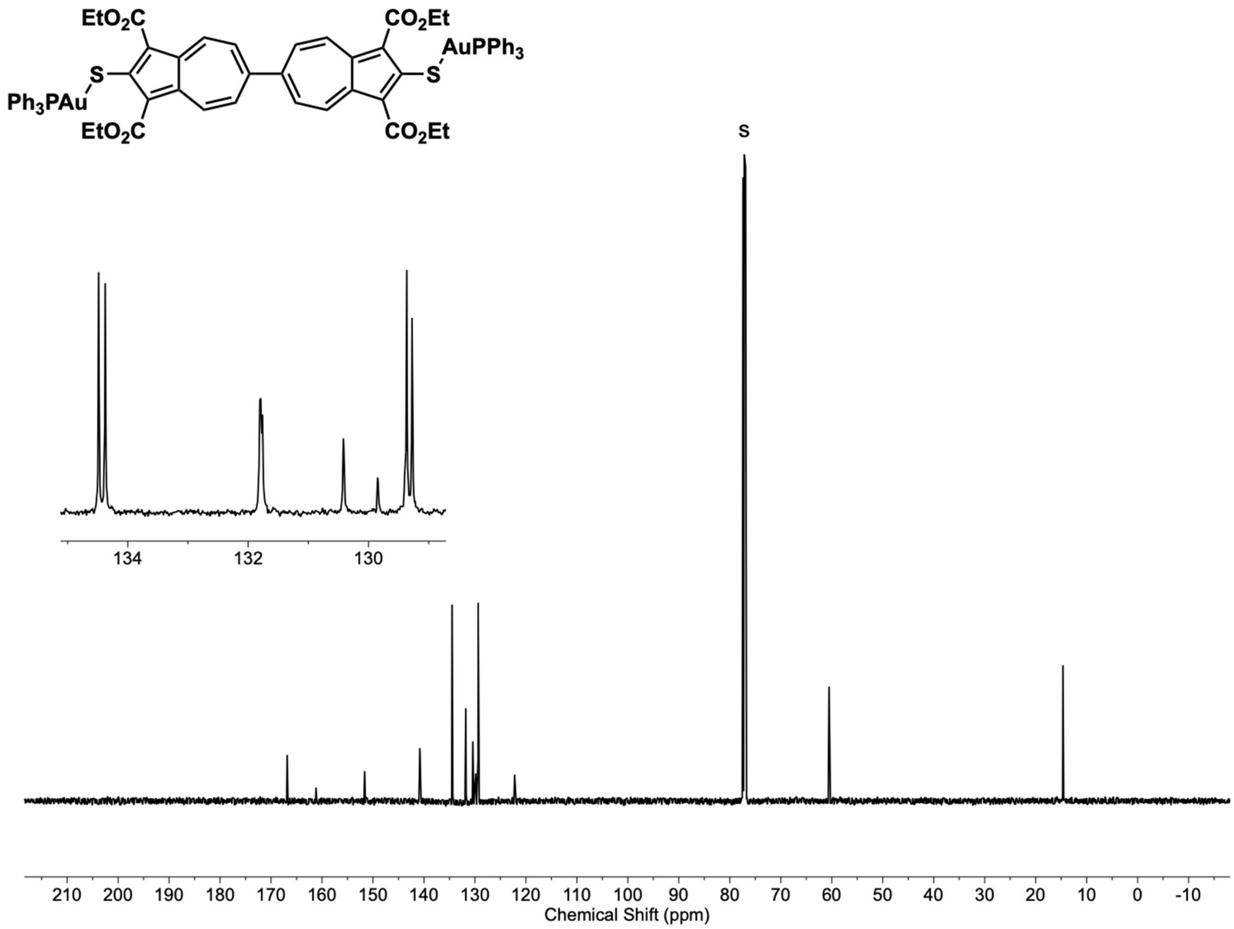

Figure S9. ^{13}C HMR (126 MHz, $CDCl_3$, 25°C) of **5**. S = $CHCl_3$ solvent residual.

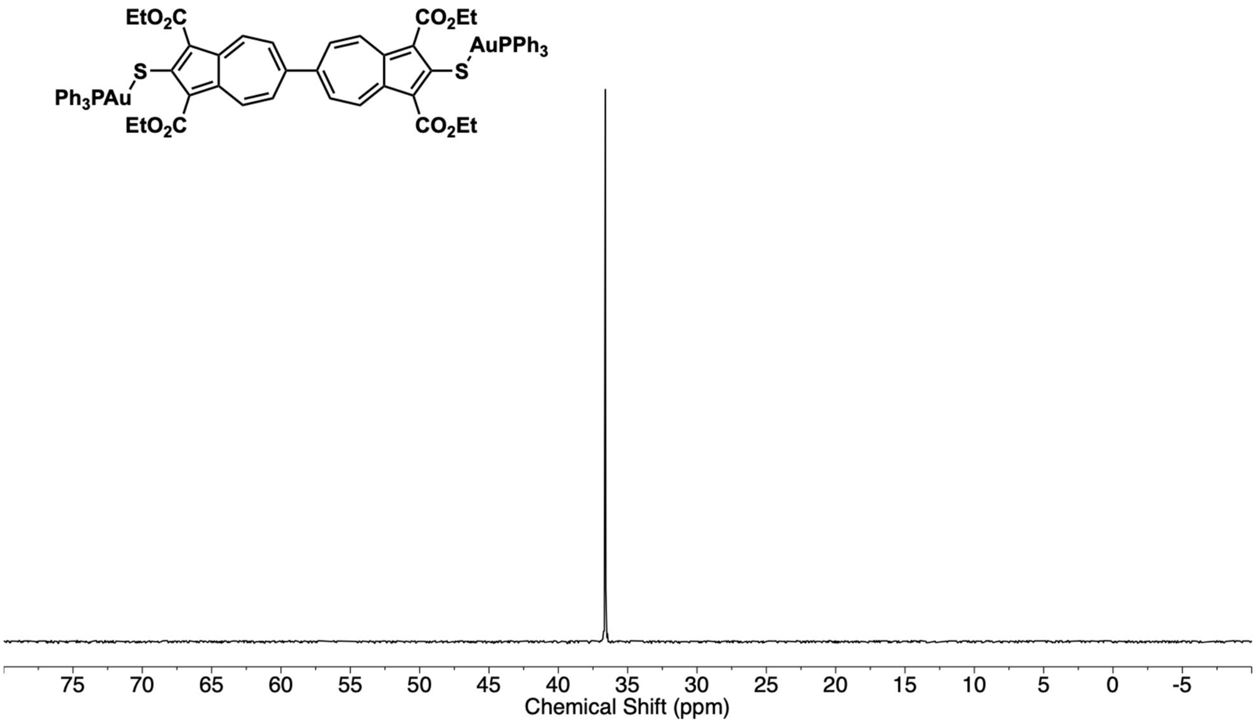

Figure S10. $^{31}P\{^{1}H\}$ NMR (202 MHz, $CDCl_3$) spectrum of **5**.

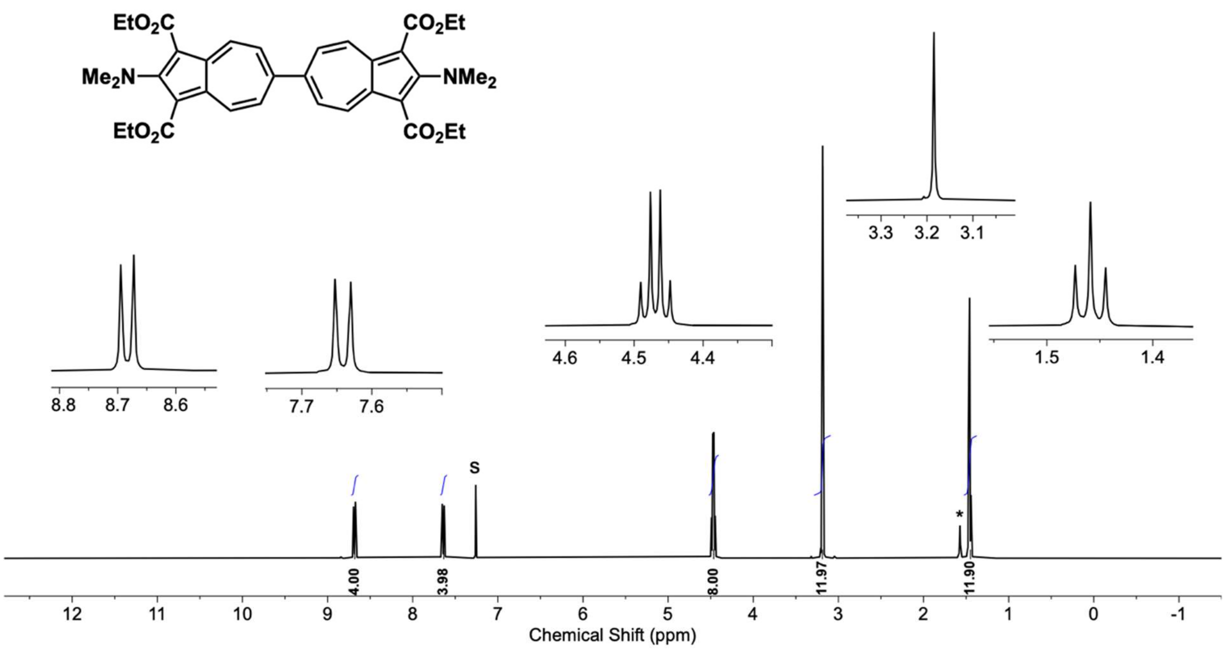

Figure S11. ^{1}H HMR (500 MHz, $CDCl_3$, 25°C) of **6**. S = $CHCl_3$ solvent residual; $^*H_2O$ impurity in solvent.

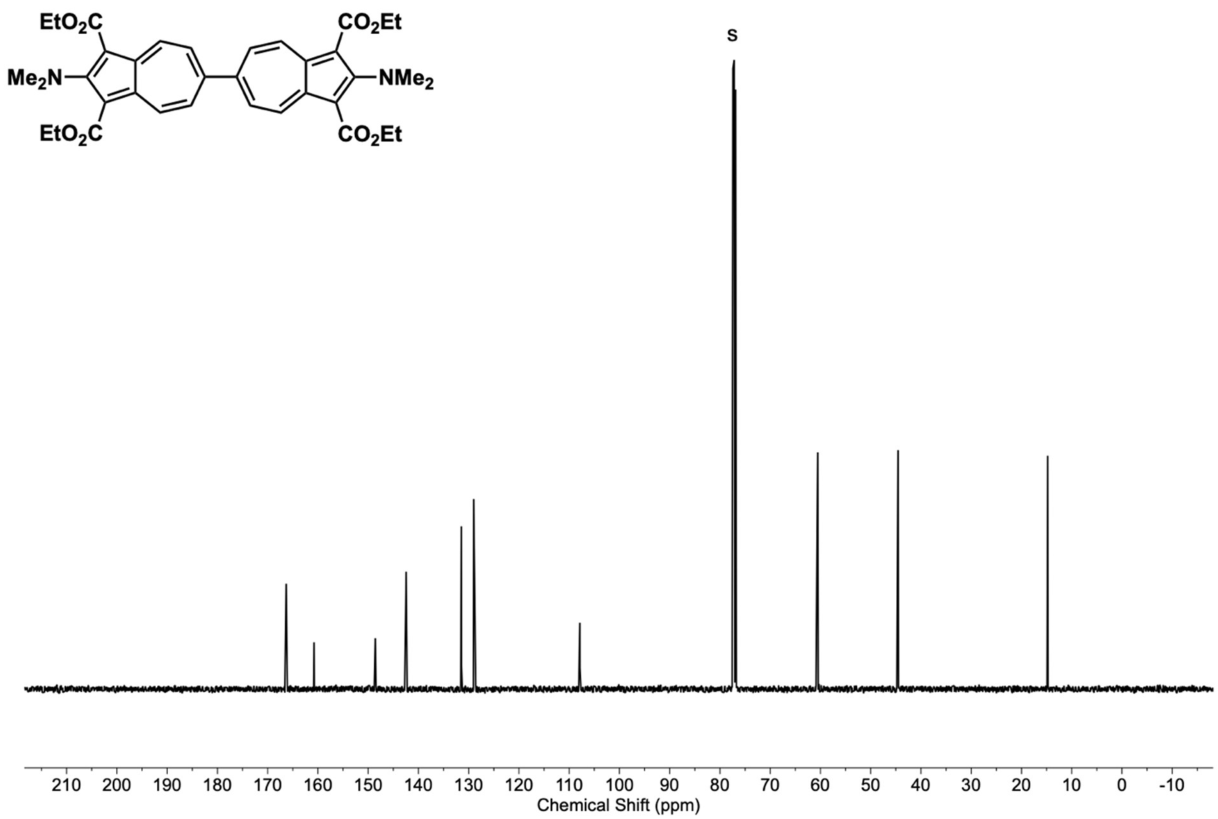

Figure S12. ^{13}C HMR (126 MHz, $CDCl_3$, 25°C) of **6**. S = $CHCl_3$ solvent residual.

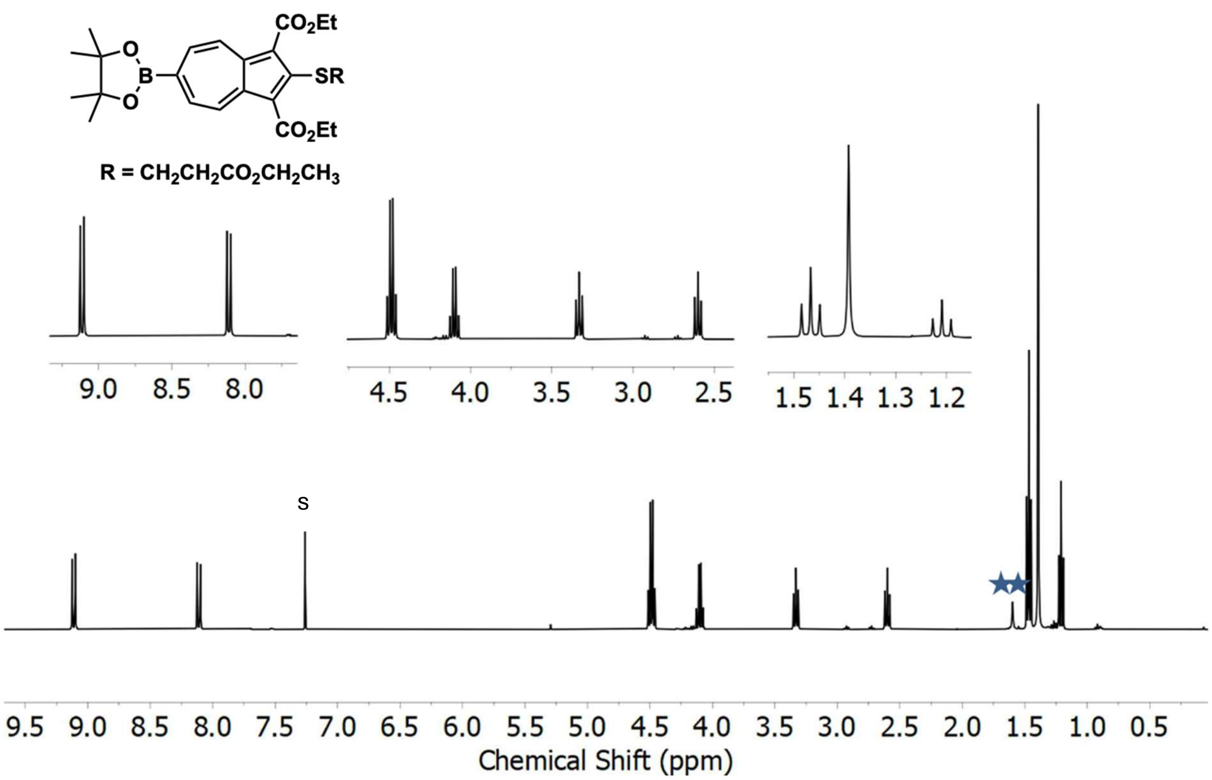

Figure S13. ^{1}H HMR (500 MHz, $CDCl_3$, 25°C) of **8**. S = $CHCl_3$ solvent residual; ★★H_2O impurity in solvent.

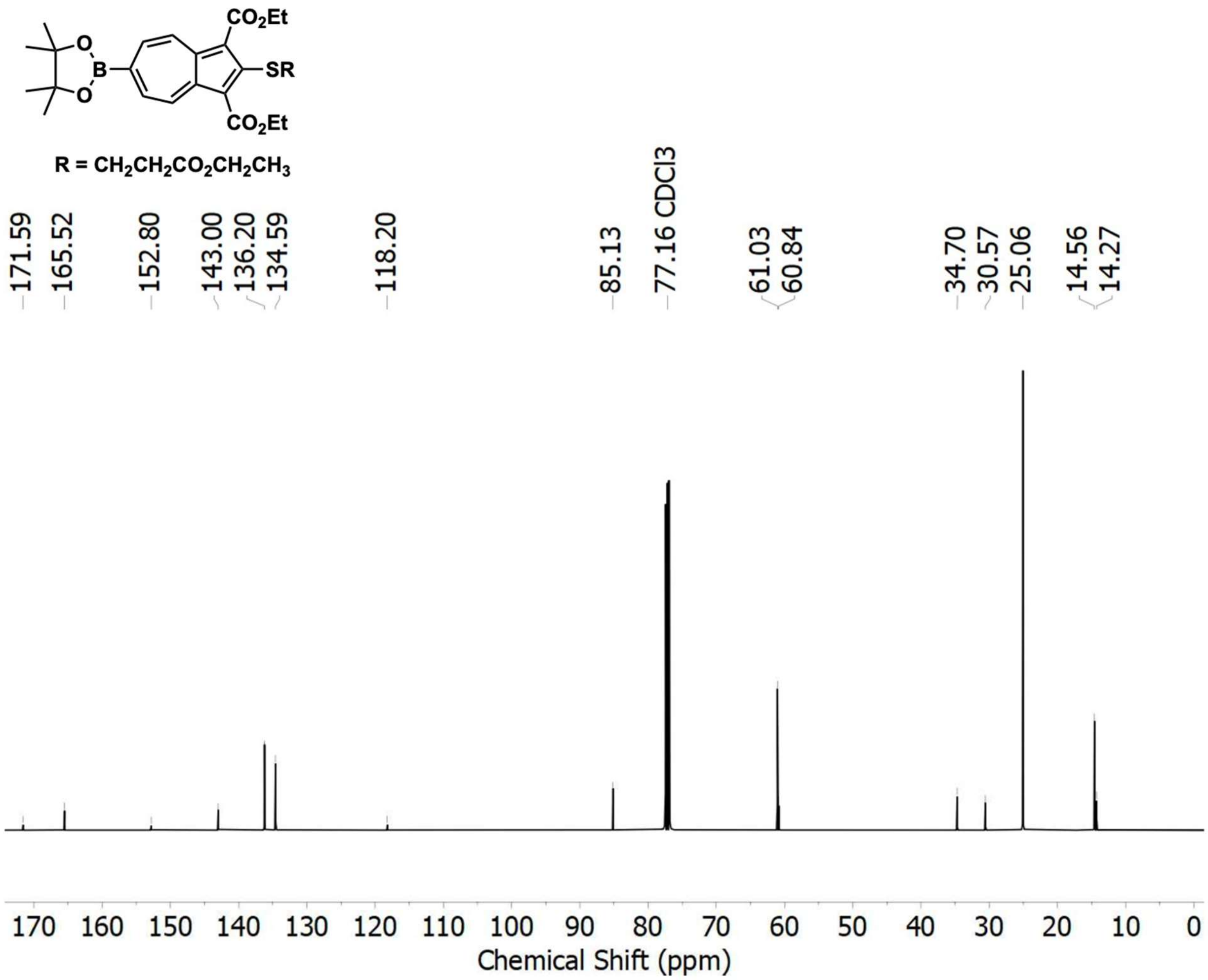

Figure S14. ^{13}C HMR (126 MHz, $CDCl_3$, 25°C) of **8**.

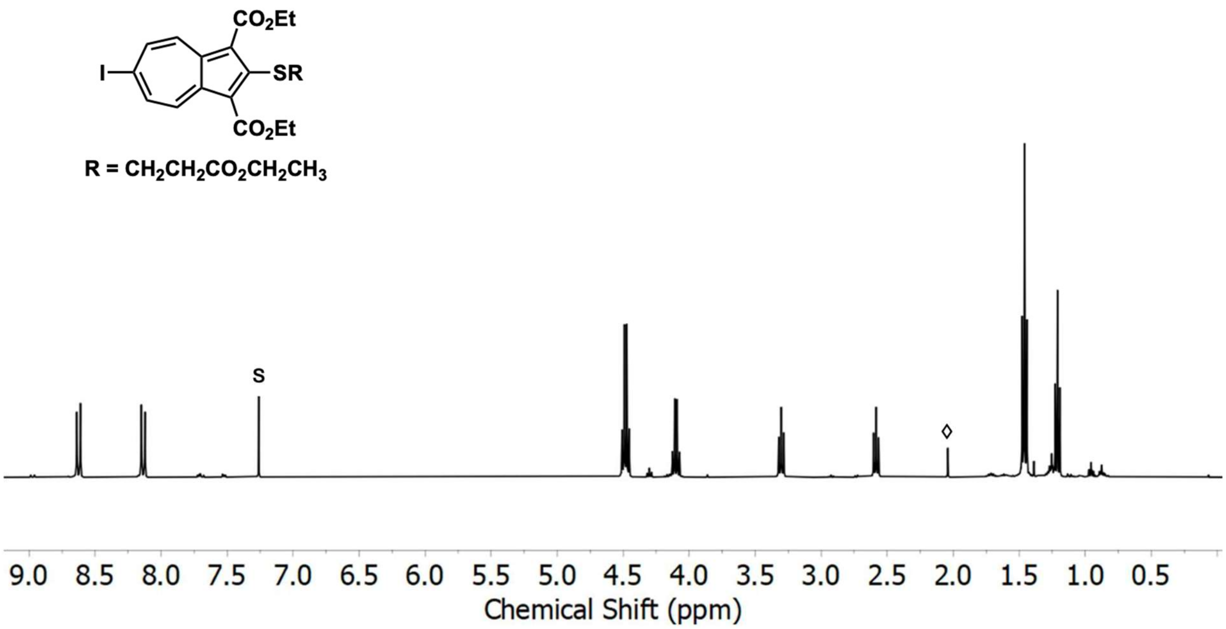

Figure S15. ^{1}H HMR (400 MHz, $CDCl_3$, 25°C) of **9**. S = $CHCl_3$ solvent residual; $^{\Diamond}$acetone impurity.

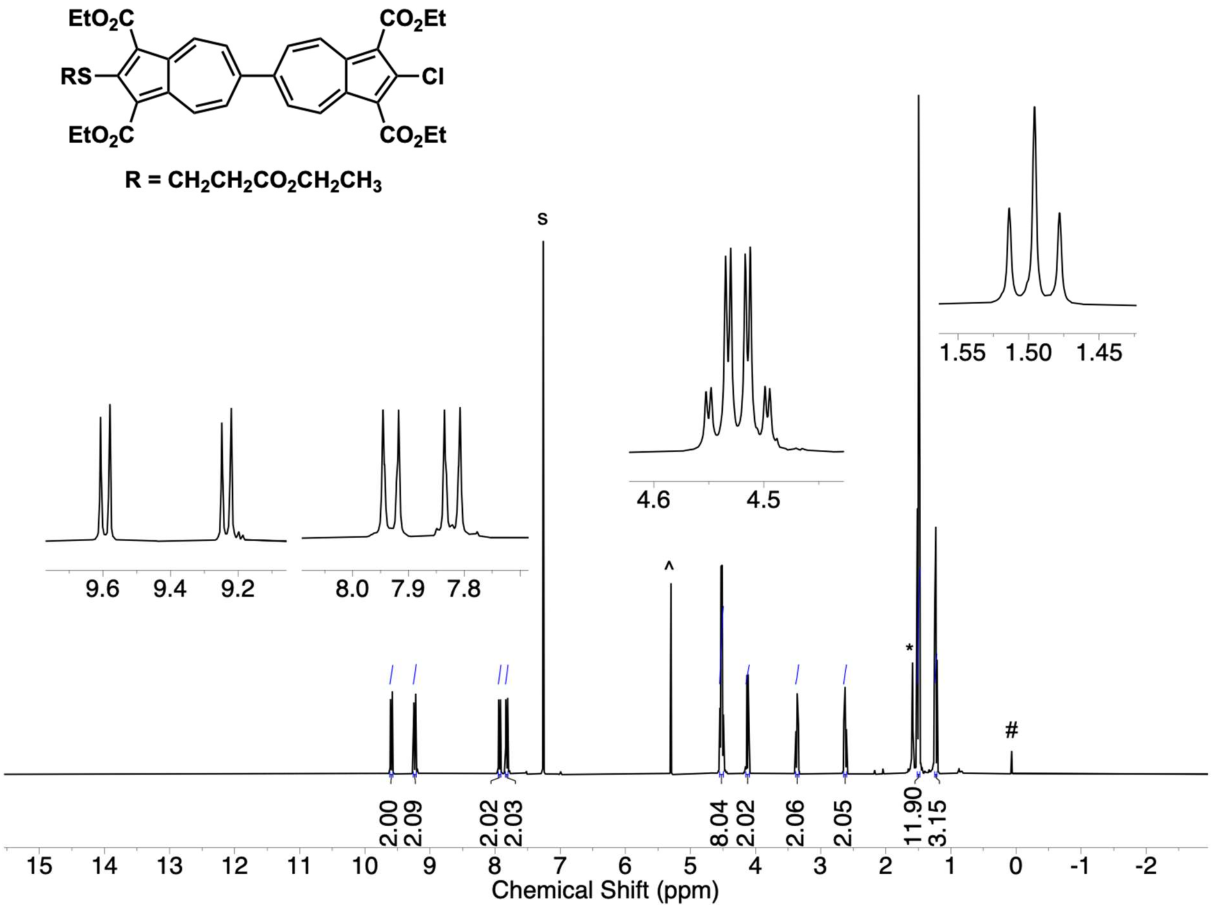

Figure S16. ^{13}C HMR (126 MHz, $CDCl_3$, 25°C) of **10**. S = $CHCl_3$ solvent residual; $^{\wedge}CH_2Cl_2$ impurity; $^{*}H_2O$ impurity in solvent; $^{\#}$silicone grease.

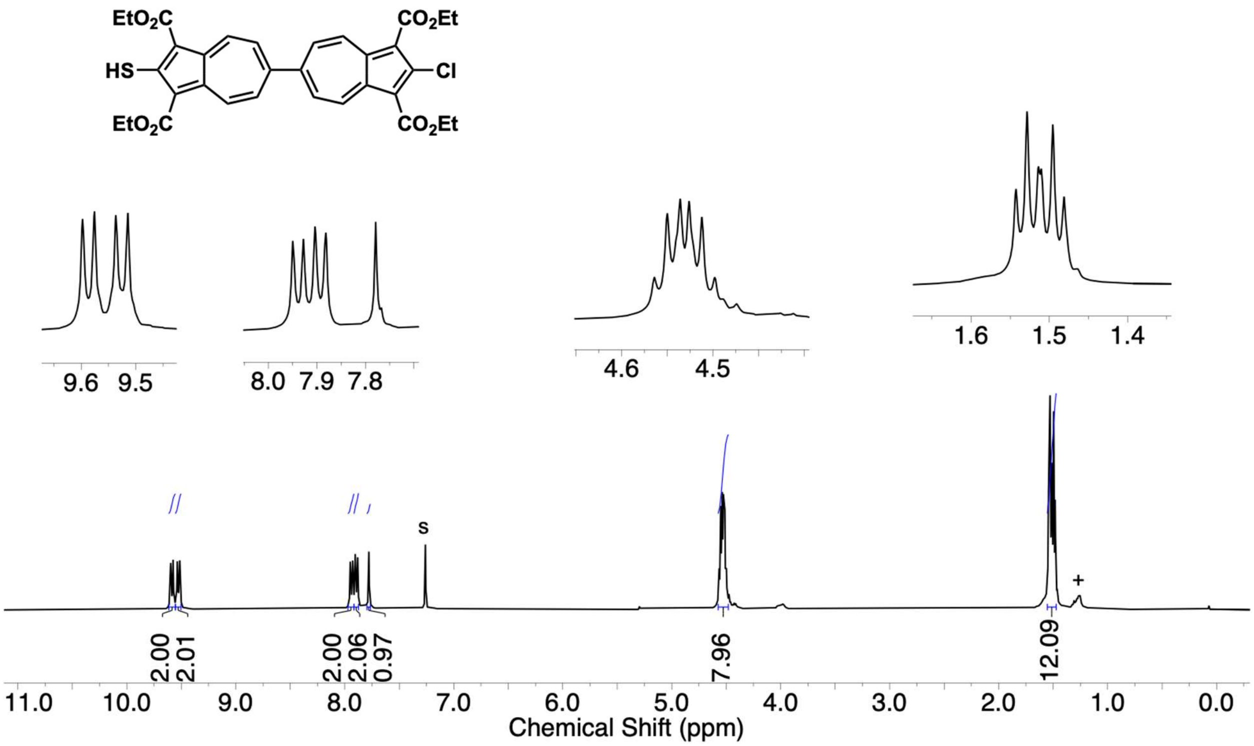

Figure S17. ^{1}H HMR (500 MHz, $CDCl_3$, 25°C) of **11**. S = $CHCl_3$ solvent residual; [+]*n*-pentane impurity.

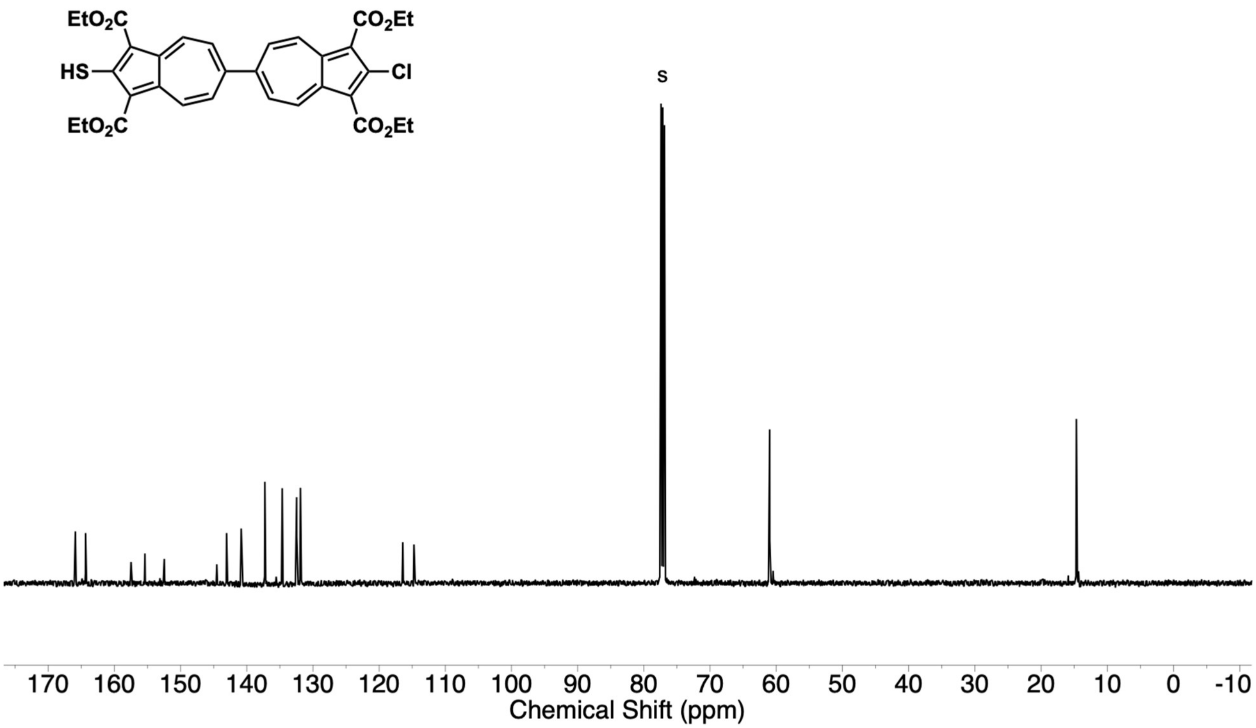

Figure S18. ^{13}C HMR (126 MHz, $CDCl_3$, 25°C) of **11**. S = $CHCl_3$ solvent residual.

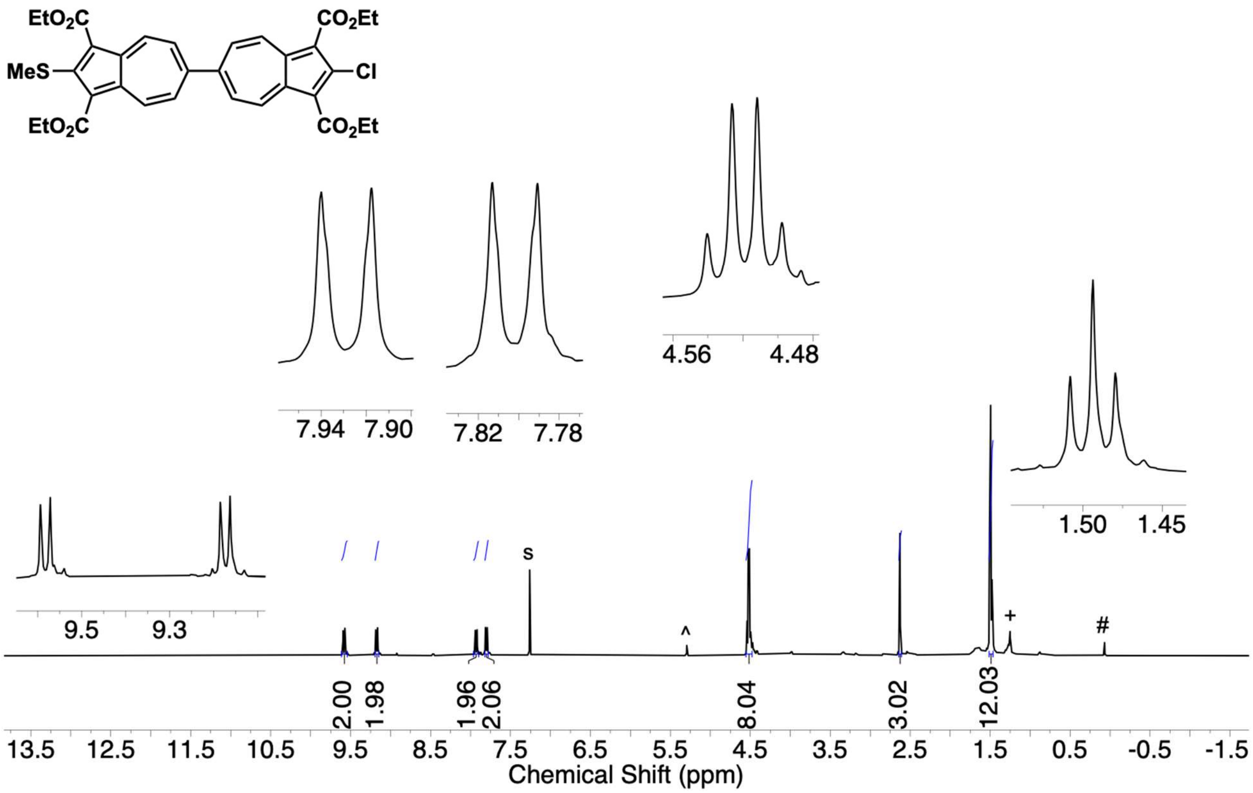

Figure S19. ^{1}H HMR (500 MHz, $CDCl_3$, 25°C) of **12**. S = $CHCl_3$ solvent residual; $^{\wedge}CH_2Cl_2$ impurity; $^+$*n*-pentane impurity; $^\#$silicone grease.

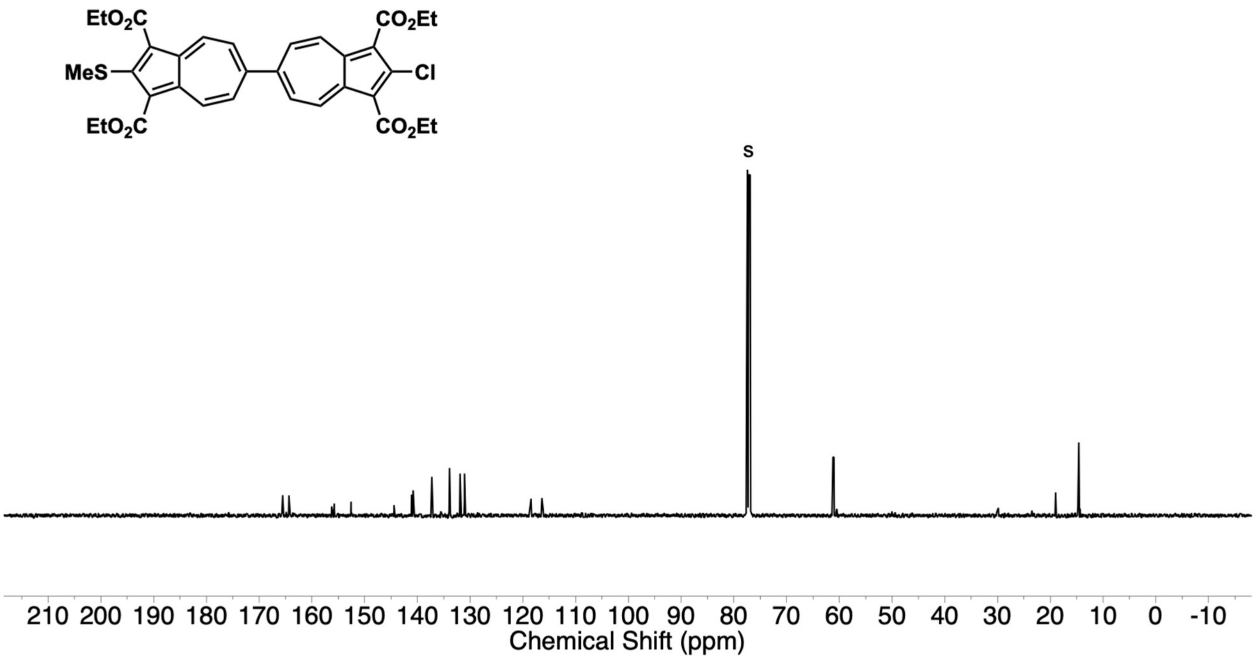

Figure S20. ^{13}C HMR (126 MHz, $CDCl_3$, 25°C) of **12**. S = $CHCl_3$ solvent residual.

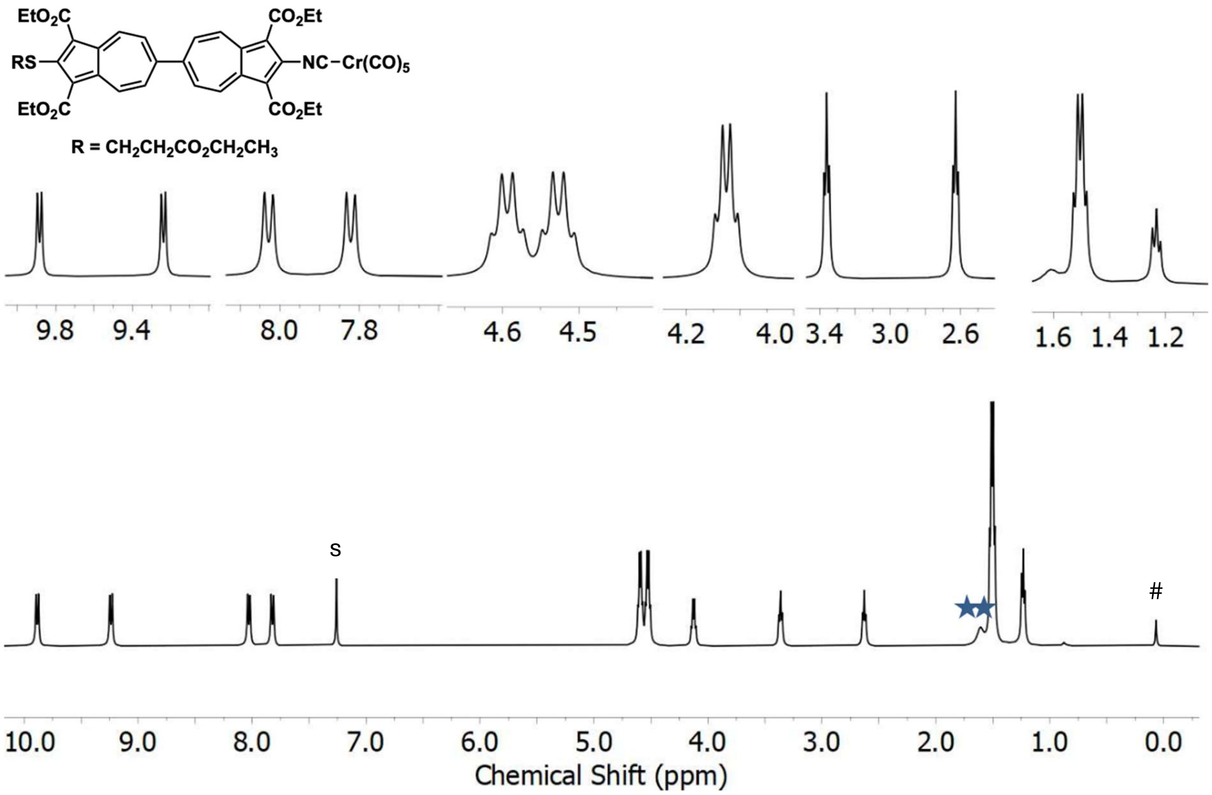

Figure S21. ^{1}H HMR (400 MHz, $CDCl_3$, 25°C) of **14**. S = $CHCl_3$ solvent residual; ★★H_2O impurity in solvent; #silicone grease.

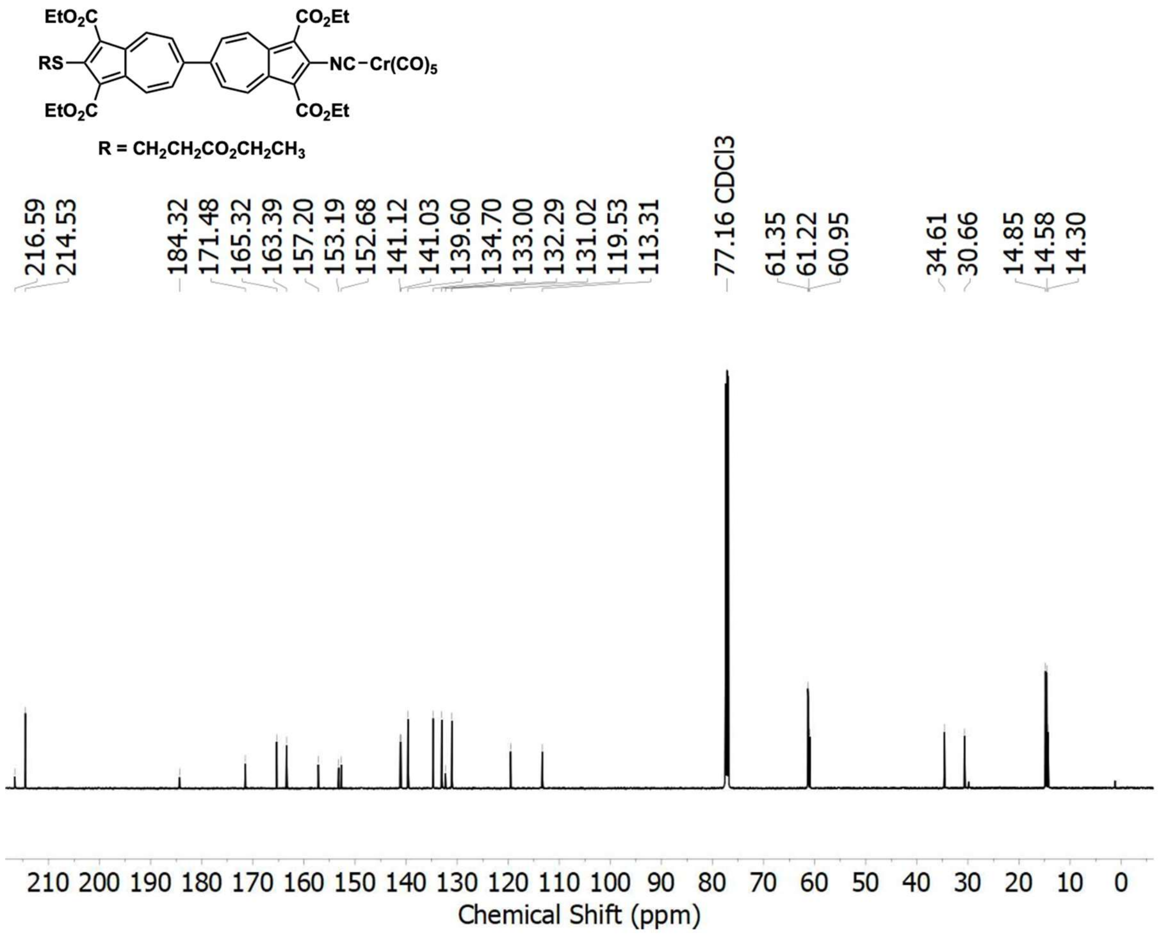

Figure S22. ^{13}C HMR (126 MHz, $CDCl_3$, 25°C) of **14**.

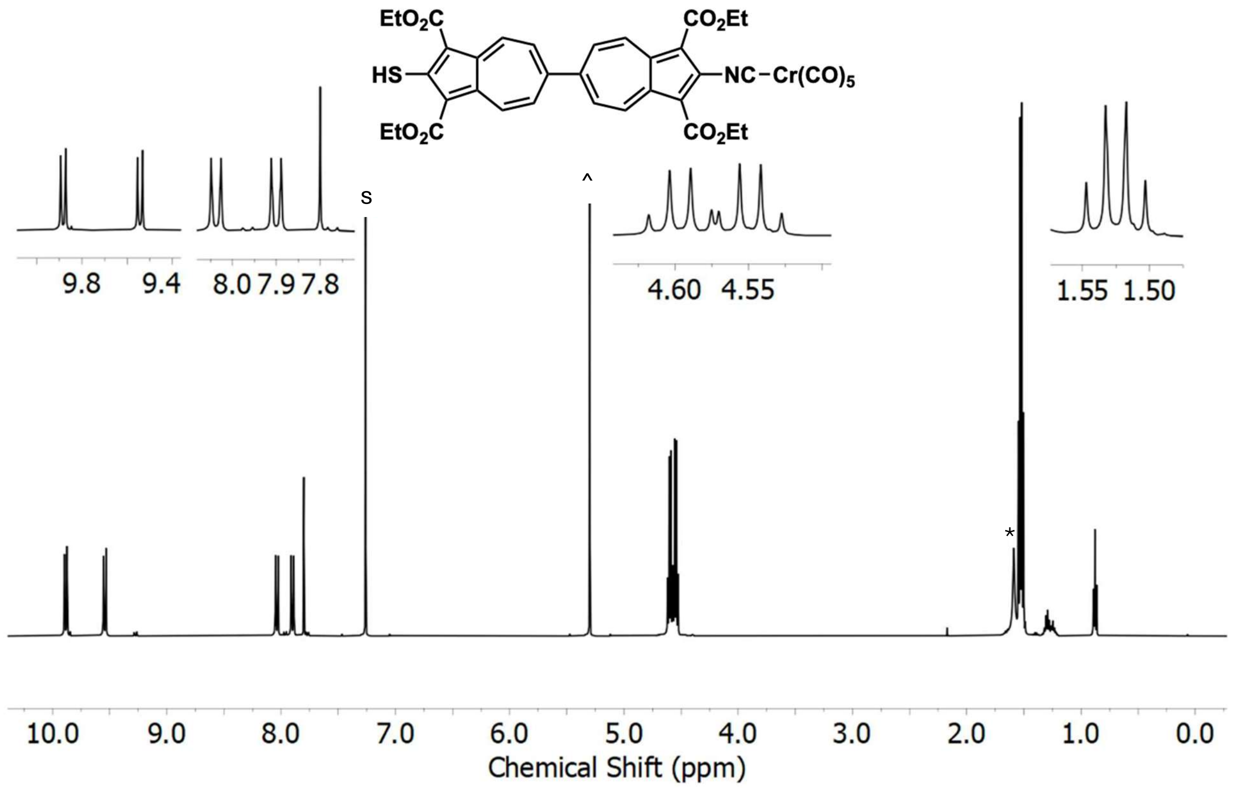

Figure S23. ^{1}H HMR (400 MHz, $CDCl_3$, 25°C) of **15**. S = $CHCl_3$ solvent residual; ^CH_2Cl_2 impurity; *H_2O impurity in solvent.

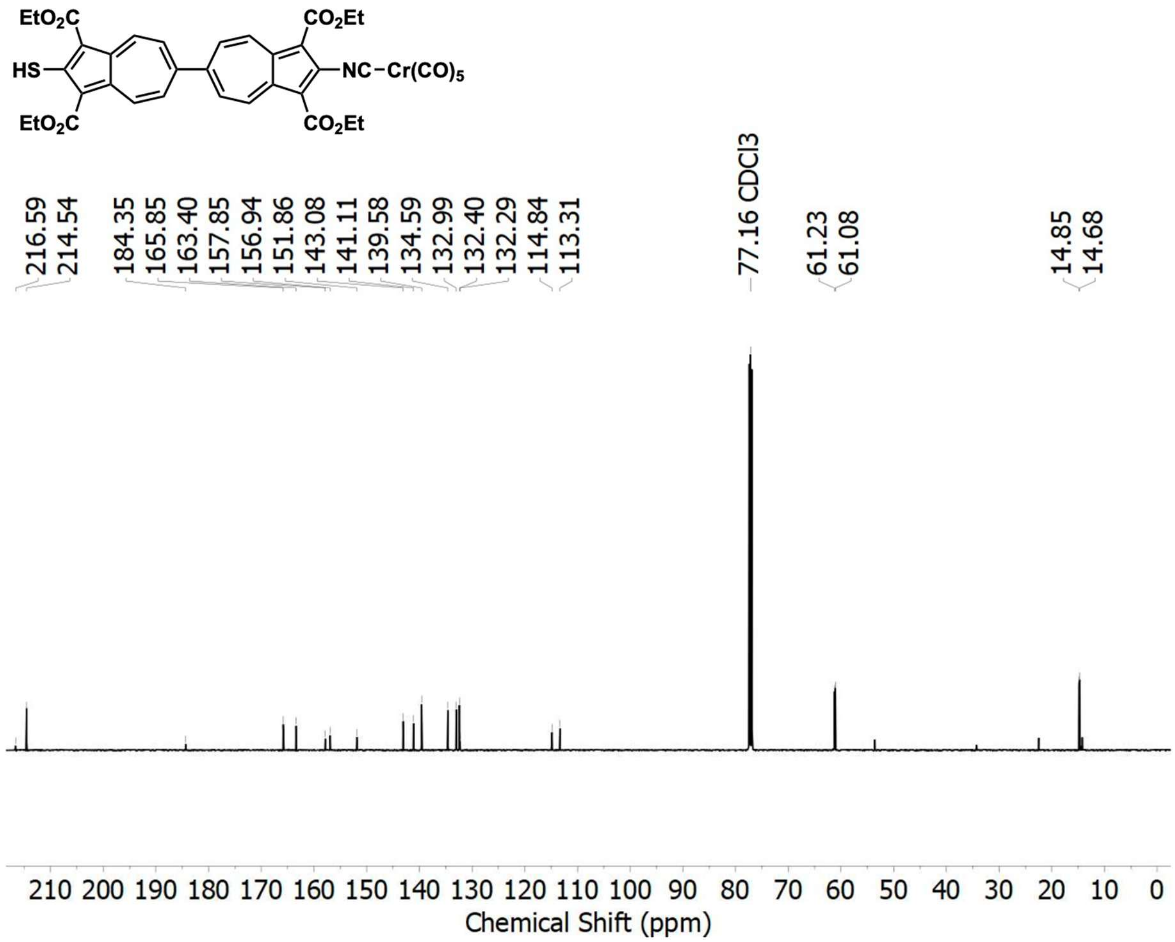

Figure S24. ^{13}C HMR (126 MHz, $CDCl_3$, 25°C) of **15**.

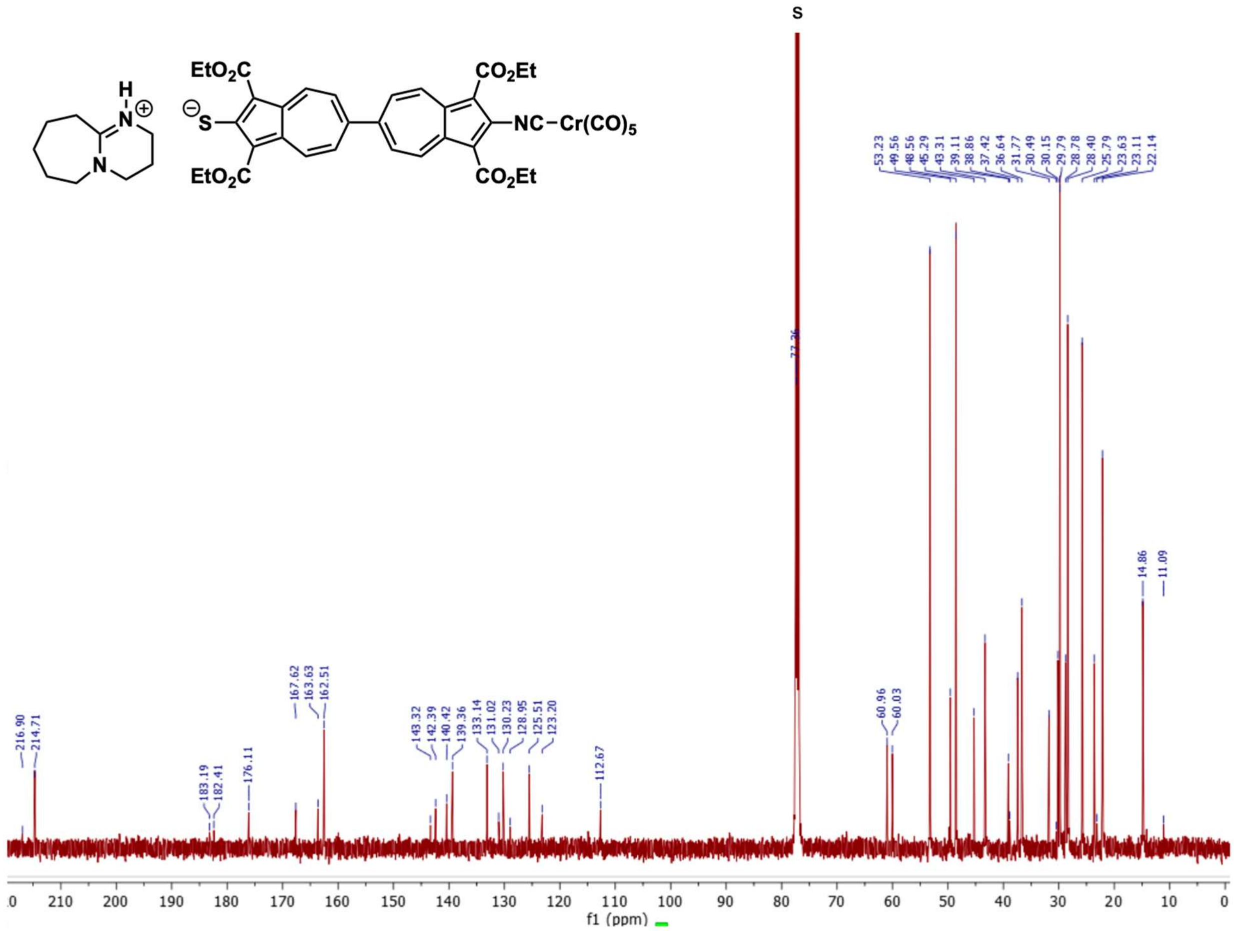

Figure S25. ^{13}C HMR (126 MHz, $CDCl_3$, 25°C) of **15***. S = $CHCl_3$ solvent residual.

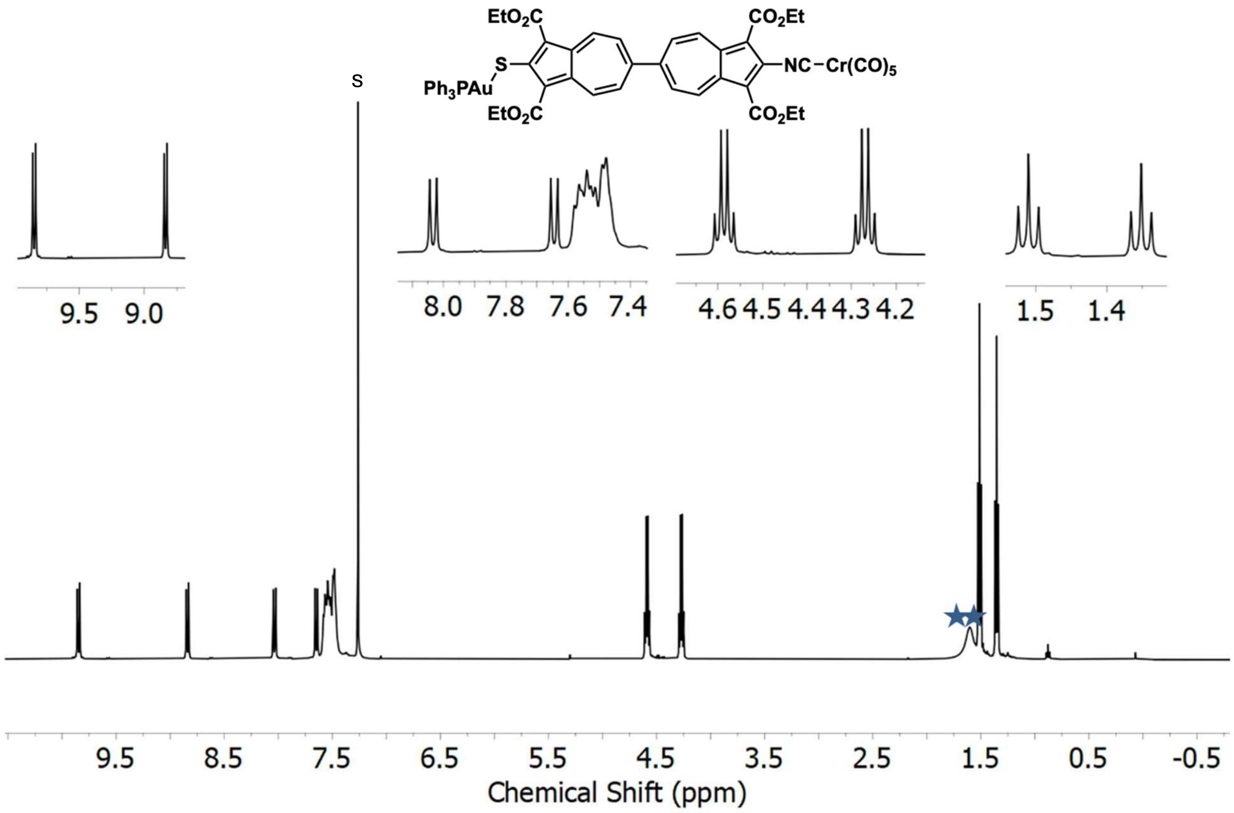

Figure S26. ^{1}H HMR (400 MHz, $CDCl_3$, 25°C) of **16**. S = $CHCl_3$ solvent residual; ★★H_2O impurity in solvent.

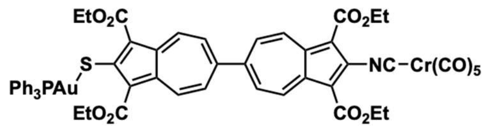

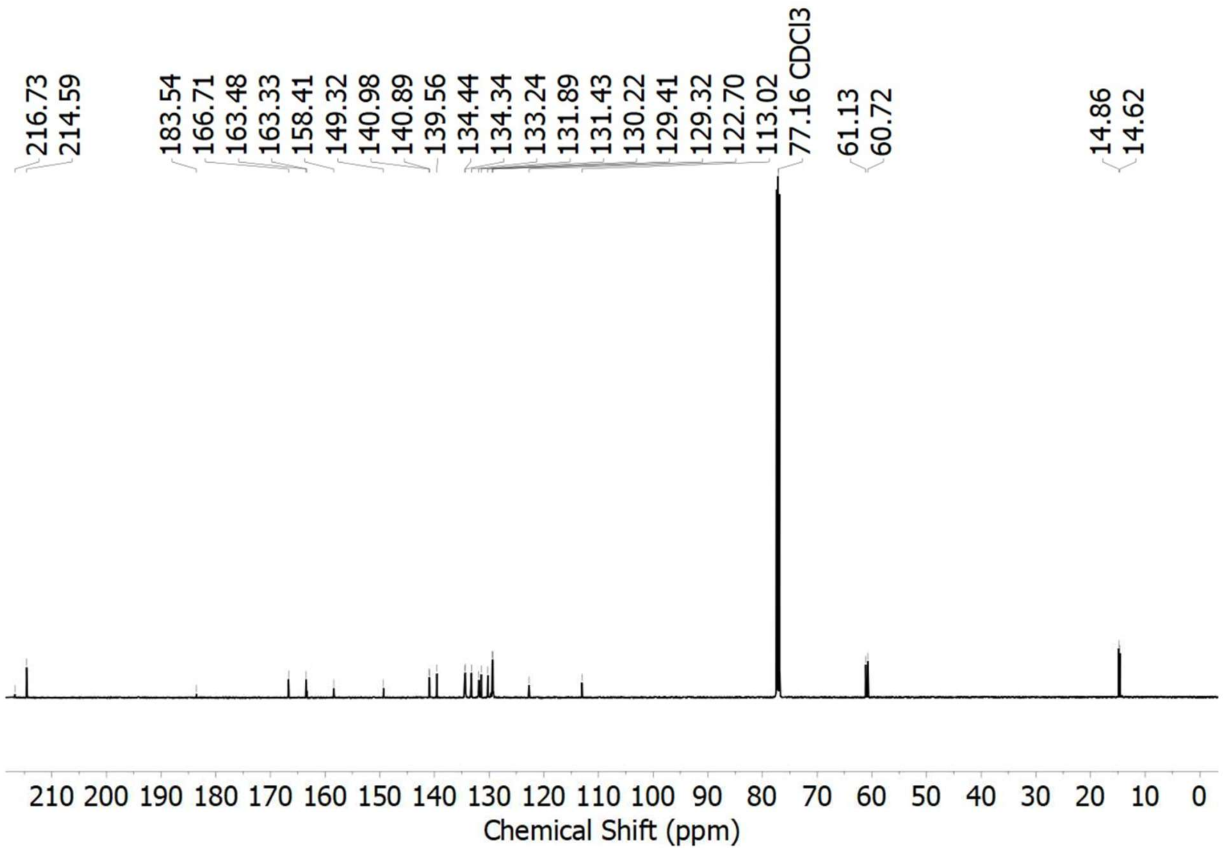

Figure S27. ^{13}C HMR (126 MHz, $CDCl_3$, 25°C) of **16**.

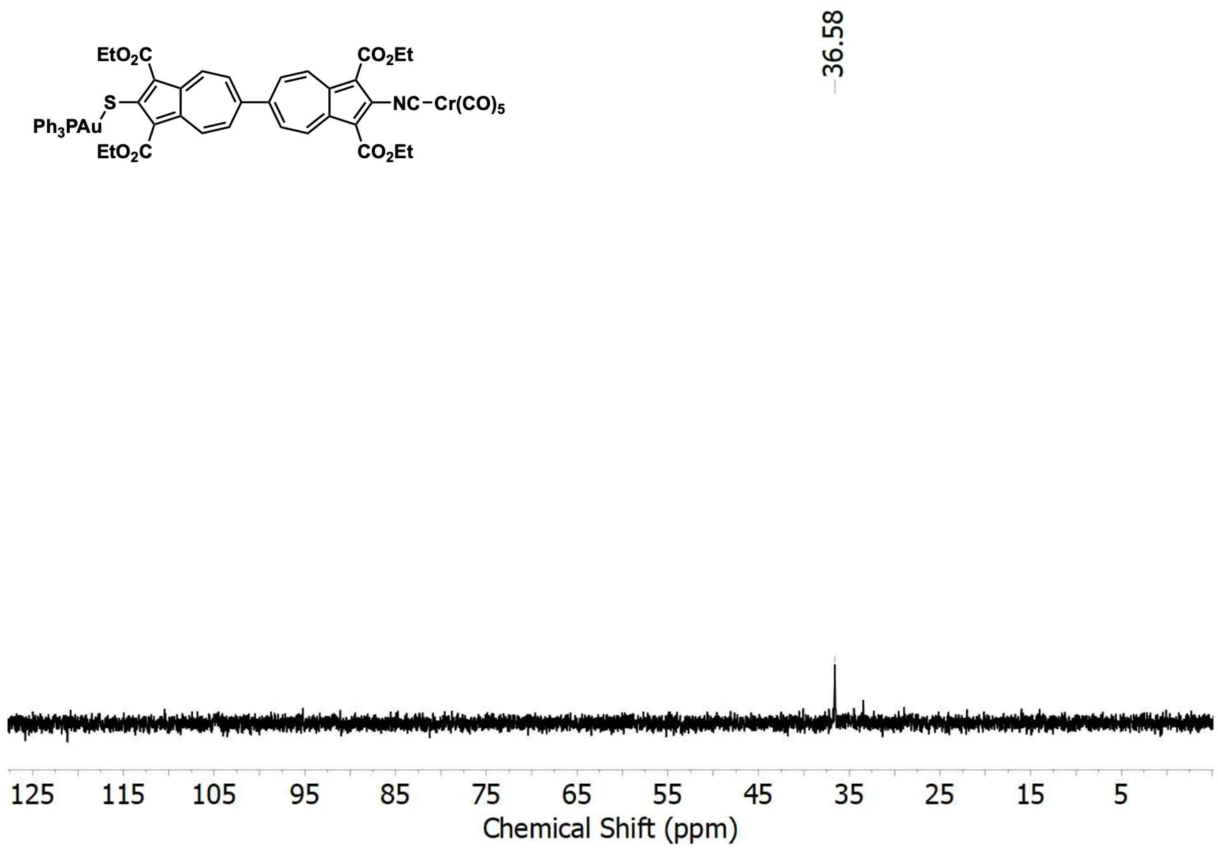

Figure S28. ${}^{31}P\{{}^{1}H\}$ NMR (162 MHz, $CDCl_3$) spectrum of **16**.

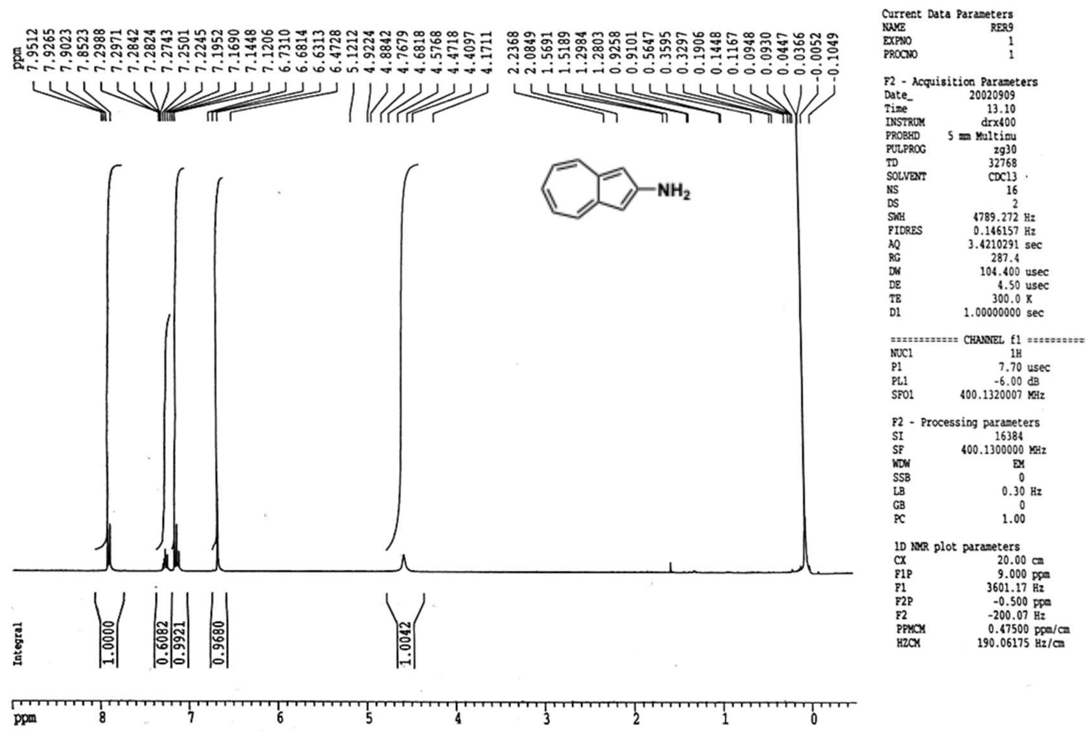

Figure S29. ^{1}H HMR (400 MHz, $CDCl_3$, 25°C) of 2-aminoazulene.

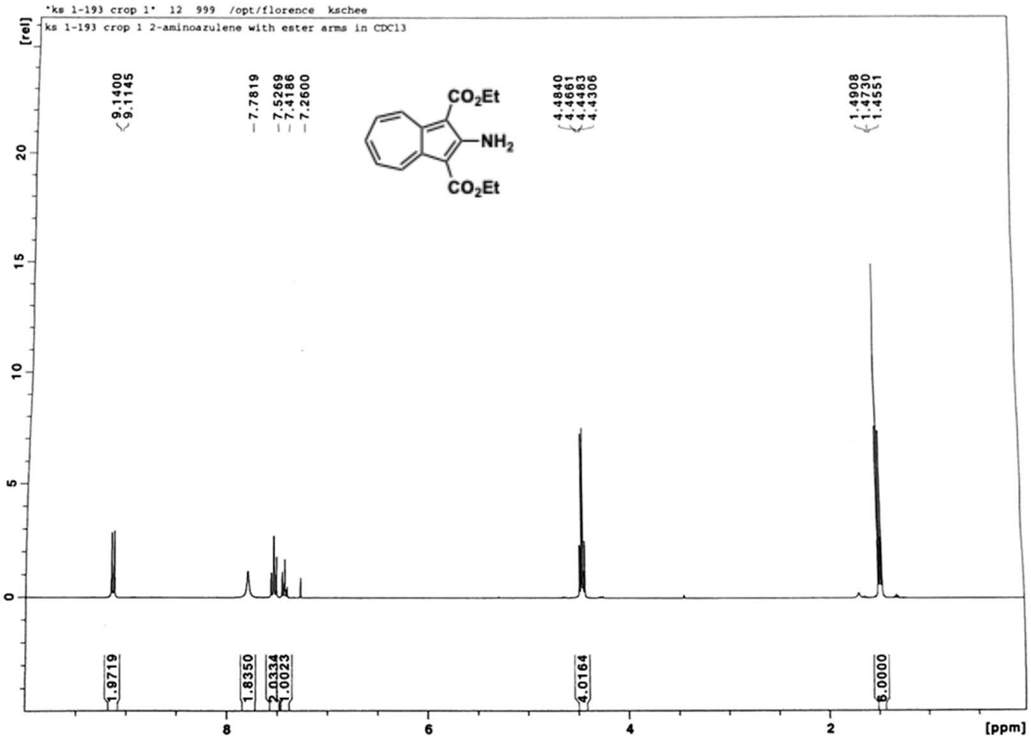

Figure S30. ^{1}H HMR (400 MHz, $CDCl_3$, 25°C) of 2-amino-1,3-diethoxycarbonylazulene.

Table S1. ^{1}H NMR chemical shifts for the amino group(s) of 2-aminoazulenes in $CDCl_3$.

Compound	δ(N*H₂*)	Reference
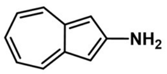	4.58 ppm	Figure S29
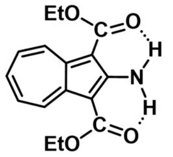	7.78 ppm	Figure S30
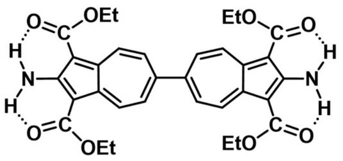	7.88 ppm	5

Table S2. ^{1}H NMR chemical shifts for the mercapto group(s) of 2-mercaptoazulenes in $CDCl_3$.

Compound	δ(S*H*)	Reference
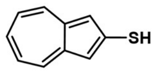	3.88 ppm	6
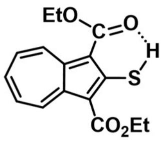	7.71 ppm	6
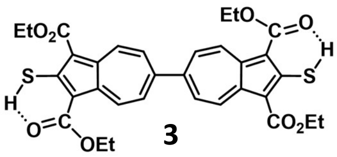 **3**	7.78 ppm	Figure S3

Table S3. ^{13}C NMR chemical shifts for the [NC-Cr(CO)$_5$] moiety in **14**, **15**, **15***, **16**, and **17** in $CDCl_3$.

Compound	δ(^{13}CN)	δ($^{13}CO_{trans}$)	δ($^{13}CO_{cis}$)
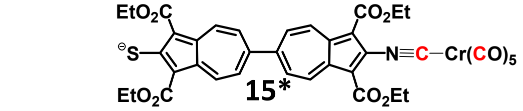 **15***	182.40	216.89	214.69
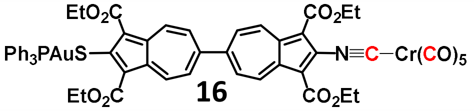 **16**	183.53	216.72	214.58
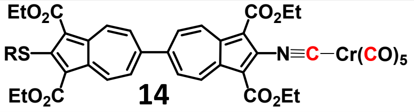 **14**	184.32	216.59	214.52
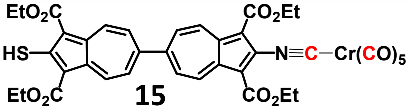 **15**	184.34	216.58	214.53
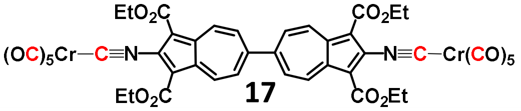 **17**	185.06	216.49	214.48

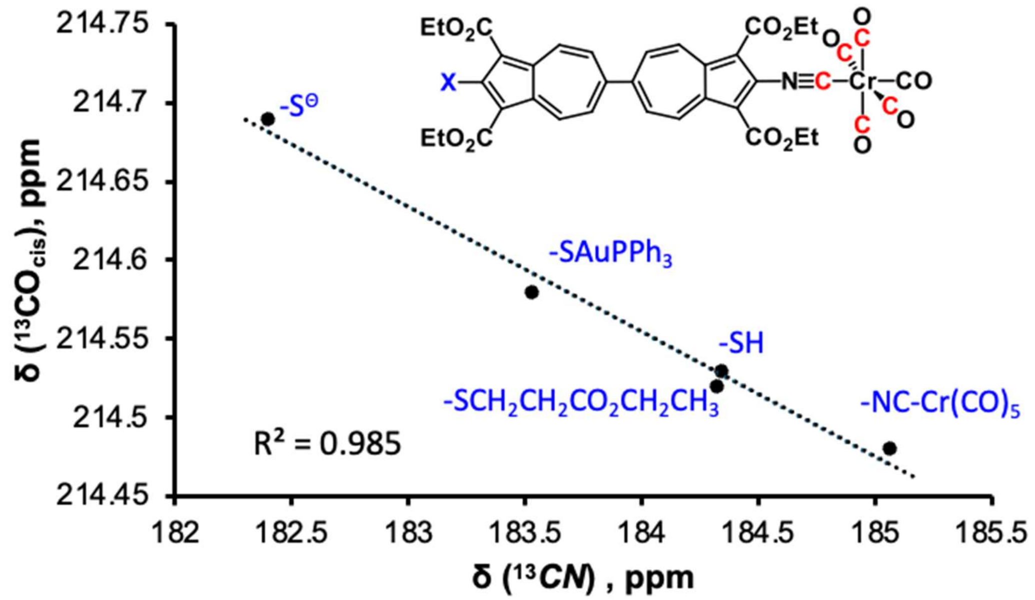

Figure S31. Plot of δ(^{13}CO$_{cis}$) vs. δ(^{13}CN) chemical shifts (in $CDCl_3$) for the [NC-Cr(CO)$_5$] moiety in complexes of functionalized 2-isocyanobiazulene ligands.

C. HRMS

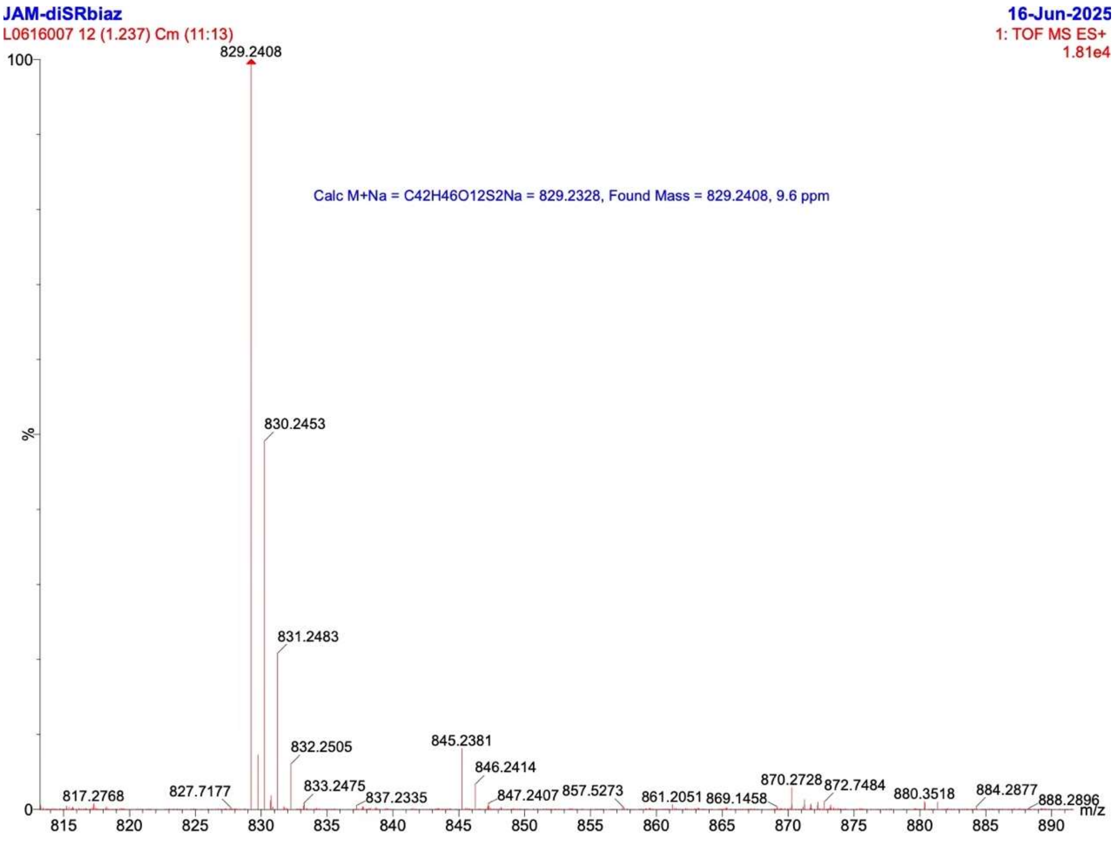

Figure S32. Positive-ion ESI mass spectrum of **2**.

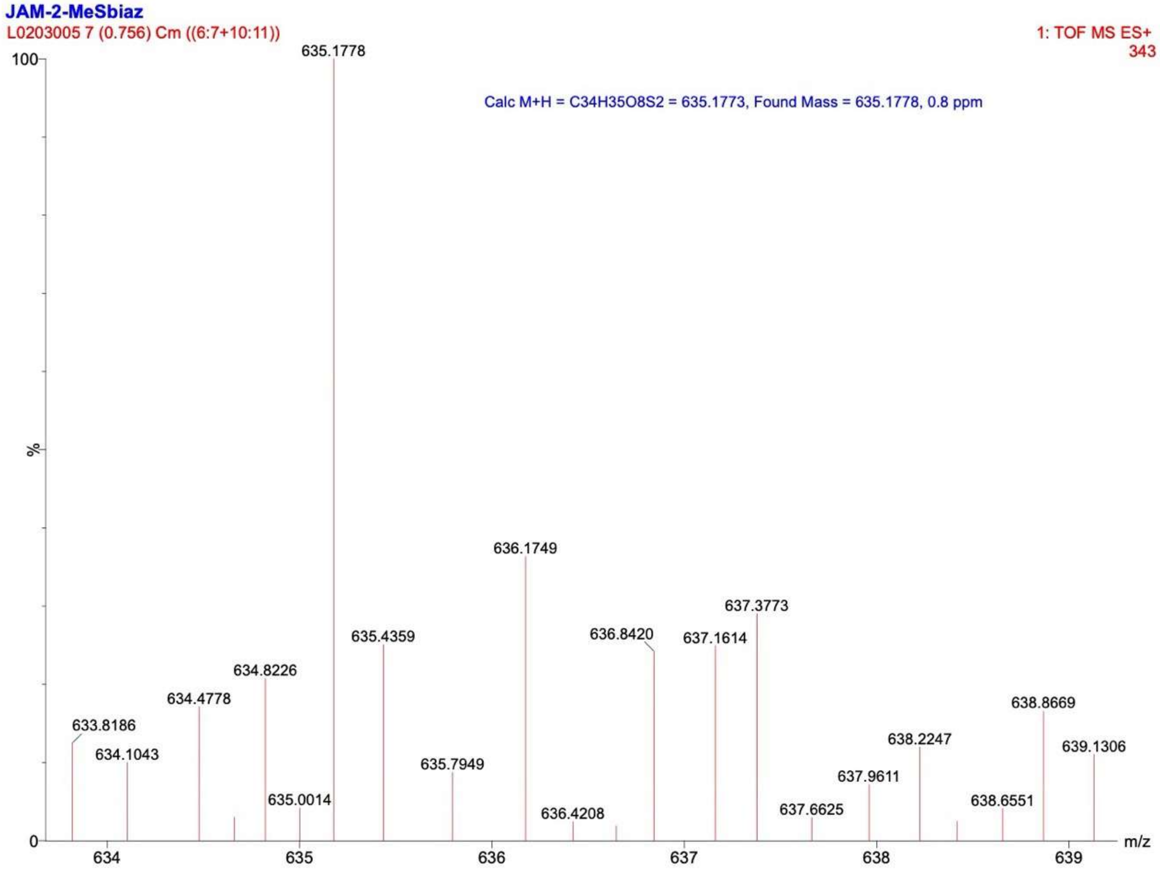

Figure S33. Positive-ion ESI mass spectrum of **4**.

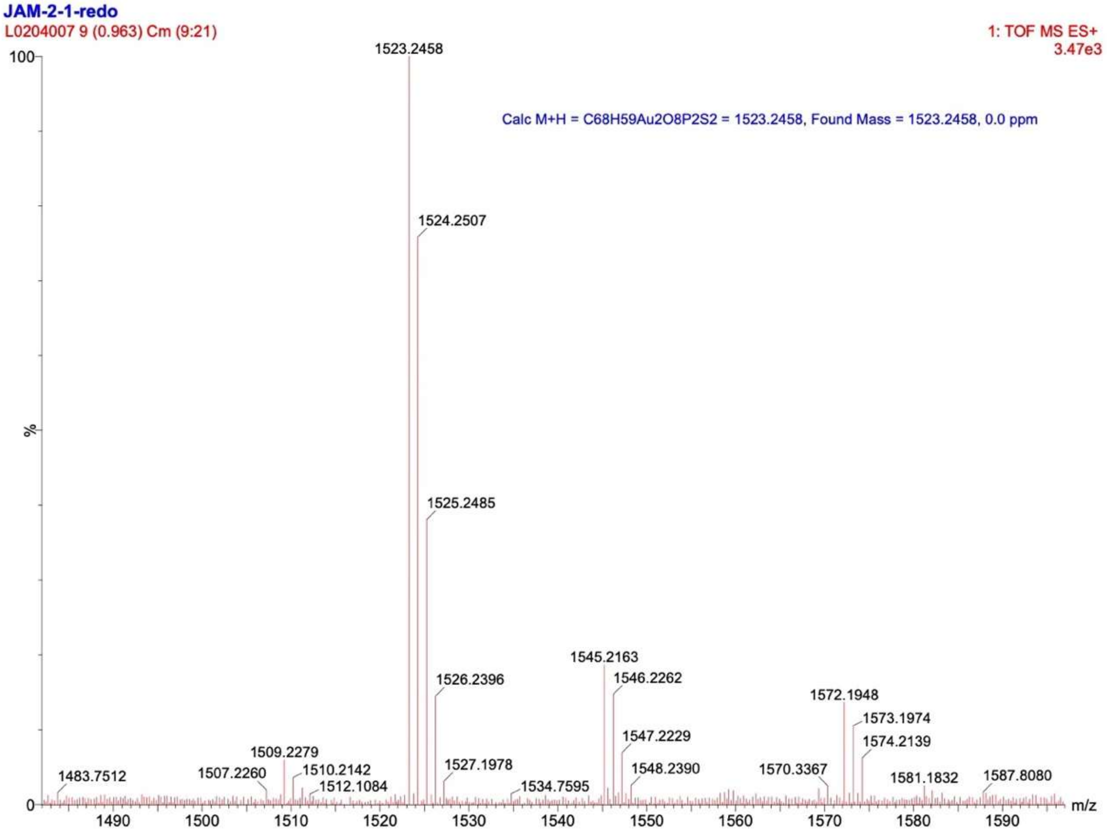

Figure S34. Positive-ion ESI mass spectrum of **5**.

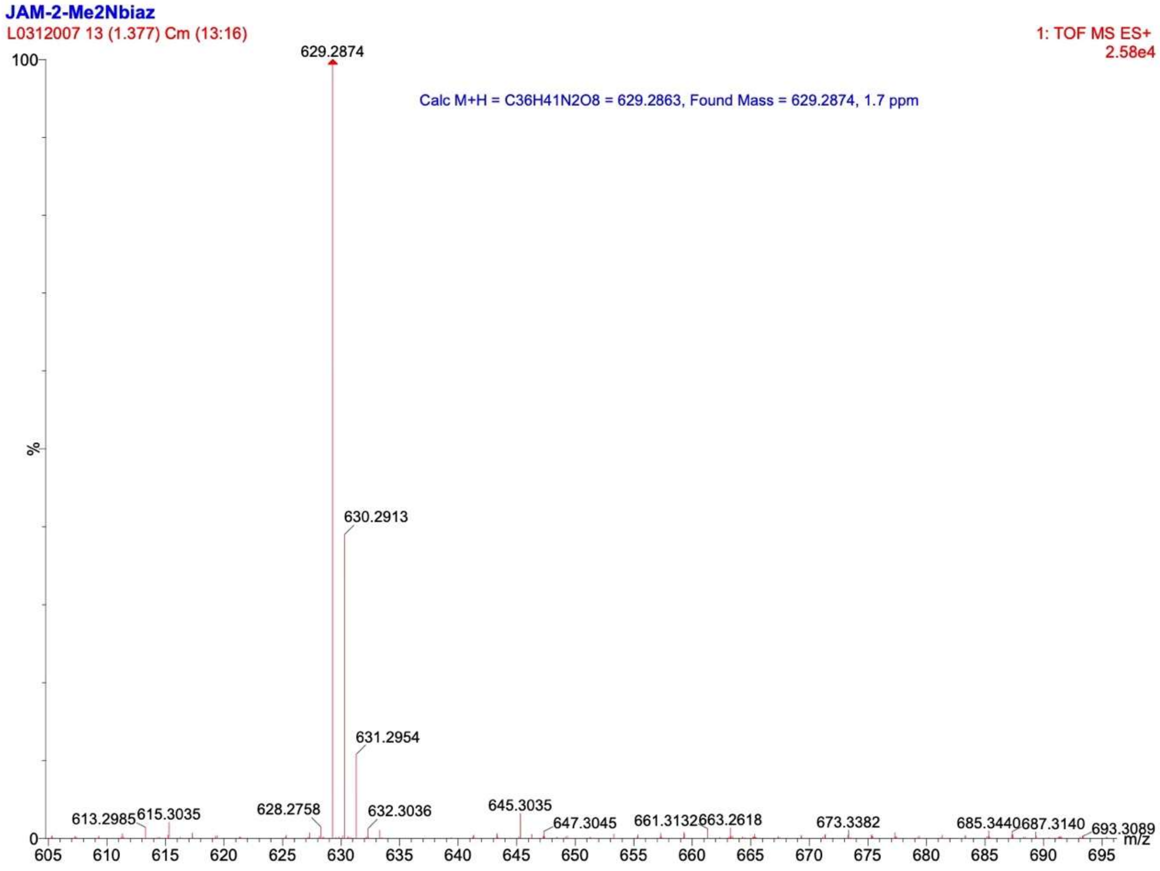

Figure S35. Positive-ion ESI mass spectrum of **6**.

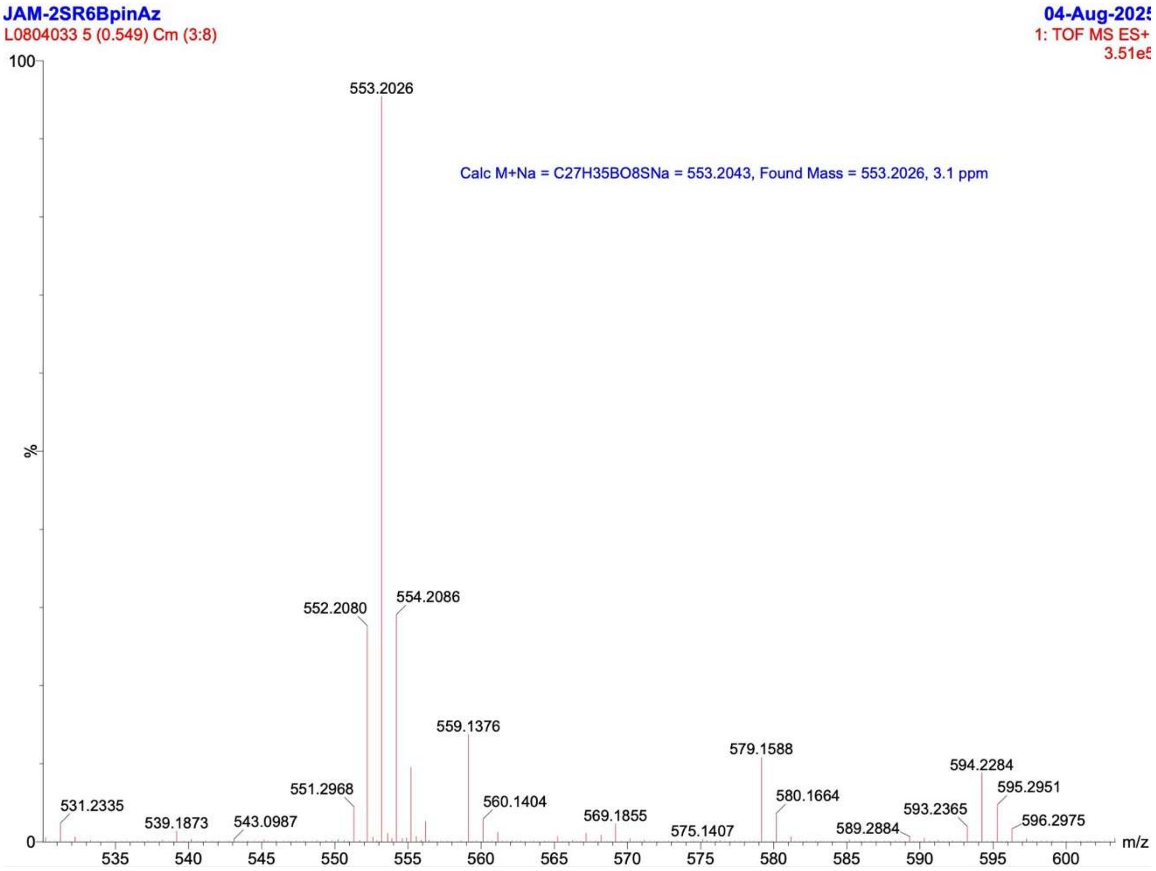

Figure S36. Positive-ion ESI mass spectrum of **8**.

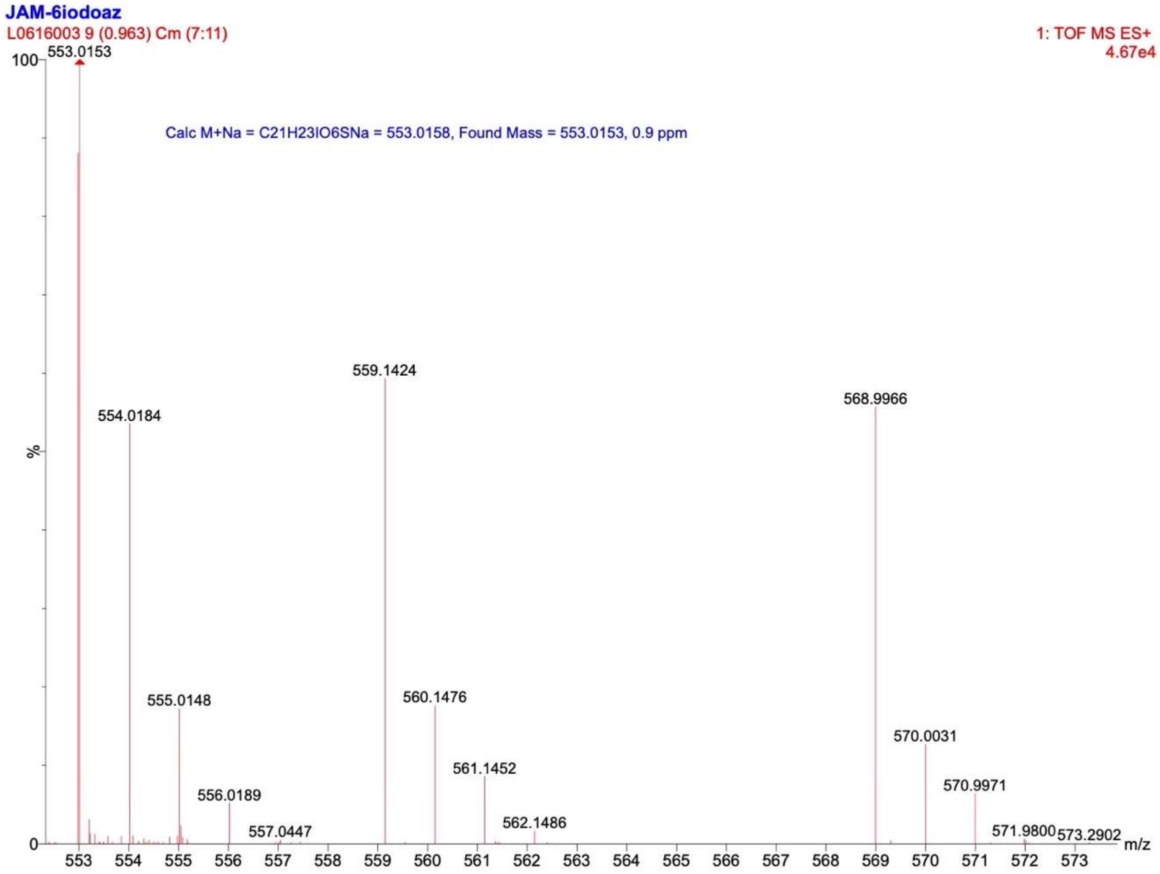

Figure S37. Positive-ion ESI mass spectrum of **9**.

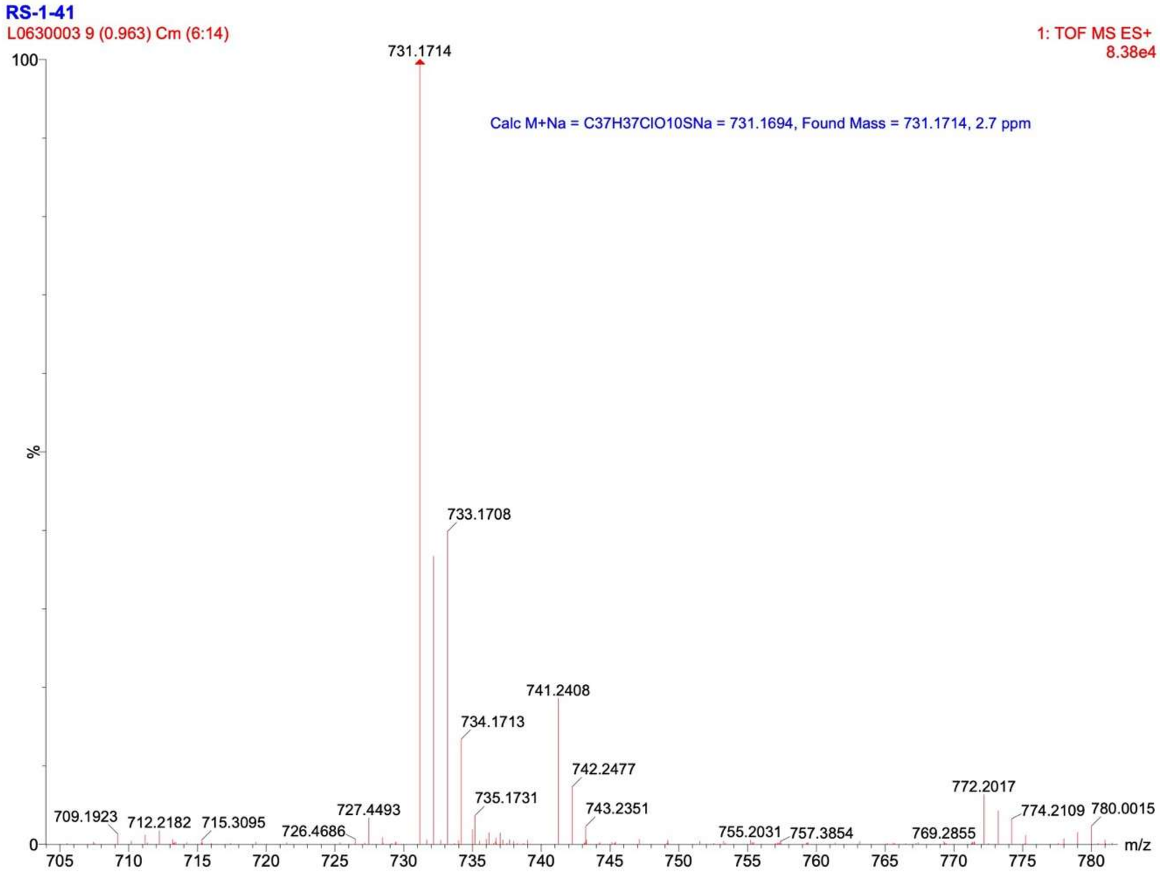

Figure S38. Positive-ion ESI mass spectrum of **10**.

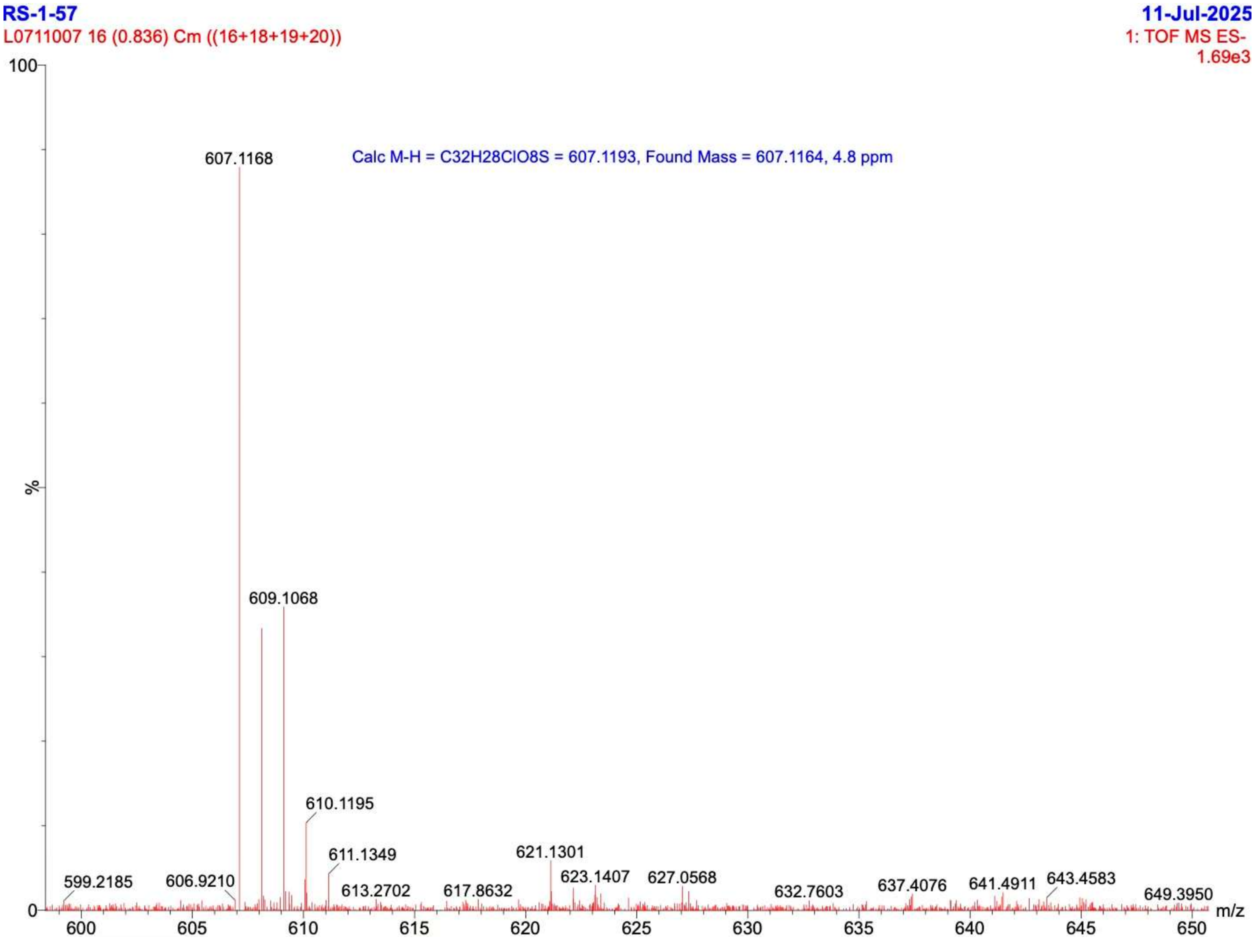

Figure S39. Negative-ion ESI mass spectrum of **11**.

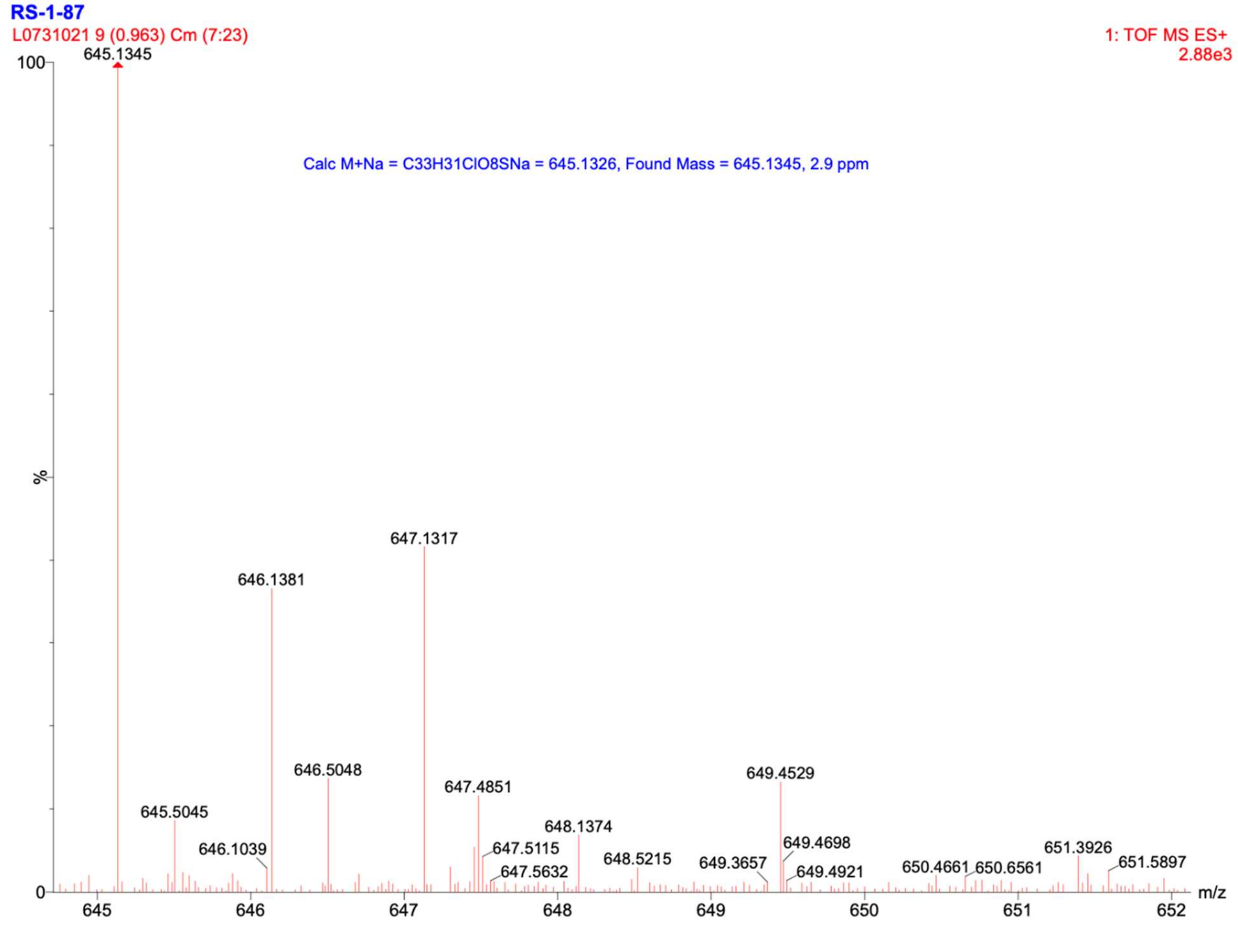

Figure S40. Positive-ion ESI mass spectrum of **12**.

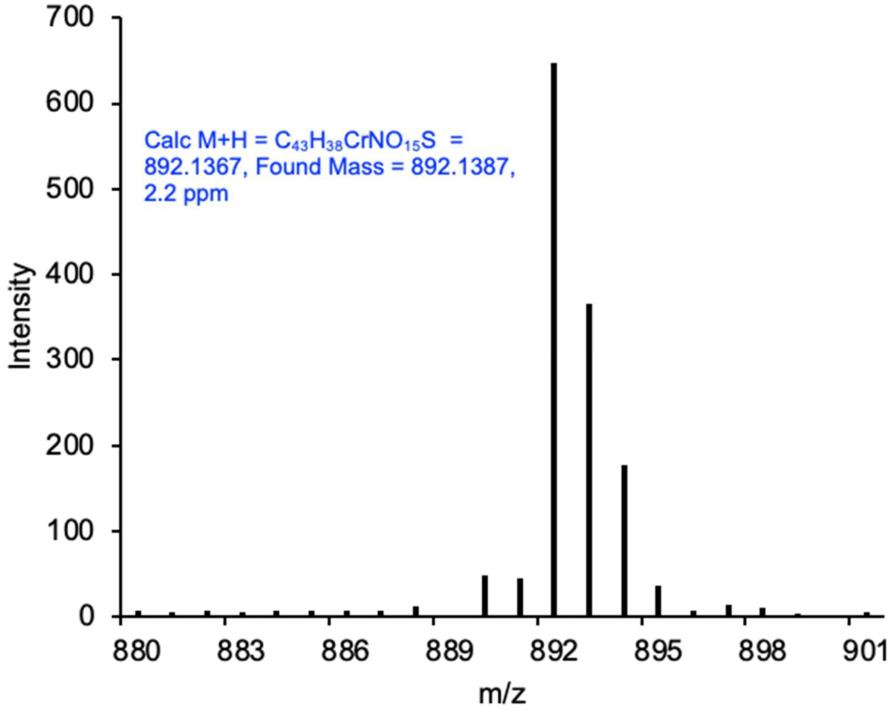

Figure S41. Positive-ion ESI mass spectrum of **14**.

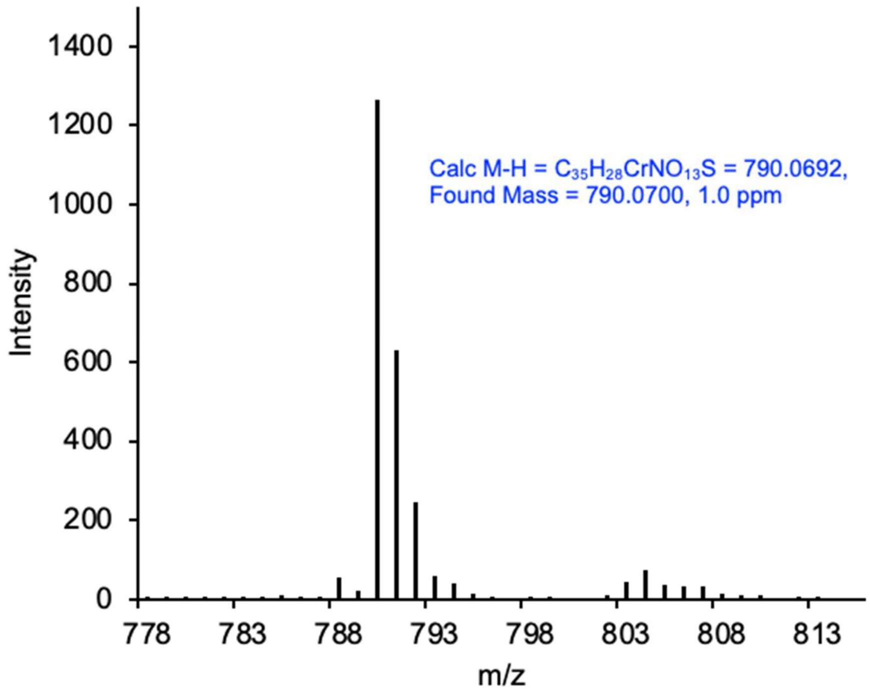

Figure S42. Negative-ion ESI mass spectrum of **15**.

D. Electrochemistry

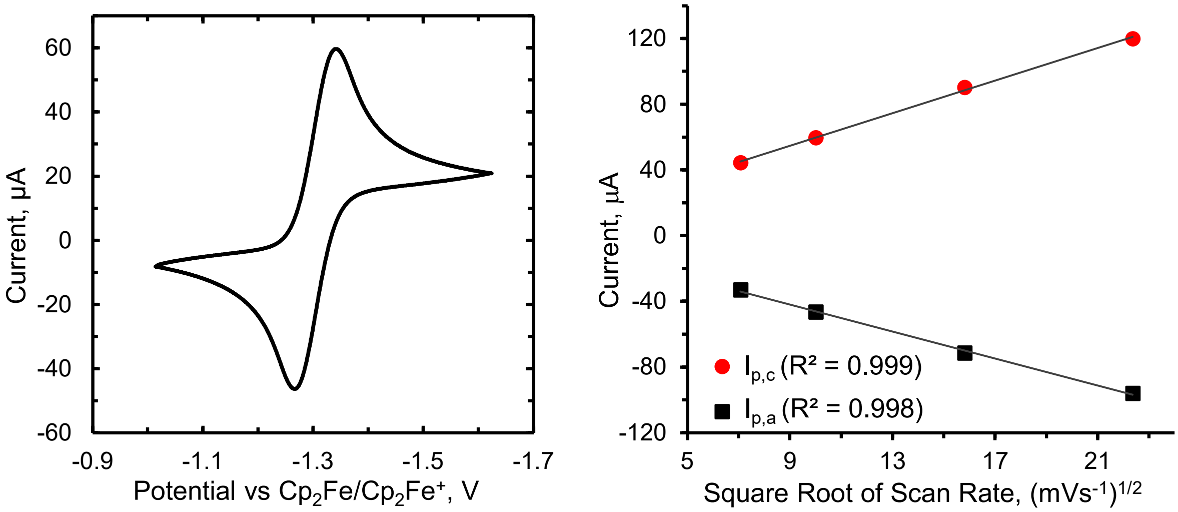

Figure S43. (a) Cyclic voltammogram of **3** in 0.1 M $[^{n}Bu_4N]^+[PF_6]^-/CH_2Cl_2$ vs. external $Cp_2Fe^{0/+}$ at 22°C (scan rate = 100 mV/s). (b) Randles-Sevcik graph of peak currents versus square root of the scan rate for **3** in 0.1 M $[^{n}Bu_4N]^+[PF_6]^-/CH_2Cl_2$.

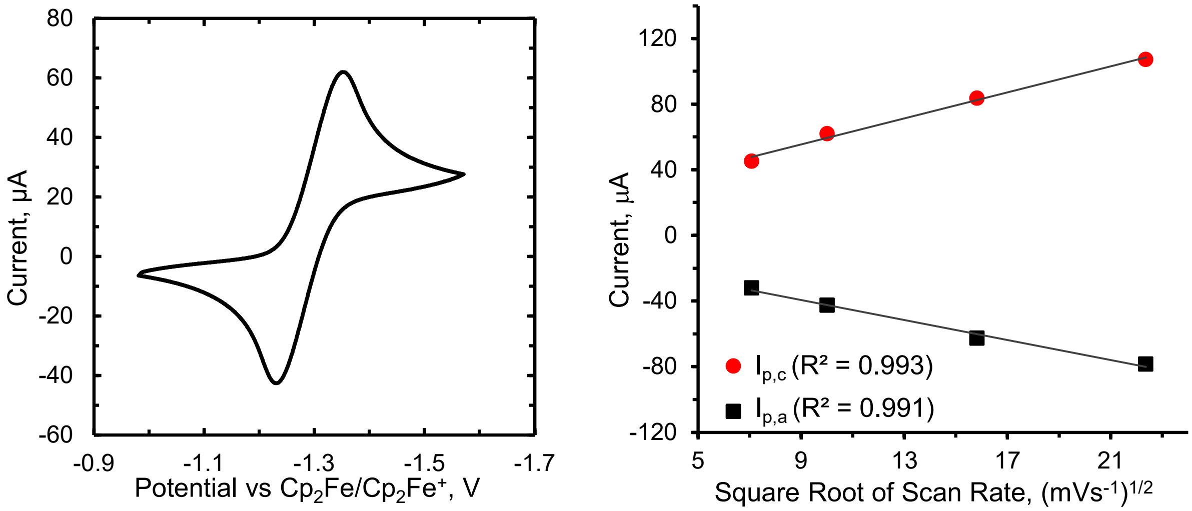

Figure S44. (a) Cyclic voltammogram of **4** in 0.1 M $[^{n}Bu_4N]^+[PF_6]^-/CH_2Cl_2$ vs. external $Cp_2Fe^{0/+}$ at 22°C (scan rate = 100 mV/s). (b) Randles-Sevcik graph of peak currents versus square root of the scan rate for **4** in 0.1 M $[^{n}Bu_4N]^+[PF_6]^-/CH_2Cl_2$.

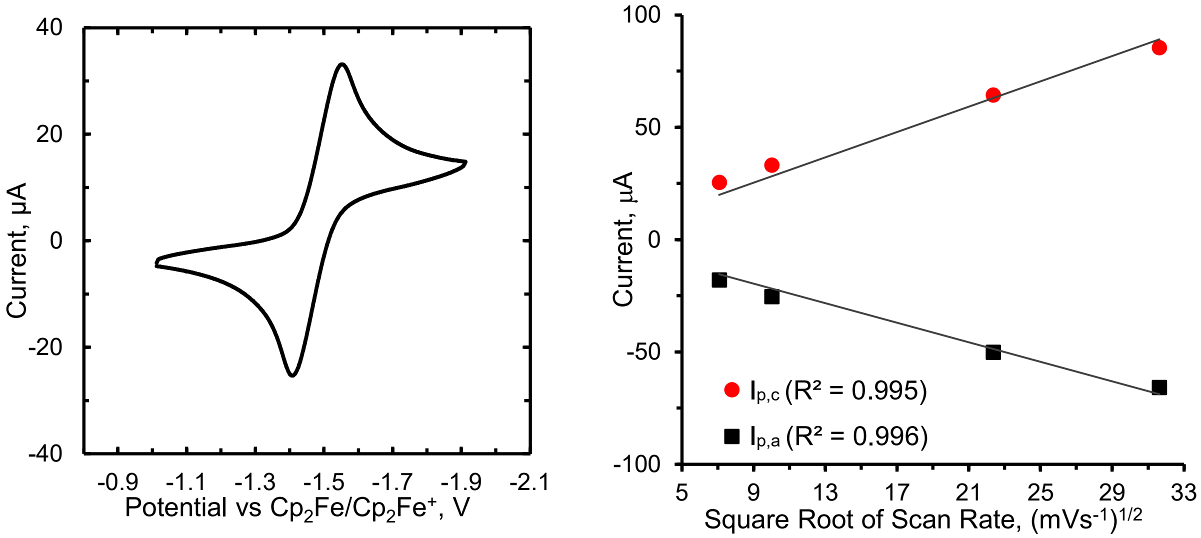

Figure S45. (a) Cyclic voltammogram of **5** in 0.1 M $[^{n}Bu_4N]^{+}[PF_6]^{-}/CH_2Cl_2$ vs. external $Cp_2Fe^{0/+}$ at 22°C (scan rate = 100 mV/s). (b) Randles-Sevcik graph of peak currents versus square root of the scan rate for **5** in 0.1 M $[^{n}Bu_4N]^{+}[PF_6]^{-}/CH_2Cl_2$.

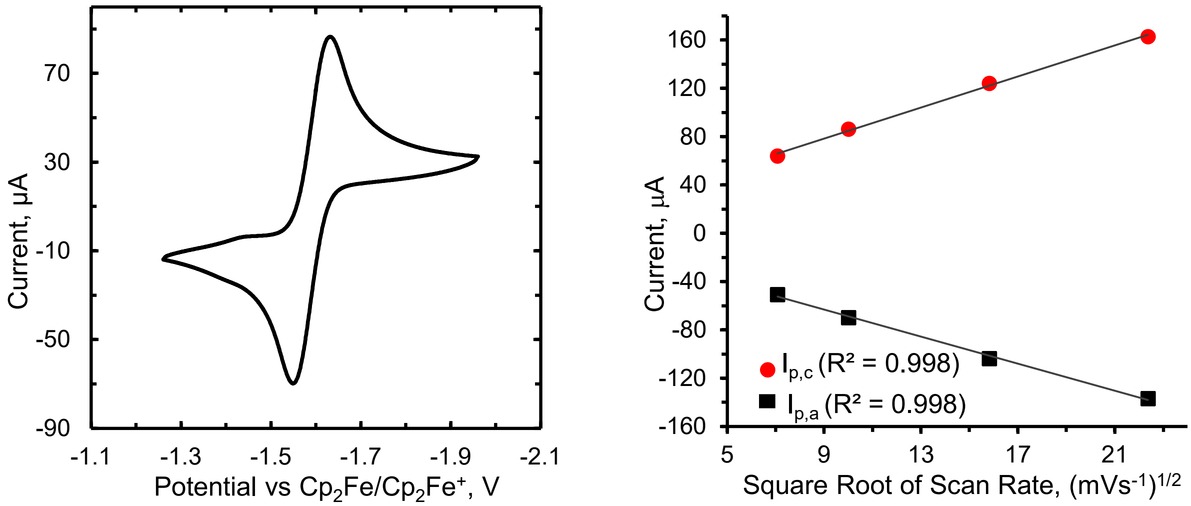

Figure S46. (a) Cyclic voltammogram of **6** in 0.1 M $[^{n}Bu_4N]^{+}[PF_6]^{-}/CH_2Cl_2$ vs. external $Cp_2Fe^{0/+}$ at 22°C (scan rate = 100 mV/s). (b) Randles-Sevcik graph of peak currents versus square root of the scan rate for **6** in 0.1 M $[^{n}Bu_4N]^{+}[PF_6]^{-}/CH_2Cl_2$.

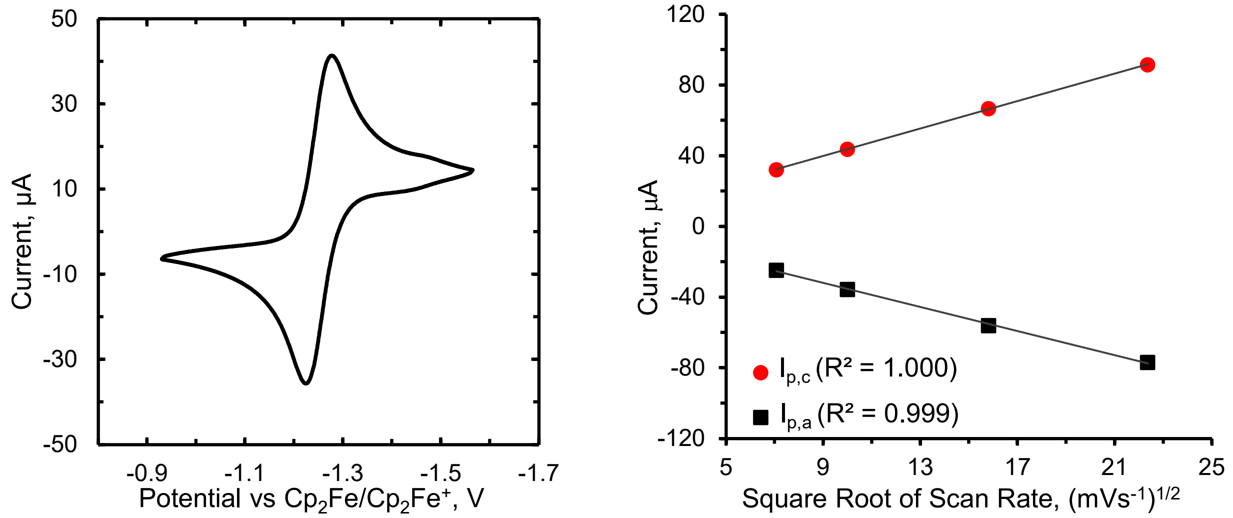

Figure S47. (a) Cyclic voltammogram of **11** in 0.1 M $[^nBu_4N]^+[PF_6]^-/CH_2Cl_2$ vs. external $Cp_2Fe^{0/+}$ at 22°C (scan rate = 100 mV/s). (b) Randles-Sevcik graph of peak currents versus square root of the scan rate for **11** in 0.1 M $[^nBu_4N]^+[PF_6]^-/CH_2Cl_2$.

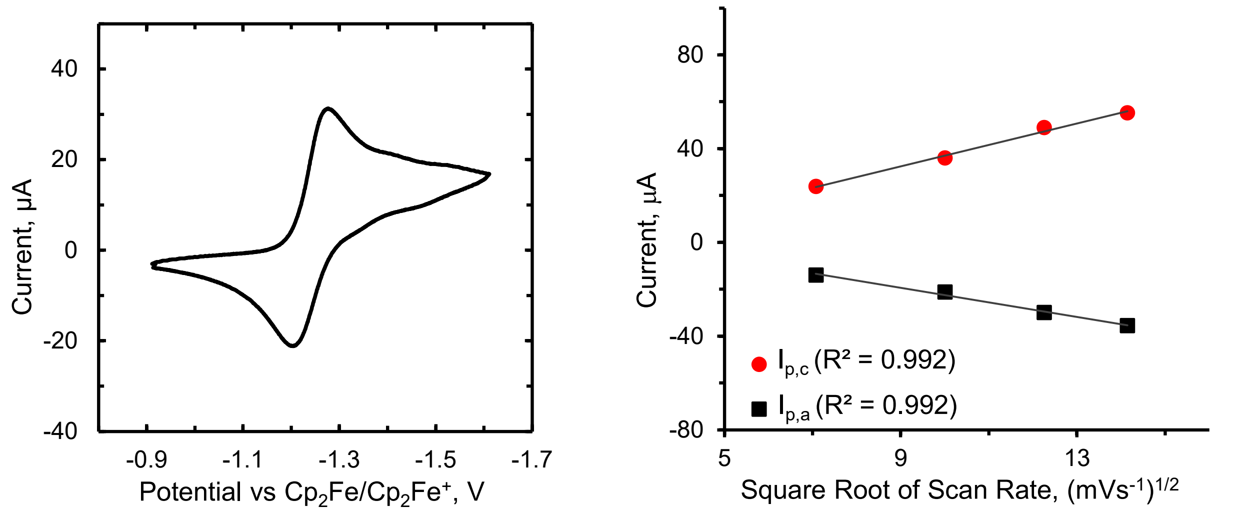

Figure S48. (a) Cyclic voltammogram of **12** in 0.1 M $[^nBu_4N]^+[PF_6]^-/CH_2Cl_2$ vs. external $Cp_2Fe^{0/+}$ at 22°C (scan rate = 100 mV/s). (b) Randles-Sevcik graph of peak currents versus square root of the scan rate for **12** in 0.1 M $[^nBu_4N]^+[PF_6]^-/CH_2Cl_2$.

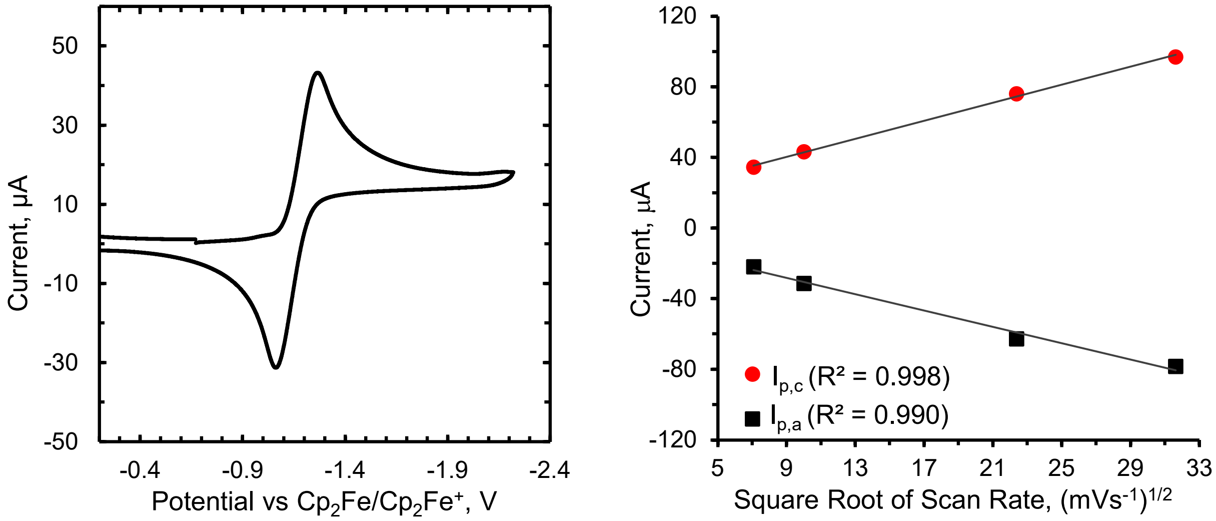

Figure S49. (a) Cyclic voltammogram of **15** in 0.1 M $[^{n}Bu_4N]^+[PF_6]^-/CH_2Cl_2$ vs. external $Cp_2Fe^{0/+}$ at 22°C (scan rate = 100 mV/s). (b) Randles-Sevcik graph of peak currents versus square root of the scan rate for **15** in 0.1 M $[^{n}Bu_4N]^+[PF_6]^-/CH_2Cl_2$.

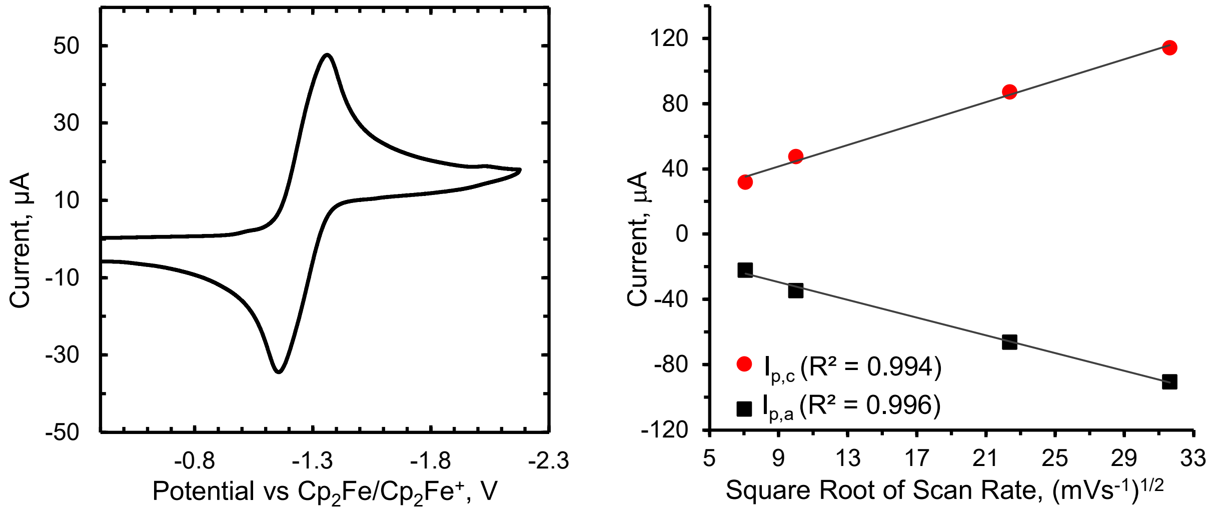

Figure S50. (a) Cyclic voltammogram of **16** in 0.1 M $[^{n}Bu_4N]^+[PF_6]^-/CH_2Cl_2$ vs. external $Cp_2Fe^{0/+}$ at 22°C (scan rate = 100 mV/s). (b) Randles-Sevcik graph of peak currents versus square root of the scan rate for **16** in 0.1 M $[^{n}Bu_4N]^+[PF_6]^-/CH_2Cl_2$.

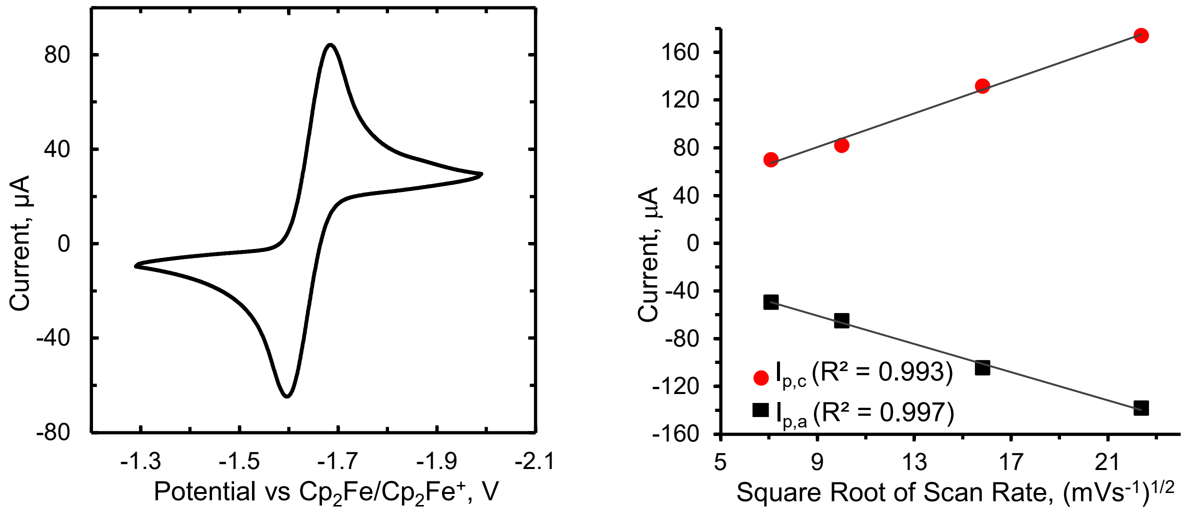

Figure S51. (a) Cyclic voltammogram of 2,2’-diamino-1,1’,3,3’-tetraethoxycarbonyl-6,6’-biazulene in 0.1 M $[^nBu_4N]^+[PF_6]^-/CH_2Cl_2$ vs. external $Cp_2Fe^{0/+}$ at 22°C (scan rate = 100 mV/s). (b) Randles-Sevcik graph of peak currents versus square root of the scan rate for 2,2’-diamino-1,1’,3,3’-tetraethoxycarbonyl-6,6’-biazulene in 0.1 M $[^nBu_4N]^+[PF_6]^-/CH_2Cl_2$.

Table S4. Cyclic voltametric data pertaining to the two-electron reduction of 2,2'-functionalized 6,6'-biazulenes.

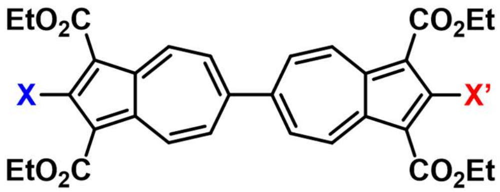

**Compound**	**X**	**X'**	**$E_{1/2}$, V**	**$\Delta E_{p,c-p,a}$, mV**	**$i_{p,c}/i_{p,a}$**
**1**[1]	Cl	Cl	−1.19	72	1.22
**3**	SH	SH	−1.31	75	0.99
**4**	SMe	SMe	−1.30	121	1.07
**5**	$SAuPPh_3$	$SAuPPh_3$	−1.48	143	1.05
**6**	NMe_2	NMe_2	−1.59	82	1.00
**11**	Cl	SH	−1.25	59	0.99
**12**	Cl	SMe	−1.24	73	1.04
**15**	SH	$NC\text{-}Cr(CO)_5$	−1.16	206	0.97
**16**	$SAuPPh_3$	$NC\text{-}Cr(CO)_5$	−1.26	211	1.07
**17**[7]	$NC\text{-}Cr(CO)_5$	$NC\text{-}Cr(CO)_5$	−1.00	203	0.99
**18**[7]	H	$NC\text{-}Cr(CO)_5$	−1.14	150	1.11
	NH_2	NH_2	−1.64	88	0.99

E. Electronic Absorption Spectra

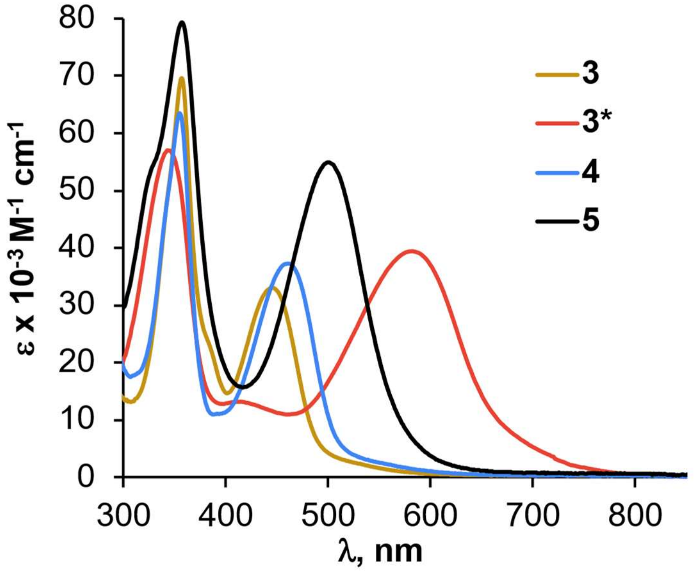

Figure S52. Electronic absorption spectra of **3**, **3***, **4**, and **5** in CH_2Cl_2 at 22°C.

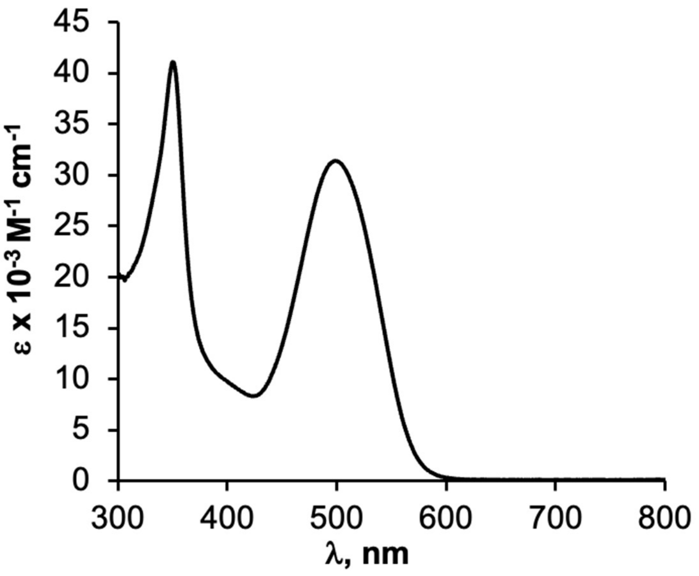

Figure S53. Electronic absorption spectrum of **6** in CH_2Cl_2 at 22°C.

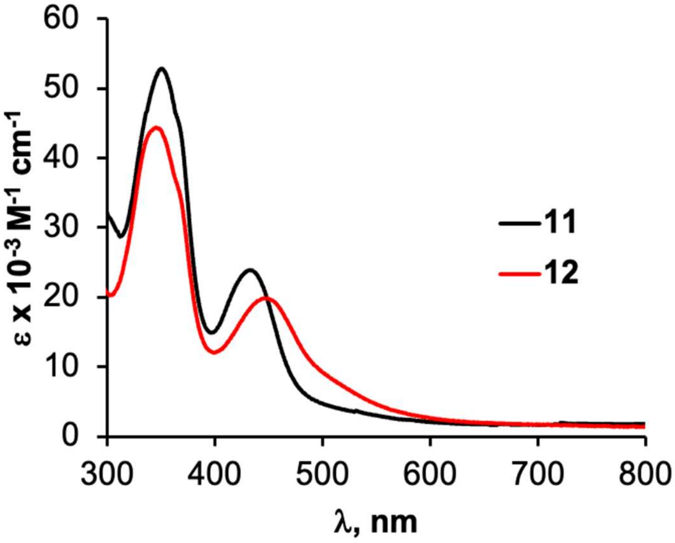

Figure S54. Electronic absorption spectra of **11** and **12** in CH_2Cl_2 at 22°C.

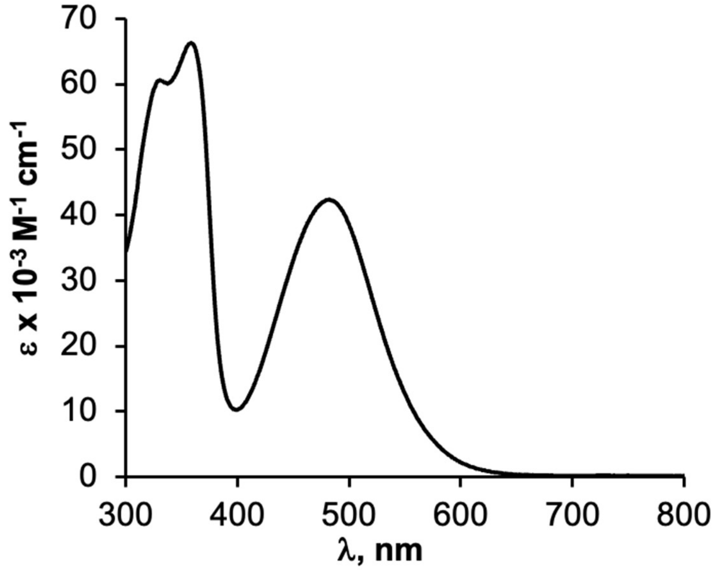

Figure S55. Electronic absorption spectrum of **14** in CH_2Cl_2 at 22°C.

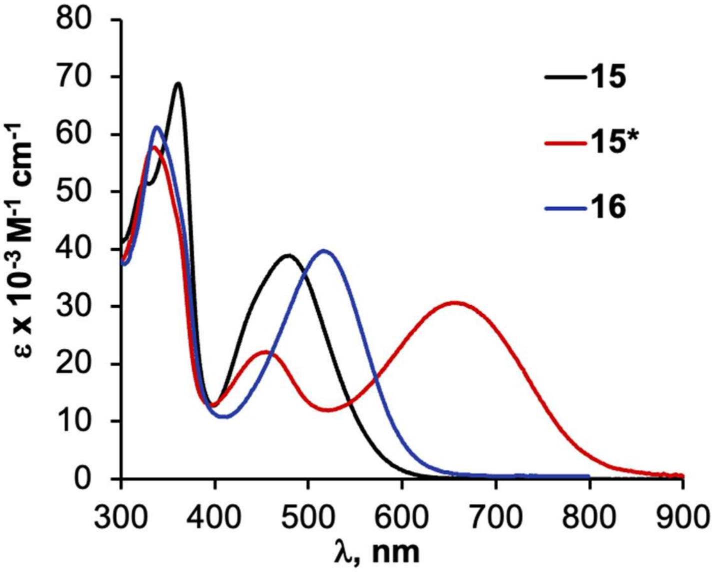

Figure S56. Electronic absorption spectra of **15**, **15***, and **16** in CH_2Cl_2 at 22°C.

F. X-ray Crystallographic Studies

F1. Experimental and Refinement Model Description

Table S4 contains crystal data, collection parameters, and refinement criteria for the crystal structure of $C_{34}H_{34}O_8S_2$ (**4**). Crystals of **4** were grown by slow evaporation of the solvent from a solution of **4** in $CHCl_3$ at 4°C. A red plate crystal was selected and mounted on a Bruker APEX-II CCD diffractometer. The crystal was kept at 100 K during data collection. The intensity data were corrected for absorption using SADABS-2016/2. Using Olex2[8], the structure was solved with the olex2.solve[9] structure solution program using Charge Flipping and refined with the SHELXL[10] refinement package using Least Squares minimization.

A direct-methods solution identified most of the non-hydrogen atoms from the E-map. Full-matrix least-squares/difference Fourier cycles were performed that located the remaining nonhydrogen atoms. All non-hydrogen atoms were refined with anisotropic displacement parameters. All of the hydrogen atoms were placed in calculated positions and refined in the riding-model approximation. CCDC entry 2482367 contains the relevant crystallographic data associated with this article. These data can be accessed free of charge from the Cambridge Crystallographic Data Centre.

The structure consists of a single entire molecule in the asymmetric unit. One half of the molecule (all atom labels with primes) did not indicate any disorder. However, disorder was modeled to resolve different conformations pertaining to the azulenic five membered carbon ring and the associated SMe and CO_2Et substituents. The disorder model portion at 50% occupancy was fixed, while the other two disorder components were freely refined for occupancy. Disorder component details are as follows:

50% occupancy C17, S1, C1-C3, C11-13, O1, O2
31% occupancy C17A, S1A, C1A-C3A, C11A-C13A, O1A, O2A
19% occupancy C17B, S1BA C1B-C3B, C11B-C13B, O1B, O2B

The disorder component involving the SMe group (S1-C17) is oriented *anti* to the S1’-C17’ group, while the other two disorder conformations (S1A-C17A and S1B-C17B with the 31% and 19% occupancies, respectively) are syn with respect to the S1’-C17’ fragment.

To achieve a reasonably accurate disorder model, several paired thermal parameters were constrained to be equivalent (EADP), numerous bond lengths in adjacent disorder conformations were restrained to be similar (SADI), and bond angles and distances within similar conformations of the ethyl ester groups were restrained to be equal (SAME). Complete details of the restraints and constraints implemented are listed below:

Details:
1. Fixed Uiso
 At 1.2 times of:
  All C(H) groups, All C(H,H) groups
 At 1.5 times of:
  All C(H,H,H) groups
2. Restrained distances
 C1-C14 ≈ C1A-C14 ≈ C1B-C14
 with sigma of 0.02
 C10-C3 ≈ C10-C3A ≈ C10-C3B
 with sigma of 0.0025
 C14-O4 ≈ C14-O4A
 with sigma of 0.02
 O4-C15 ≈ O4A-C15A
 with sigma of 0.02
 C15-C16 ≈ C15A-C16A
 with sigma of 0.02
 C14-C15 ≈ C14-C15A
 with sigma of 0.04
 O4-C16 ≈ O4A-C16A
 with sigma of 0.04
3. Uiso/Uaniso restraints and constraints
All non-hydrogen atoms have similar U: within 1.2A with sigma of 0.01 and sigma for terminal atoms of 0.02 within 1.2A
Uanis(C1) ≈ Ueq, Uanis(C1A) ≈ Ueq, Uanis(C1B) ≈ Ueq: with sigma of 0.005 and sigma for terminal atoms of 0.002
Uanis(C1) = Uanis(C1A) = Uanis(C1B)
Uanis(C3) = Uanis(C3A) = Uanis(C3B)
4. Same fragment restrains
{C17A, S1A, C1A, C2A, C3A, C11A, O1A, O2A, C12A, C13A} sigma for 1-2: 0.02, 1-3: 0.04
{C17B, S1B, C1B, C2B, C3B, C11B, O1B, O2B, C12B, C13B} sigma for 1-2: 0.02, 1-3: 0.04
as in
{C17, S1, C1, C2, C3, C11, O1, O2, C12, C13}
5. Others

1*[Sof(C17A)+Sof(H17G)+Sof(H17H)+Sof(H17I)+Sof(S1A)+Sof(C1A)+Sof(C2A)+
Sof(C3A)+Sof(C11A)+Sof(O1A)+Sof(O2A)+Sof(C12A)+Sof(H12E)+Sof(H12F)+Sof(C13A)+
Sof(H13G)+Sof(H13H)+Sof(H13I)]+1*[Sof(C17B)+Sof(H17J)+Sof(H17K)+Sof(H17L)+
Sof(S1B)+Sof(C1B)+Sof(C2B)+Sof(C3B)+Sof(C11B)+Sof(O1B)+Sof(O2B)+Sof(C12B)+
Sof(H12G)+Sof(H12H)+Sof(C13B)+Sof(H13J)+Sof(H13K)+Sof(H13L)]=0.5 with esd of
0.0001
Sof(C17A)=Sof(H17G)=Sof(H17H)=Sof(H17I)=Sof(S1A)=Sof(C1A)=Sof(C2A)=Sof(C3A)=

Sof(C11A)=Sof(O1A)=Sof(O2A)=Sof(C12A)=Sof(H12E)=Sof(H12F)=Sof(C13A)=Sof(H13G)
=
Sof(H13H)=Sof(H13I)=FVAR(1)
Sof(C17B)=Sof(H17J)=Sof(H17K)=Sof(H17L)=Sof(S1B)=Sof(C1B)=Sof(C2B)=Sof(C3B)=
Sof(C11B)=Sof(O1B)=Sof(O2B)=Sof(C12B)=Sof(H12G)=Sof(H12H)=Sof(C13B)=Sof(H13J)=
Sof(H13K)=Sof(H13L)=FVAR(2)
Fixed Sof: C17(0.5) H17D(0.5) H17E(0.5) H17F(0.5) S1(0.5) C1(0.5) C2(0.5)
C3(0.5) C11(0.5) O1(0.5) O2(0.5) C12(0.5) H12C(0.5) H12D(0.5) C13(0.5)
H13D(0.5) H13E(0.5) H13F(0.5) O4(0.5) C15(0.5) H15C(0.5) H15D(0.5) C16(0.5)
H16D(0.5) H16E(0.5) H16F(0.5) O4A(0.5) C15A(0.5) H15E(0.5) H15F(0.5) C16A(0.5)
H16G(0.5) H16H(0.5) H16I(0.5)
6.a Secondary CH2 refined with riding coordinates:
C12'(H12A,H12B), C15'(H15A,H15B), C12(H12C,H12D), C12A(H12E,H12F), C12B(H12G,
H12H), C15(H15C,H15D), C15A(H15E,H15F)
6.b Aromatic/amide H refined with riding coordinates:
C4(H4), C4'(H4'), C5(H5), C5'(H5'), C7(H7), C7'(H7'), C8(H8), C8'(H8')
6.c Idealised Me refined as rotating group:
C13'(H13A,H13B,H13C), C16'(H16A,H16B,H16C), C17'(H17A,H17B,H17C), C17(H17D,
H17E,H17F), C13(H13D,H13E,H13F), C17A(H17G,H17H,H17I), C13A(H13G,H13H,H13I),
C17B(H17J,H17K,H17L), C13B(H13J,H13K,H13L), C16(H16D,H16E,H16F), C16A(H16G,
H16H,H16I)

Table S5. Crystal data and structure refinement for **4**.

Empirical formula	$C_{34}H_{34}O_8S_2$
Formula weight	634.73
Temperature/K	100.15
Crystal system	monoclinic
Space group	$P2_1/c$
a/Å	17.2376(8)
b/Å	11.8058(5)
c/Å	15.3135(7)
α/°	90
β/°	92.212(2)
γ/°	90
Volume/Å^3	3114.0(2)
Z	4
ρ_{calc}g/cm^3	1.354
μ/mm^{-1}	0.223
F(000)	1336.0
Crystal size/mm^3	0.4 × 0.2 × 0.1
Radiation	MoKα (λ = 0.71073)
2Θ range for data collection/°	4.182 to 50.7
Index ranges	$-20 \leq h \leq 20$, $-14 \leq k \leq 14$, $-18 \leq l \leq 18$
Reflections collected	96802
Independent reflections	5697 [R_{int} = 0.0521, R_{sigma} = 0.0186]
Data/restraints/parameters	5697/279/593
Goodness-of-fit on F^2	1.056
Final R indexes [I>=2σ (I)]	R_1 = 0.0592, wR_2 = 0.1547
Final R indexes [all data]	R_1 = 0.0693, wR_2 = 0.1641
Largest diff. peak/hole / e Å^{-3}	1.59/-0.48

Table S6. Fractional Atomic Coordinates (×10^4) and Equivalent Isotropic Displacement Parameters (Å^2×10^3) for **4**. U_{eq} is defined as 1/3 of the trace of the orthogonalised U_{IJ} tensor.

**Atom**	***x***	***y***	***z***	**U(eq)**
S1'	3400.7(4)	3220.0(6)	7595.7(4)	30.2(2)
O1'	5662.5(11)	4981.4(16)	7543.1(12)	30.7(4)
O2'	4428.6(12)	5059.3(18)	7934.9(14)	39.2(5)
O3	7688.2(11)	233.0(17)	1197.8(13)	32.7(5)
O3'	2471.4(11)	2815(2)	5702.4(14)	37.9(5)
O4'	3187.4(11)	1580.7(18)	4958.4(13)	34.5(5)
C1'	3841.8(15)	2930(2)	5833.3(17)	22.2(5)
C2'	3962.6(15)	3391(2)	6681.4(17)	24.3(5)
C3'	4680.6(15)	3980(2)	6715.7(17)	23.4(5)

C4	7760.4(16)	4358(2)	2936.4(18)	28.0(6)
C4'	5784.5(15)	4179(2)	5718.3(16)	22.9(5)
C5	7125.8(15)	4234(2)	3456.8(17)	25.6(6)
C5'	6207.1(15)	3976(2)	4981.0(16)	23.2(5)
C6	6623.0(15)	3309(2)	3547.8(16)	23.0(5)
C6'	5998.1(15)	3432(2)	4198.1(16)	21.9(5)
C7	6663.6(15)	2275(2)	3104.7(17)	24.6(5)
C7'	5261.7(15)	2983(2)	3976.0(16)	22.8(5)
C8	7163.0(15)	1907(2)	2475.1(17)	24.5(5)
C8'	4612.2(15)	2864(2)	4472.3(16)	21.4(5)
C9	7783.5(15)	2473(2)	2104.3(17)	26.2(6)
C9'	4505.3(14)	3155(2)	5346.3(16)	21.3(5)
C10	8072.6(17)	3605(2)	2340.8(19)	31.1(6)
C10'	5044.6(14)	3813(2)	5913.6(16)	21.2(5)
C11'	4993.3(16)	4699(2)	7428.8(17)	26.1(6)
C12'	4656(2)	5831(3)	8643(2)	44.2(8)
C13'	3992(3)	5949(4)	9212(3)	65.5(11)
C14	8203.8(15)	904(2)	1084.0(17)	27.4(6)
C14'	3098.9(16)	2450(2)	5504.1(17)	26.8(6)
C15'	2468.5(19)	1031(3)	4641(2)	45.6(8)
C16'	2214(3)	176(4)	5271(3)	73.5(13)
C17'	2927.1(18)	1870(3)	7443(2)	36.9(7)
C17	10513(4)	3362(9)	1239(7)	86(4)
S1	9744.4(10)	2469.5(15)	779.0(12)	44.6(4)
C1	8304(8)	1998(8)	1513(8)	25.7(15)
C2	8929(5)	2762(7)	1394(6)	29(2)
C3	8792(3)	3727(6)	1921(5)	24.0(15)
C11	9198(8)	4833(8)	1870(13)	35(2)
O1	9358(3)	5313(5)	1224(3)	55.4(13)
O2	9391(2)	5227(3)	2687(3)	34.3(9)
C12	9713(4)	6368(5)	2744(5)	41.1(15)
C13	9083(4)	7224(5)	2818(5)	40.9(14)
C17A	8856(12)	2525(13)	-566(10)	29(3)
S1A	9400(7)	3142(10)	356(8)	26.4(14)
C1A	8254(17)	2113(13)	1378(19)	25.7(15)
C2A	8758(15)	3038(17)	1213(13)	23(3)
C3A	8649(9)	3972(13)	1762(9)	24.0(15)
C11A	9082(11)	5052(15)	1840(20)	33(3)
O1A	8819(14)	5939(14)	2026(17)	33(4)
O2A	9841(6)	4858(8)	1671(6)	35(2)
C12A	10322(6)	5872(9)	1619(10)	46(3)
C13A	11082(6)	5519(11)	1282(10)	62(4)
C17B	8750(20)	2150(20)	-584(18)	40(7)

S1B	9306(12)	2920(18)	253(13)	30(3)
C1B	8170(20)	2070(17)	1410(30)	25.7(15)
C2B	8700(30)	2900(30)	1150(20)	25(4)
C3B	8614(15)	3860(19)	1689(14)	24.0(15)
C11B	9085(18)	4910(20)	1710(40)	34(4)
O1B	8850(20)	5830(20)	1850(30)	24(5)
O2B	9770(9)	4680(12)	1324(10)	30(3)
C12B	10192(9)	5672(14)	1048(12)	43(4)
C13B	10704(14)	6060(20)	1780(16)	66(6)
O4	8721(6)	733(9)	462(6)	39(2)
C15	8733(7)	-367(9)	30(6)	43(2)
C16	9117(7)	-1222(11)	618(8)	61(3)
O4A	8871(6)	614(10)	733(6)	40(2)
C15A	8884(7)	-542(9)	392(7)	46(3)
C16A	9115(6)	-1346(9)	1116(8)	61(3)

Table S7. Anisotropic Displacement Parameters ($Å^2 \times 10^3$) for **4**. The Anisotropic displacement factor exponent takes the form: $-2\pi^2[h^2a^{*2}U_{11}+2hka^*b^*U_{12}+\ldots]$.

Atom	U_{11}	U_{22}	U_{33}	U_{23}	U_{13}	U_{12}
S1'	29.9(4)	34.3(4)	27.2(4)	5.6(3)	10.5(3)	2.7(3)
O1'	31.9(11)	32.2(10)	28.0(10)	-5.1(8)	3.2(8)	0.0(8)
O2'	37.1(12)	41.3(12)	40.2(12)	-18.5(10)	14.5(9)	-8.3(9)
O3	29.3(10)	34.5(11)	34.8(11)	-5.5(9)	5.6(8)	-2.7(9)
O3'	22.2(10)	54.3(13)	37.4(11)	7.9(10)	1.7(8)	4.2(9)
O4'	29.6(11)	39.5(12)	34.4(11)	-1.9(9)	2.8(8)	-11.5(9)
C1'	21.2(12)	21.7(12)	23.8(12)	4.6(10)	2.4(10)	3.2(10)
C2'	24.2(13)	23.3(13)	25.6(13)	4.9(10)	5.3(10)	6.1(10)
C3'	24.9(13)	22.7(12)	22.9(13)	2.0(10)	4.3(10)	3.4(10)
C4	27.8(14)	26.7(14)	30.0(14)	-0.9(11)	5.5(11)	-5.4(11)
C4'	24.2(13)	21.9(12)	22.5(12)	0.4(10)	-0.1(10)	0.8(10)
C5	25.8(13)	25.1(13)	26.1(13)	0.4(10)	4.1(10)	0.6(11)
C5'	20.7(12)	25.1(13)	23.8(13)	1.0(10)	1.3(10)	-1.2(10)
C6	21.5(13)	26.9(13)	20.7(12)	2.7(10)	1.9(10)	1.8(10)
C6'	23.3(13)	21.2(12)	21.4(12)	4.8(10)	3.3(10)	1.5(10)
C7	22.1(13)	27.8(13)	24.2(13)	4.5(11)	2.5(10)	-2.0(10)
C7'	25.5(13)	23.8(13)	19.0(12)	2.4(10)	0.1(10)	1.6(10)
C8	23.3(13)	25.0(13)	25.2(13)	0.0(10)	0.5(10)	-0.4(10)
C8'	20.5(12)	21.6(12)	22.0(12)	3.1(10)	-1.2(10)	0.1(10)
C9	24.7(13)	30.5(14)	23.6(13)	-0.4(11)	2.8(10)	0.3(11)
C9'	21.0(12)	18.9(12)	23.9(12)	5.1(10)	1.2(10)	2.2(10)
C10	31.6(15)	31.2(15)	31.0(14)	-2.4(12)	9.1(12)	-7.1(12)

C10'	23.6(13)	18.7(12)	21.1(12)	2.6(9)	0.9(10)	3.6(10)
C11'	30.5(15)	24.4(13)	23.8(13)	0.9(10)	6.8(11)	2.8(11)
C12'	52(2)	38.3(17)	43.5(18)	-18.0(14)	14.3(15)	-15.6(15)
C13'	68(3)	59(2)	70(3)	-25(2)	15(2)	-11(2)
C14	23.2(13)	34.8(15)	24.4(13)	-2.1(11)	1.7(10)	3.3(11)
C14'	27.3(14)	30.3(14)	22.9(13)	8.7(11)	1.8(10)	-1.1(11)
C15'	38.6(18)	57(2)	40.6(18)	-2.8(15)	-0.3(14)	-22.6(16)
C16'	64(3)	79(3)	78(3)	0(2)	11(2)	-27(2)
C17'	33.5(16)	40.0(17)	37.3(16)	13.8(13)	1.2(13)	-7.1(13)
C17	35(4)	105(7)	120(8)	-69(7)	39(5)	-27(4)
S1	34.7(8)	48.7(10)	52.2(10)	-21.7(8)	23.8(8)	-10.5(8)
C1	25(2)	30.5(17)	22(3)	0.5(17)	2(2)	-0.6(16)
C2	27(4)	34(4)	27(4)	-5(3)	7(3)	-2(3)
C3	26(2)	25(3)	21(3)	4(2)	2(2)	0(2)
C11	32(4)	32(4)	43(5)	-10(4)	22(4)	-4(3)
O1	61(3)	58(3)	48(3)	-12(3)	23(3)	-29(3)
O2	28(2)	33(2)	42(2)	-11.7(18)	6.9(17)	-7.5(17)
C12	33(3)	37(3)	53(4)	-12(3)	7(3)	-12(3)
C13	41(4)	33(3)	49(4)	-3(3)	2(3)	-1(3)
C17A	34(7)	25(9)	29(5)	1(5)	9(4)	3(6)
S1A	25(3)	25(4)	29(3)	1(2)	10(2)	2(2)
C1A	25(2)	30.5(17)	22(3)	0.5(17)	2(2)	-0.6(16)
C2A	17(5)	28(6)	22(5)	14(4)	0(5)	2(4)
C3A	26(2)	25(3)	21(3)	4(2)	2(2)	0(2)
C11A	31(5)	30(5)	40(7)	3(5)	16(5)	-8(5)
O1A	37(5)	23(5)	39(11)	-4(4)	19(5)	-2(4)
O2A	26(4)	28(4)	53(6)	-14(4)	13(4)	-6(3)
C12A	34(6)	39(6)	66(8)	-19(6)	10(7)	-12(5)
C13A	30(6)	53(7)	102(11)	-12(7)	17(6)	-10(5)
C17B	48(13)	35(17)	36(9)	5(10)	1(9)	4(12)
S1B	27(5)	34(7)	31(5)	1(4)	12(4)	1(4)
C1B	25(2)	30.5(17)	22(3)	0.5(17)	2(2)	-0.6(16)
C2B	22(7)	31(7)	21(7)	0(6)	2(6)	13(6)
C3B	26(2)	25(3)	21(3)	4(2)	2(2)	0(2)
C11B	33(6)	31(6)	38(8)	1(6)	19(6)	0(6)
O1B	28(7)	18(8)	28(11)	-1(6)	19(6)	8(6)
O2B	23(5)	24(6)	44(8)	-3(6)	18(6)	1(5)
C12B	35(7)	36(7)	59(9)	-11(8)	18(8)	-9(6)
C13B	41(12)	67(13)	90(14)	-35(11)	2(12)	-10(11)
O4	39(5)	35(4)	44(5)	-16(3)	20(4)	-11(3)
C15	52(6)	34(4)	45(6)	-14(4)	22(5)	-3(4)
C16	47(5)	52(6)	85(7)	-15(7)	9(6)	15(4)
O4A	23(3)	36(3)	62(6)	-16(4)	9(4)	5(2)

C15A	38(5)	35(5)	67(7)	-16(6)	14(5)	2(4)
C16A	44(5)	39(4)	99(8)	-6(6)	14(6)	5(3)

Table S8. Bond Lengths for **4**.

Atom	Atom	Length/Å	Atom	Atom	Length/Å
S1'	C2'	1.745(3)	C14	C1	1.456(8)
S1'	C17'	1.801(3)	C14	C1A	1.498(12)
O1'	C11'	1.207(3)	C14	C1B	1.466(15)
O2'	C11'	1.337(3)	C14	O4	1.344(9)
O2'	C12'	1.459(4)	C14	O4A	1.333(9)
O3	C14	1.208(3)	C15'	C16'	1.476(6)
O3'	C14'	1.214(3)	C17	S1	1.814(7)
O4'	C14'	1.336(3)	S1	C2	1.756(7)
O4'	C15'	1.465(4)	C1	C2	1.422(9)
C1'	C2'	1.416(4)	C2	C3	1.421(9)
C1'	C9'	1.414(3)	C3	C11	1.485(9)
C1'	C14'	1.471(4)	C11	O1	1.181(19)
C2'	C3'	1.419(4)	C11	O2	1.364(19)
C3'	C10'	1.415(4)	O2	C12	1.458(7)
C3'	C11'	1.469(4)	C12	C13	1.490(8)
C4	C5	1.386(4)	C17A	S1A	1.816(12)
C4	C10	1.397(4)	S1A	C2A	1.753(11)
C4'	C5'	1.388(4)	C1A	C2A	1.423(12)
C4'	C10'	1.390(4)	C2A	C3A	1.403(12)
C5	C6	1.405(4)	C3A	C11A	1.479(12)
C5'	C6'	1.395(4)	C11A	O1A	1.181(19)
C6	C6'	1.502(3)	C11A	O2A	1.36(2)
C6	C7	1.399(4)	O2A	C12A	1.460(11)
C6'	C7'	1.405(4)	C12A	C13A	1.484(12)
C7	C8	1.387(4)	C17B	S1B	1.820(15)
C7'	C8'	1.384(4)	S1B	C2B	1.754(13)
C8	C9	1.400(4)	C1B	C2B	1.413(15)
C8'	C9'	1.401(4)	C2B	C3B	1.414(14)
C9	C10	1.466(4)	C3B	C11B	1.478(14)
C9	C1	1.415(12)	C11B	O1B	1.18(2)
C9	C1A	1.465(19)	C11B	O2B	1.37(2)
C9	C1B	1.36(3)	O2B	C12B	1.451(14)
C9'	C10'	1.470(4)	C12B	C13B	1.474(16)
C10	C3	1.426(4)	O4	C15	1.458(10)
C10	C3A	1.425(4)	C15	C16	1.490(13)
C10	C3B	1.425(4)	O4A	C15A	1.462(10)

Table S9. Bond Angles for **4**.

Atom	Atom	Atom	Angle/°	Atom	Atom	Atom	Angle/°
C2'	S1'	C17'	105.12(14)	O4	C14	C1	112.5(7)
C11'	O2'	C12'	116.7(2)	O4A	C14	C1A	109.0(10)
C14'	O4'	C15'	115.6(2)	O3'	C14'	O4'	123.6(3)
C2'	C1'	C14'	123.9(2)	O3'	C14'	C1'	123.4(3)
C9'	C1'	C2'	108.6(2)	O4'	C14'	C1'	113.0(2)
C9'	C1'	C14'	127.0(2)	O4'	C15'	C16'	110.8(3)
C1'	C2'	S1'	128.7(2)	C2	S1	C17	105.4(4)
C1'	C2'	C3'	108.6(2)	C9	C1	C14	125.0(8)
C3'	C2'	S1'	122.5(2)	C9	C1	C2	109.4(6)
C2'	C3'	C11'	127.3(2)	C2	C1	C14	125.6(9)
C10'	C3'	C2'	108.4(2)	C1	C2	S1	124.7(7)
C10'	C3'	C11'	124.3(2)	C3	C2	S1	128.1(6)
C5	C4	C10	129.8(3)	C3	C2	C1	107.0(6)
C5'	C4'	C10'	129.5(2)	C10	C3	C11	122.2(9)
C4	C5	C6	130.0(3)	C2	C3	C10	110.0(5)
C4'	C5'	C6'	130.7(2)	C2	C3	C11	125.8(9)
C5	C6	C6'	116.8(2)	O1	C11	C3	126.2(13)
C7	C6	C5	126.0(2)	O1	C11	O2	123.3(7)
C7	C6	C6'	117.2(2)	O2	C11	C3	110.5(13)
C5'	C6'	C6	116.5(2)	C11	O2	C12	116.7(7)
C5'	C6'	C7'	125.6(2)	O2	C12	C13	110.7(5)
C7'	C6'	C6	117.9(2)	C2A	S1A	C17A	103.4(8)
C8	C7	C6	130.9(2)	C9	C1A	C14	118.5(11)
C8'	C7'	C6'	130.4(2)	C2A	C1A	C9	105.8(9)
C7	C8	C9	129.5(3)	C2A	C1A	C14	134.9(16)
C7'	C8'	C9'	129.7(2)	C1A	C2A	S1A	126.4(12)
C8	C9	C10	126.3(2)	C3A	C2A	S1A	119.7(9)
C8	C9	C1	125.7(4)	C3A	C2A	C1A	113.6(11)
C8	C9	C1A	128.6(6)	C10	C3A	C11A	125.1(15)
C1	C9	C10	107.5(4)	C2A	C3A	C10	104.2(9)
C1A	C9	C10	105.0(5)	C2A	C3A	C11A	130.2(13)
C1B	C9	C8	123.6(9)	O1A	C11A	C3A	125.9(18)
C1B	C9	C10	109.8(8)	O1A	C11A	O2A	125.1(14)
C1'	C9'	C10'	107.1(2)	O2A	C11A	C3A	109.0(14)
C8'	C9'	C1'	126.6(2)	C11A	O2A	C12A	115.1(10)
C8'	C9'	C10'	126.2(2)	O2A	C12A	C13A	107.4(9)
C4	C10	C9	127.3(2)	C2B	S1B	C17B	103.4(13)
C4	C10	C3	126.4(4)	C9	C1B	C14	128.4(19)
C4	C10	C3A	120.5(6)	C9	C1B	C2B	108.7(12)
C4	C10	C3B	127.0(9)	C2B	C1B	C14	121.4(19)

C3	C10	C9	105.9(4)	C1B	C2B	S1B	129.9(17)
C3A	C10	C9	111.2(6)	C1B	C2B	C3B	107.7(14)
C3B	C10	C9	104.2(8)	C3B	C2B	S1B	121.9(14)
C3'	C10'	C9'	107.2(2)	C10	C3B	C11B	122.4(18)
C4'	C10'	C3'	125.8(2)	C2B	C3B	C10	109.2(13)
C4'	C10'	C9'	127.0(2)	C2B	C3B	C11B	127.6(15)
O1'	C11'	O2'	122.7(2)	O1B	C11B	C3B	125(2)
O1'	C11'	C3'	126.1(2)	O1B	C11B	O2B	125(2)
O2'	C11'	C3'	111.1(2)	O2B	C11B	C3B	108.1(17)
O2'	C12'	C13'	107.8(3)	C11B	O2B	C12B	114.8(14)
O3	C14	C1	126.3(5)	O2B	C12B	C13B	108.9(16)
O3	C14	C1A	128.0(9)	C14	O4	C15	118.5(9)
O3	C14	C1B	121.8(11)	O4	C15	C16	110.1(8)
O3	C14	O4	121.0(5)	C14	O4A	C15A	114.2(9)
O3	C14	O4A	122.7(6)	O4A	C15A	C16A	109.5(9)

Table S10. Torsion Angles for **4**.

A	B	C	D	Angle/°	A	B	C	D	Angle/°
S1'	C2'	C3'	C10'	170.81(18)	C10	C9	C1	C14	-176.5(9)
S1'	C2'	C3'	C11'	-12.2(4)	C10	C9	C1	C2	2.5(11)
O3	C14	C1	C9	-3.5(15)	C10	C9	C1A	C14	169(2)
O3	C14	C1	C2	177.7(8)	C10	C9	C1A	C2A	-2(3)
O3	C14	C1A	C9	21(4)	C10	C9	C1B	C14	165(4)
O3	C14	C1A	C2A	-171(3)	C10	C9	C1B	C2B	-1(5)
O3	C14	C1B	C9	27(6)	C10	C3	C11	O1	-120.5(16)
O3	C14	C1B	C2B	-169(4)	C10	C3	C11	O2	61.2(12)
O3	C14	O4	C15	-10.6(10)	C10	C3A	C11A	O1A	-40(4)
O3	C14	O4A	C15A	7.7(10)	C10	C3A	C11A	O2A	138(2)
C1'	C2'	C3'	C10'	-4.8(3)	C10	C3B	C11B	O1B	-45(7)
C1'	C2'	C3'	C11'	172.1(2)	C10	C3B	C11B	O2B	150(3)
C1'	C9'	C10'	C3'	-1.1(3)	C10'	C3'	C11'	O1'	-21.5(4)
C1'	C9'	C10'	C4'	177.1(2)	C10'	C3'	C11'	O2'	155.6(2)
C2'	C1'	C9'	C8'	-178.5(2)	C10'	C4'	C5'	C6'	5.9(5)
C2'	C1'	C9'	C10'	-1.8(3)	C11'	O2'	C12'	C13'	-169.4(3)
C2'	C1'	C14'	O3'	34.5(4)	C11'	C3'	C10'	C4'	8.4(4)
C2'	C1'	C14'	O4'	-146.2(2)	C11'	C3'	C10'	C9'	-173.4(2)
C2'	C3'	C10'	C4'	-174.6(2)	C12'	O2'	C11'	O1'	0.4(4)
C2'	C3'	C10'	C9'	3.6(3)	C12'	O2'	C11'	C3'	-176.9(2)
C2'	C3'	C11'	O1'	162.0(3)	C14	C1	C2	S1	-6.0(17)
C2'	C3'	C11'	O2'	-20.9(4)	C14	C1	C2	C3	178.9(10)
C4	C5	C6	C6'	178.0(3)	C14	C1A	C2A	S1A	17(6)
C4	C5	C6	C7	-0.3(5)	C14	C1A	C2A	C3A	-170(3)

C4	C10	C3	C2	177.1(5)	C14	C1B	C2B	S1B	18(8)
C4	C10	C3	C11	-18.0(11)	C14	C1B	C2B	C3B	-169(4)
C4	C10	C3A	C2A	-174.0(14)	C14	O4	C15	C16	-76.1(12)
C4	C10	C3A	C11A	13(2)	C14	O4A	C15A	C16A	-85.7(10)
C4	C10	C3B	C2B	-172(3)	C14'	O4'	C15'	C16'	-84.5(4)
C4	C10	C3B	C11B	17(4)	C14'	C1'	C2'	S1'	16.7(4)
C4'	C5'	C6'	C6	-176.9(3)	C14'	C1'	C2'	C3'	-168.0(2)
C4'	C5'	C6'	C7'	2.2(5)	C14'	C1'	C9'	C8'	-6.7(4)
C5	C4	C10	C9	1.4(5)	C14'	C1'	C9'	C10'	170.0(2)
C5	C4	C10	C3	-170.4(4)	C15'	O4'	C14'	O3'	-3.5(4)
C5	C4	C10	C3A	169.2(10)	C15'	O4'	C14'	C1'	177.2(2)
C5	C4	C10	C3B	165.1(18)	C17'	S1'	C2'	C1'	26.9(3)
C5	C6	C6'	C5'	-39.7(3)	C17'	S1'	C2'	C3'	-147.8(2)
C5	C6	C6'	C7'	141.0(2)	C17	S1	C2	C1	-154.2(10)
C5	C6	C7	C8	-2.0(5)	C17	S1	C2	C3	19.8(10)
C5'	C4'	C10'	C3'	174.3(3)	S1	C2	C3	C10	-177.3(6)
C5'	C4'	C10'	C9'	-3.5(4)	S1	C2	C3	C11	18.4(14)
C5'	C6'	C7'	C8'	-7.0(4)	C1	C9	C10	C4	-177.0(7)
C6	C6'	C7'	C8'	172.1(2)	C1	C9	C10	C3	-3.9(7)
C6	C7	C8	C9	0.7(5)	C1	C14	O4	C15	174.7(9)
C6'	C6	C7	C8	179.7(3)	C1	C2	C3	C10	-2.5(10)
C6'	C7'	C8'	C9'	-0.3(5)	C1	C2	C3	C11	-166.7(10)
C7	C6	C6'	C5'	138.7(2)	C2	C3	C11	O1	42.0(18)
C7	C6	C6'	C7'	-40.5(3)	C2	C3	C11	O2	-136.3(11)
C7	C8	C9	C10	3.1(5)	C3	C11	O2	C12	-173.1(7)
C7	C8	C9	C1	174.6(7)	C11	O2	C12	C13	89.3(10)
C7	C8	C9	C1A	-173.2(19)	O1	C11	O2	C12	8.5(18)
C7	C8	C9	C1B	-171(3)	C17A	S1A	C2A	C1A	39(3)
C7'	C8'	C9'	C1'	-174.9(3)	C17A	S1A	C2A	C3A	-134(2)
C7'	C8'	C9'	C10'	9.1(4)	S1A	C2A	C3A	C10	177.3(17)
C8	C9	C10	C4	-4.2(5)	S1A	C2A	C3A	C11A	-10(4)
C8	C9	C10	C3	168.9(4)	C1A	C9	C10	C4	172.8(15)
C8	C9	C10	C3A	-173.0(9)	C1A	C9	C10	C3A	4.0(18)
C8	C9	C10	C3B	-170.9(15)	C1A	C14	O4A	C15A	-178.0(16)
C8	C9	C1	C14	10.7(15)	C1A	C2A	C3A	C10	3(3)
C8	C9	C1	C2	-170.3(6)	C1A	C2A	C3A	C11A	176(3)
C8	C9	C1A	C14	-14(4)	C2A	C3A	C11A	O1A	148(3)
C8	C9	C1A	C2A	174.9(16)	C2A	C3A	C11A	O2A	-33(4)
C8	C9	C1B	C14	-20(6)	C3A	C11A	O2A	C12A	173.0(17)
C8	C9	C1B	C2B	174(3)	C11A	O2A	C12A	C13A	-170(2)
C8'	C9'	C10'	C3'	175.6(2)	O1A	C11A	O2A	C12A	-8(4)
C8'	C9'	C10'	C4'	-6.3(4)	C17B	S1B	C2B	C1B	32(6)
C9	C10	C3	C2	3.9(7)	C17B	S1B	C2B	C3B	-139(4)

C9	C10	C3	C11	168.9(9)	S1B	C2B	C3B	C10	178(3)
C9	C10	C3A	C2A	-4.3(19)	S1B	C2B	C3B	C11B	-12(7)
C9	C10	C3A	C11A	-177.5(18)	C1B	C9	C10	C4	171(3)
C9	C10	C3B	C2B	-5(3)	C1B	C9	C10	C3B	4(3)
C9	C10	C3B	C11B	-176(3)	C1B	C2B	C3B	C10	5(5)
C9	C1	C2	S1	175.0(7)	C1B	C2B	C3B	C11B	175(5)
C9	C1	C2	C3	-0.1(12)	C2B	C3B	C11B	O1B	146(5)
C9	C1A	C2A	S1A	-174(2)	C2B	C3B	C11B	O2B	-19(6)
C9	C1A	C2A	C3A	-1(4)	C3B	C11B	O2B	C12B	162(3)
C9	C1B	C2B	S1B	-175(4)	C11B	O2B	C12B	C13B	88(3)
C9	C1B	C2B	C3B	-2(6)	O1B	C11B	O2B	C12B	-3(6)
C9'	C1'	C2'	S1'	-171.16(19)	O4	C14	C1	C9	170.9(10)
C9'	C1'	C2'	C3'	4.1(3)	O4	C14	C1	C2	-7.9(15)
C9'	C1'	C14'	O3'	-136.1(3)	O4A	C14	C1A	C9	-153(2)
C9'	C1'	C14'	O4'	43.2(4)	O4A	C14	C1A	C2A	15(4)
C10	C4	C5	C6	1.3(5)					

Table S11. Hydrogen Atom Coordinates (Å×10^4) and Isotropic Displacement Parameters (Å^2×10^3) for **4**.

**Atom**	***x***	***y***	***z***	**U(eq)**
H4	8023.08	5063.34	2993.34	34
H4'	6038.22	4634.74	6153.41	27
H5	7013.43	4874.21	3806.14	31
H5'	6724.72	4253.03	5015.26	28
H7	6285.2	1734.63	3261.53	30
H7'	5199.41	2719.96	3391.35	27
H8	7070.27	1160.07	2264.39	29
H8'	4173.42	2534.93	4175.79	26
H12A	4797.53	6578.89	8403.71	53
H12B	5110.73	5525.55	8980.24	53
H13A	3546.82	6258.2	8873.89	98
H13B	4131.11	6461.42	9696.55	98
H13C	3856.24	5204.41	9444.99	98
H15A	2554.08	663.36	4072.05	55
H15B	2057.1	1609.37	4551.05	55
H16A	1704.88	-121.8	5078.04	110
H16B	2175.92	525.25	5848.55	110
H16C	2591.36	-444.88	5305.89	110
H17A	2447.92	1971	7085.93	55
H17B	2802.24	1556.06	8012.89	55
H17C	3273.53	1350.13	7146.86	55

H17D	11017.09	3009.21	1142.75	128
H17E	10445.84	3453.85	1867.43	128
H17F	10491.15	4105.9	954.61	128
H12C	10009.38	6527.47	2217.19	49
H12D	10074.35	6422.33	3260.83	49
H13D	8722.37	7164.78	2309.45	61
H13E	9309.71	7984.82	2840.92	61
H13F	8802.63	7083.92	3352.21	61
H17G	9033.05	2856.24	-1110.29	44
H17H	8302.11	2681.2	-511.21	44
H17I	8941.07	1704.07	-573.52	44
H12E	10397.14	6220.55	2204.09	56
H12F	10070.18	6433.12	1220.8	56
H13G	10997.22	5128.46	721.72	92
H13H	11342.61	5006.57	1702.31	92
H13I	11405.94	6189.43	1199.63	92
H17J	8898.88	2396.44	-1163.48	59
H17K	8192.6	2291.83	-520.01	59
H17L	8848.89	1333.05	-519.99	59
H12G	9823.59	6280.43	870.56	51
H12H	10506.19	5481.45	540.27	51
H13J	11010.29	6711.82	1591.45	99
H13K	11053.15	5447.98	1968.07	99
H13L	10387.58	6291.47	2269.02	99
H15C	9018.3	-306.26	-516.81	52
H15D	8195.12	-609.88	-123.27	52
H16D	9649.08	-979.84	767.32	92
H16E	9126.56	-1956.02	319.98	92
H16F	8826.26	-1291.92	1153.05	92
H15E	9258.83	-594.45	-80.29	55
H15F	8363.71	-747.57	144.85	55
H16G	9619.91	-1119.62	1375.52	91
H16H	9151.65	-2115.44	880.49	91
H16I	8724.36	-1328.03	1563.8	91

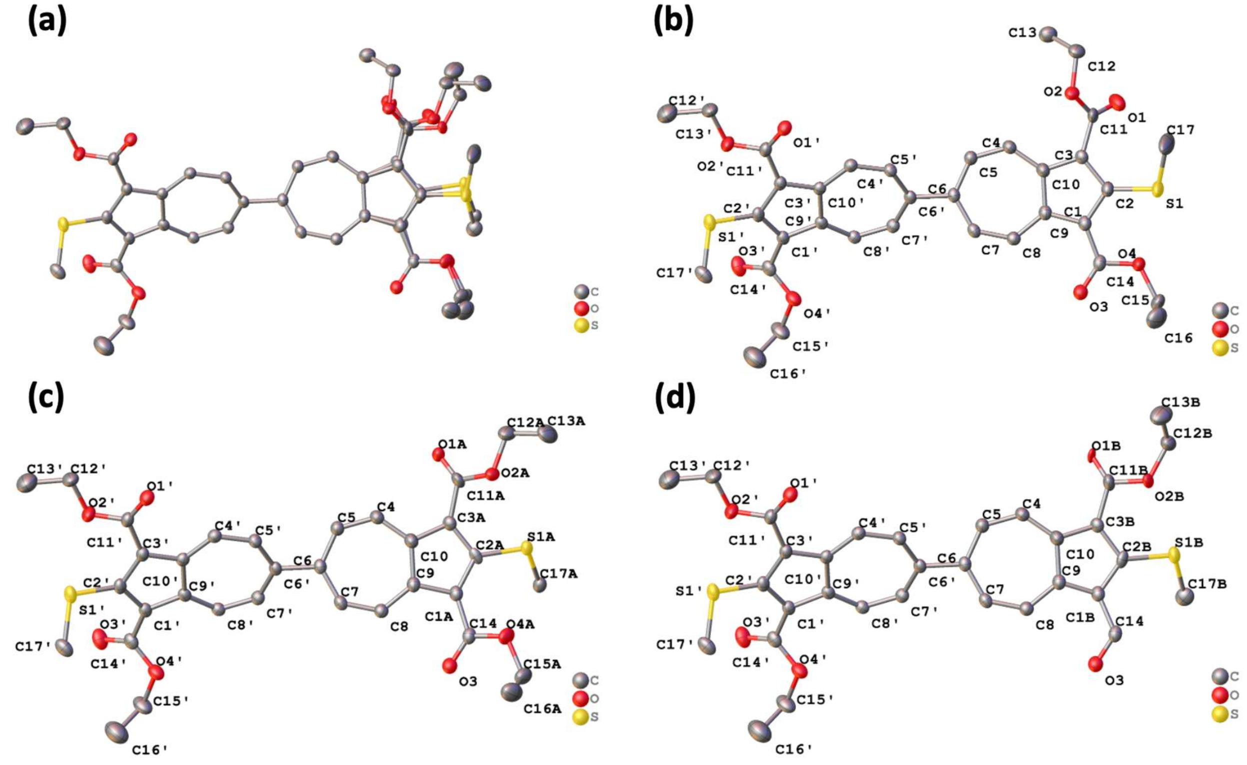

Figure S57. (a) Positional disorder of one of the substituted five-membered rings within **4** modeled/refined by invoking 50%:31%:19% fragment occupancies. (b–d) individual conformers of the three-component disorder.

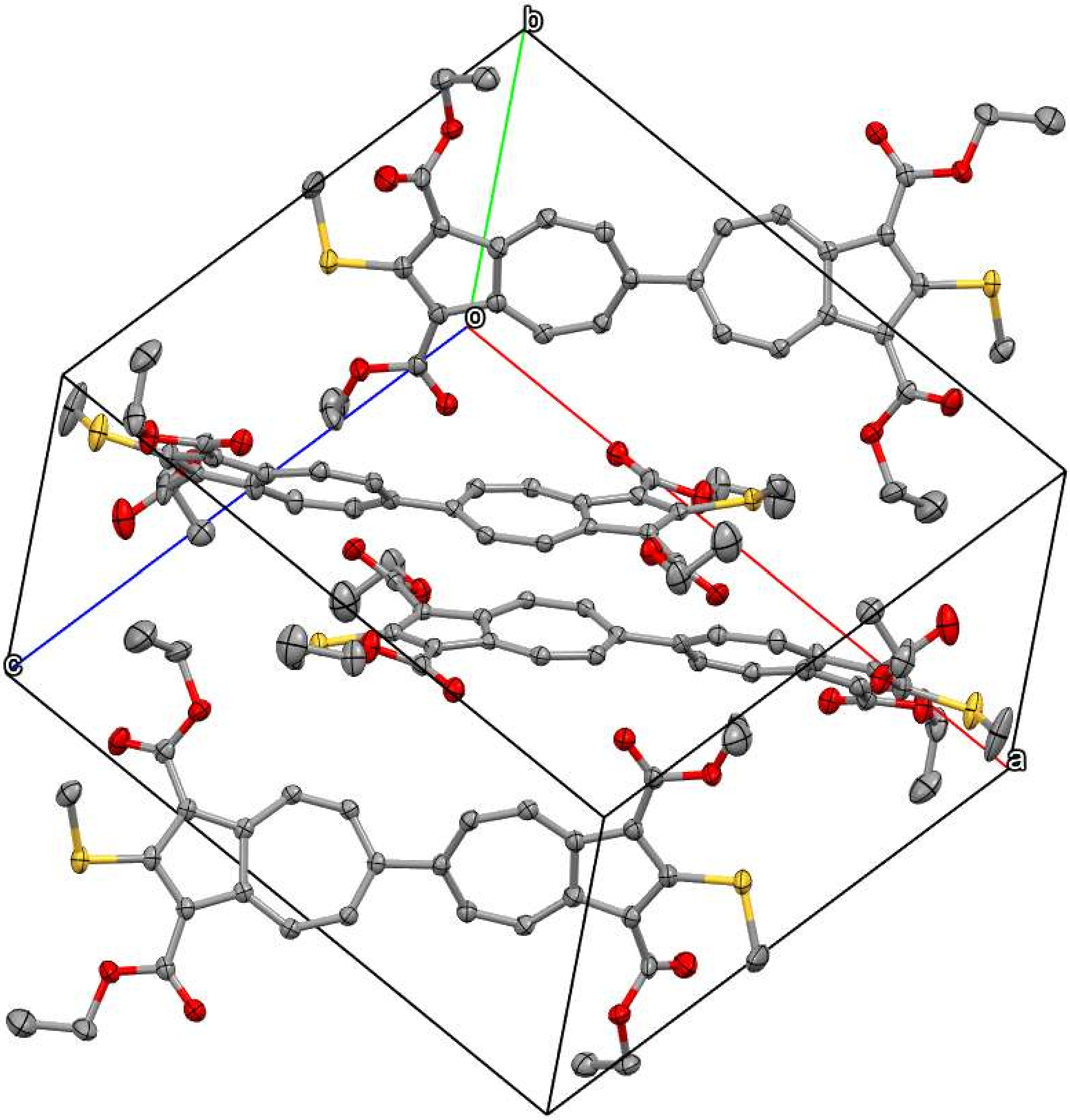

Figure S58. The unit cell of **4**.

G. Computational Work

G1. Calculation Details

All calculations pertaining to compound **3** were performed using version 6.0.0 of the ORCA software package.[11] The geometry optimized structures of **3** were obtained through density-functional theory (DFT) calculation using the B3LYP functional[12] and def2-mTZVP basis with DFT-D3 dispersion energy correction. The resolution of identity chain of spheres approximation (RIJCOSX) operating with the def2/J auxiliary basis was applied for computational efficiency. Disrupting the O⋯H–S hydrogen bonds within **3** was achieved by restricting the H–S–C–C dihedral angles to 90° followed by re-optimization of the entire structure via the above protocol. The LUMO plots for **3** and the HOMO/LUMO plots shown in Figure 1 were generated using UCSF Chimera 1.19 software.

Geometry optimization of **6** was carried out with the B3LYP functional[12] and 6-31+G* basis set using version 5.4 of the Q-Chem software package.[13] Dichloromethane solvent was modeled using the integral equation formalism polarizable continuum model[14] (IEFPCM) with static dielectric constant $\varepsilon = 8.93$.

Table S12. Cartesian coordinates of the atoms in the DFT-optimized structure of **3**.

	X	Y	Z
S	7.2923500296	2.0807807868	17.6789978692
S	0.5091482020	0.2852957550	3.7261802154
O	-0.7447644512	2.8795779532	7.3813738269
O	6.7928684647	4.6175019218	16.6781668783
O	6.0850456396	-1.7949964217	15.3996319297
O	7.0485891595	-0.8957721883	17.2119725282
O	2.7360172967	-1.3672444560	3.7620447550
O	4.2313646935	-1.6742391028	5.4175165726
O	5.9066122615	5.1925644445	14.6898799013
O	-1.1575323149	2.2853865968	5.2605552839
C	6.1247960789	2.8778867109	15.2553175373
C	5.5593153954	2.2954573995	14.0925447057
C	2.6937339377	0.2736718721	7.0643303370
C	5.6179614067	0.8366561223	14.2487896796
C	2.4087895890	-0.1587459313	5.7440683957
C	1.6550074074	1.2350630657	7.4545345970

C	5.0438603501	3.0028799502	13.0018414969
H	5.1180510646	4.0770608452	13.0969884773
C	6.2211870156	0.5690079713	15.5135676145
C	3.2230840995	-1.1291279210	4.9994544718
C	4.2327029050	1.2466232054	11.3708182845
C	6.5314205436	1.8271443838	16.1220020945
C	5.1652389580	-0.0956034230	13.3118844105
H	5.2882656823	-1.1270076439	13.5999788083
C	6.4964762791	-0.7305430918	16.1305726965
C	3.5736178910	1.0855460550	10.0365994778
C	6.2463832994	4.3252110877	15.4777964946
C	4.4649366162	2.5512910136	11.8255868226
H	4.1710834605	3.3408075908	11.1428513471
C	3.7787594639	-0.1503987703	7.8383361066
H	4.4250978758	-0.8595774795	7.3407011859
C	4.1590362469	0.1961288555	9.1260769297
H	5.0714309520	-0.2822436625	9.4644194520
C	4.5690930479	0.0816106628	12.0690806437
H	4.2983529176	-0.8454366938	11.5757801101
C	1.2310961932	0.5062188868	5.3066664820
C	0.7568609805	1.3600952451	6.3534307350
C	1.5878330568	1.8987847977	8.6819957109
H	0.7496061581	2.5673910690	8.7924554742
C	-0.4466165410	2.1891014799	6.2540347862
C	2.4177083576	1.8363389428	9.7948961447
H	2.0921826095	2.4591536492	10.6207763287
C	-1.9293681635	3.7196786156	7.3535020037
H	-2.0063971663	4.1904723357	6.3752497567
H	-1.7257088739	4.4844215006	8.1025975169
C	6.3276984073	-3.1158695505	15.9529293855
H	7.2962359549	-3.1188553125	16.4494043439
H	6.3737208999	-3.7625024090	15.0770351608
C	3.4583044720	-2.3038699654	2.9243328943
H	4.5261315092	-2.1825436749	3.1001496046
H	3.2228960468	-1.9859858969	1.9093120496
C	6.9709530811	6.0167748829	17.0116015236
H	7.2745252020	6.5563961321	16.1156148160
H	7.7936468139	6.0143408353	17.7256059783
C	5.2191598409	-3.5410752034	16.8955061013
H	4.2481485812	-3.5075423598	16.3983356882
H	5.1862725594	-2.8992395558	17.7752792655
H	5.3952746916	-4.5668818814	17.2283946744
C	-3.1812431152	2.9346362830	7.6923284338

H	-4.0362704491	3.6132980915	7.7416724907
H	-3.0832952463	2.4406840788	8.6605745984
H	-3.3883617613	2.1817890839	6.9325410697
C	3.0141149966	-3.7320458934	3.1735161238
H	3.2653031828	-4.0488425125	4.1856126148
H	3.5186967524	-4.4009143049	2.4718065409
H	1.9375554079	-3.8355811551	3.0287429611
C	5.7145646586	6.6115244736	17.6175917130
H	5.9101218100	7.6401695721	17.9306626364
H	5.3982208376	6.0429196232	18.4935433594
H	4.9005936480	6.6253024859	16.8932353232
H	7.4165239910	0.7474129813	17.9230515484
H	-0.4926608452	1.1723671820	3.9759722555

Table S13. Cartesian coordinates of the atoms in the DFT-optimized structure of 3 in which the O…H–S hydrogen bonds were disrupted by restricting the H–S–C–C dihedral angles to 90°.

	X	Y	Z
S	7.61420074	1.31349734	17.6057426
S	-0.0178363	0.04538552	4.02756105
O	-0.7137245	2.89936347	7.34311405
O	6.36553557	4.02788956	16.8340315
O	6.79185388	-2.3200885	15.1967997
O	6.35628194	-1.578705	17.2876436
O	2.01669918	-2.2578066	4.33081937
O	4.00241671	-1.6880272	5.2362742
O	6.47190122	4.56233687	14.6452595
O	-1.8918582	1.30220132	6.26003717
C	6.24697637	2.27219609	15.2747925
C	5.6190724	1.73404783	14.1228622
C	2.40610934	-0.1378745	7.24912683
C	5.64905061	0.2641908	14.2557771
C	2.0298243	-0.5489171	5.94546529
C	1.37826974	0.81043884	7.7219046
C	5.06004414	2.46639029	13.0797226
H	5.17552814	3.53913563	13.1674141
C	6.29907586	-0.0274022	15.4786658
C	2.79823411	-1.5262641	5.14617414
C	4.11515152	0.73264049	11.4970063
C	6.68987027	1.19268361	16.071142
C	5.10026311	-0.6583924	13.3679546

H	5.2171074	-1.6950746	13.6549691
C	6.47894605	-1.3567447	16.10425
C	3.38907803	0.60033965	10.1901646
C	6.38645252	3.72243585	15.5237301
C	4.40421565	2.03093725	11.9331187
H	4.09172718	2.82566629	11.265365
C	3.525309	-0.5737764	7.95226591
H	4.16881243	-1.2504404	7.40464384
C	3.95543677	-0.2523133	9.23523631
H	4.88874436	-0.7206267	9.52620251
C	4.43769147	-0.4574986	12.1640785
H	4.10309978	-1.3665743	11.6775376
C	0.84695739	0.14125382	5.59808621
C	0.42562443	0.93890637	6.68340348
C	1.33131858	1.42633074	8.97046234
H	0.48424059	2.08167777	9.12552746
C	-0.8449502	1.69755864	6.72081934
C	2.20793967	1.34129838	10.0443248
H	1.91774785	1.92607654	10.9097436
C	-1.9103541	3.71835618	7.432368
H	-2.4734003	3.61453012	6.50624224
H	-1.5289422	4.73599951	7.50671001
C	6.97787327	-3.6642744	15.7153679
H	7.49345139	-3.6007884	16.6723452
H	7.63385369	-4.1397667	14.9872198
C	2.67494746	-3.2110947	3.45825042
H	3.63400621	-2.8013011	3.144954
H	2.01453217	-3.2796179	2.594504
C	6.55471924	5.41963959	17.1952694
H	7.24625368	5.87795701	16.4899411
H	7.02085481	5.37747427	18.1790748
C	5.66339258	-4.4093738	15.8475341
H	5.14069581	-4.4606507	14.8903008
H	5.01423614	-3.930139	16.5797405
H	5.85436205	-5.4321501	16.1813494
C	-2.7587304	3.35476712	8.63579397
H	-3.6127962	4.0333084	8.70052441
H	-2.1881438	3.44432141	9.56239331
H	-3.1406219	2.33761956	8.55261417
C	2.84421756	-4.5575309	4.13498708
H	3.52188324	-4.4840866	4.98534979
H	3.26357737	-5.275634	3.42601014
H	1.88393701	-4.9433981	4.48161796

C	5.23497274	6.16526341	17.238581
H	5.4002154	7.18530655	17.5941017
H	4.53368895	5.6779524	17.9182174
H	4.7847486	6.2193895	16.2475323
H	6.53144753	1.36005032	18.4174741
H	-0.821761	-0.9882512	4.37247649

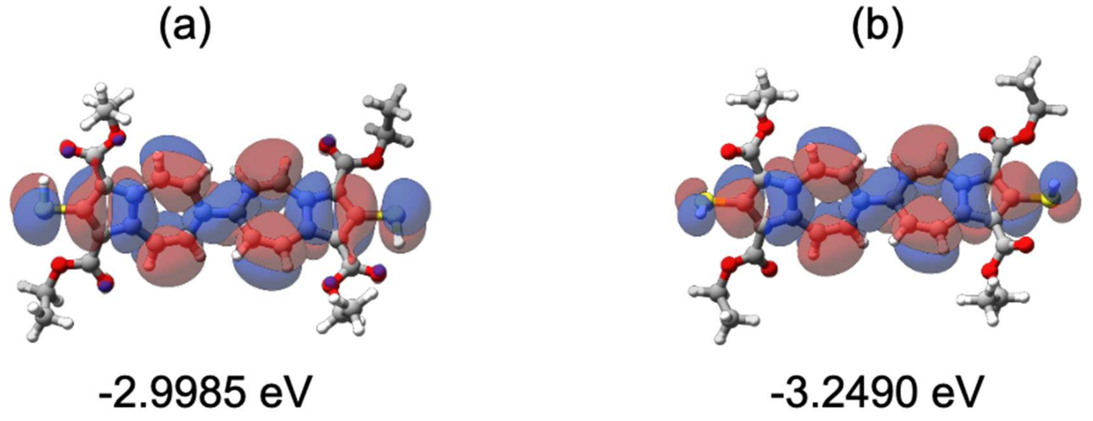

Figure S59. (a) LUMO of the DFT-optimized molecular structure of **3**. (b) LUMO of the DFT-optimized molecular structure of **3** with each S–H bond restricted to be rotated 90° out of the plane of the corresponding azulenyl unit.

Table S14. Cartesian coordinates (in Å) of the atoms in the DFT-optimized (B3LYP/6-31+G*) structure of **6**.

	**X**	**Y**	**Z**
C	-5.3033682122	0.8112931734	0.8485213789
C	-3.9289692738	0.4991162527	0.6614960190
C	-3.8615372649	-0.8383449256	0.0470208285
C	-5.1902342286	-1.3191726276	-0.0777960241
C	-6.0872284990	-0.3051135347	0.3994772839
C	-2.8359359356	1.3361607973	0.9349903808
C	-1.4682217882	1.1301843626	0.7551704457
C	-0.7623180399	0.0104205429	0.2758454565

C	-1.3546741995	-1.1863774000	-0.1726522345
C	-2.6957431228	-1.5470979248	-0.2845988504
C	-5.4948718962	-2.7189483899	-0.4084499823
O	-4.8844925884	-3.3933267995	-1.2360090511
N	-7.4455929081	-0.3882954692	0.4197116792
C	-8.2689803206	0.2850043902	1.4174901166
C	-5.8416481359	2.1532445727	1.1212716257
O	-6.8127483481	2.6462399614	0.5550439916
C	0.7331761042	0.0953466381	0.2452843143
C	1.3089620586	1.2586206138	-0.2995730835
C	2.6453122567	1.6139011681	-0.4836357753
C	3.8254236605	0.9312387804	-0.1501247506
C	3.9064559409	-0.3688563556	0.5386789502
C	5.2797596291	-0.6799123061	0.7056124140
C	6.0589687520	0.4092202553	0.1873393957
C	5.1561936635	1.3965459418	-0.3348755701
C	5.7413042577	-2.0136242876	1.1187024152
O	5.2076134409	-2.7043810602	1.9846584445
C	2.8282457465	-1.1909040013	0.9032367846
C	1.4556795254	-0.9964750874	0.7654432873
C	5.5336300961	2.7802121109	-0.6624748197
O	6.4195401698	3.4144139818	-0.0970260004
N	7.4172138103	0.4930015758	0.1961901808
C	8.2357799824	-0.0860623411	1.2568883096
O	4.7840146332	3.3218623082	-1.6509858809
C	5.0170248097	4.7231290173	-1.9661732077
C	4.0398655568	5.1173236169	-3.0577109522
C	8.1773762587	1.2181673222	-0.8160535615
O	6.7959289629	-2.4554907785	0.3991071072
C	7.3343308582	-3.7604592648	0.7485006783
C	8.4450358362	-4.0798253914	-0.2339536393
O	-6.4907432847	-3.2376575742	0.3429703495
C	-6.8625993865	-4.6192160240	0.0837835456
C	-7.9596567675	-4.9930610969	1.0628641166
O	-5.1400391679	2.8301391749	2.0605175001
C	-5.5294075455	4.2087719725	2.3153287337
C	-4.5919885349	4.7616081546	3.3723195100
C	-8.2096752742	-1.0962467972	-0.6023942416
H	2.7896750869	2.5661605657	-0.9826937442
H	3.1087475011	-2.1163521774	1.3960492928
H	0.6030523212	1.9931285839	-0.6805350841
H	-2.8659552784	-2.5277695182	-0.7176836945
H	-0.6621144639	-1.9450513158	-0.5297989066

H	0.8524000562	-1.8070603261	1.1670164902
H	-3.0900158231	2.2920199566	1.3797843240
H	-0.8517946907	1.9636237919	1.0838115163
H	-6.5708591220	4.2173973415	2.6500094363
H	-5.4638551620	4.7667822806	1.3761150476
H	6.5250250230	-4.4945967803	0.6992864877
H	7.6991309524	-3.7172665251	1.7800198444
H	-5.9752284144	-5.2464252175	0.2097699659
H	-7.1963582222	-4.6990133682	-0.9558566540
H	6.0559765939	4.8351016613	-2.2901049361
H	4.8744511331	5.3124295641	-1.0551450964
H	8.0650827639	-4.1087109160	-1.2611762244
H	8.8649849215	-5.0637583470	0.0048476426
H	9.2510490254	-3.3397549130	-0.1788156570
H	-3.5516666610	4.7409036782	3.0294314157
H	-4.6660177744	4.1891810850	4.3035468460
H	-4.8623572365	5.8021673177	3.5861146555
H	-8.8464243766	-4.3642592693	0.9266256896
H	-7.6142237820	-4.8930539180	2.0977625295
H	-8.2503882067	-6.0369097199	0.8971373024
H	4.1857544490	4.5088995680	-3.9571396865
H	4.2014126044	6.1682580576	-3.3240918393
H	3.0032030121	5.0042152282	-2.7218795615
H	-8.9678473391	-0.4097367833	-1.0021006603
H	-7.5620482766	-1.4020550393	-1.4249620919
H	-8.7157766199	-1.9779901629	-0.1933229563
H	9.0407808142	0.6022293265	-1.0963925938
H	7.5684540405	1.3776958678	-1.7079015313
H	8.5359920006	2.1860515525	-0.4481679193
H	8.8333703032	-0.9299103897	0.8944508570
H	8.9155834182	0.6906211568	1.6313244385
H	7.6100391721	-0.4174262176	2.0863776101
H	-8.7787978426	1.1636384334	1.0057910425
H	-9.0224011861	-0.4292968096	1.7732024733
H	-7.6607310019	0.5914584484	2.2696833112

H. Surface Studies

H1. Self-assembled monolayer films (SAMs) of 15 on Au(111) surfaces. Commercial gold-coated silicon substrates (Platypus Technologies) featuring (111) preferred orientation normal to the substrate[15] were soaked sequentially in distilled chloroform, acetone, and 200-proof ethanol for two hours in each solvent. The bare gold substrates were thoroughly dried under a stream of N_2 gas, and their ellipsometric physical constants *n* and *k* were measured. SAM films of **15** were formed by placing four separate, freshly cleaned 1×1 cm^2 gold substrates into 2 mM solutions of **15** in $CHCl_3$ for *ca.* 24 hrs. In another experiment, monolayer films of **15** were obtained via the SAM displacement route by immersing SAM films of $(OC)_5Cr$[2,2'-diisocyano-1,1',3,3'-terta(ethoxycarbonyl)-6,6'-biazulene] on 1×1 cm^2 Au(111)[7] substrates into a solution of mercaptobiazulene **15** for a period of 24 hrs. Regardless of the method of preparation (i.e., direct chemisorption on Au(111) or displacement of an existing isocyanide-anchored SAM), prior to their analysis, all SAMs were rinsed thoroughly with $CHCl_3$ and dried in a flow of N_2 gas.

H2. Reflection Absorption Infrared (RAIR) spectroscopic measurements. The grazing incidence Reflection Absorption Fourier Transform Infrared spectra were recorded using a Thermo Nicolet Nexus 670 FTIR spectrometer with a VeeMax grazing angle accessory set at an angle of 70°. A background spectrum was collected using a freshly cleaned bare gold substrate before acquiring the spectrum of each sample. Ten thousand scans from 600 to 4000 cm^{-1} at 2 cm^{-1} resolution were collected for each background/sample combination.

H3. Optical ellipsometry. Thicknesses of the SAM films were determined using an Auto EL III ellipsometer (Rudolph Research). All measurements were conducted with a HeNe laser at a wavelength of 632.8 nm and an incident angle of 70° to the surface normal. The optical constants n and k were obtained for each sample individually by measuring these parameters for the corresponding freshly cleaned bare gold substrates prior to the SAM formation. These optical constants were used as input in determining thicknesses of the adsorbed thin layers. A refractive index of 1.45 was assumed[16,17,18] for the organometallic thin films described herein. In the experiments involving the direct route of forming SAMs of **15** via chemisorption of this mercaptoazulene on Au(111), four different SAM-coated substrates were subjected to

ellipsometric thickness measurements. On each sample, five measurements were taken, and the reported film thickness value constitutes an average of 20 ellipsometric measurements across all four SAM samples.

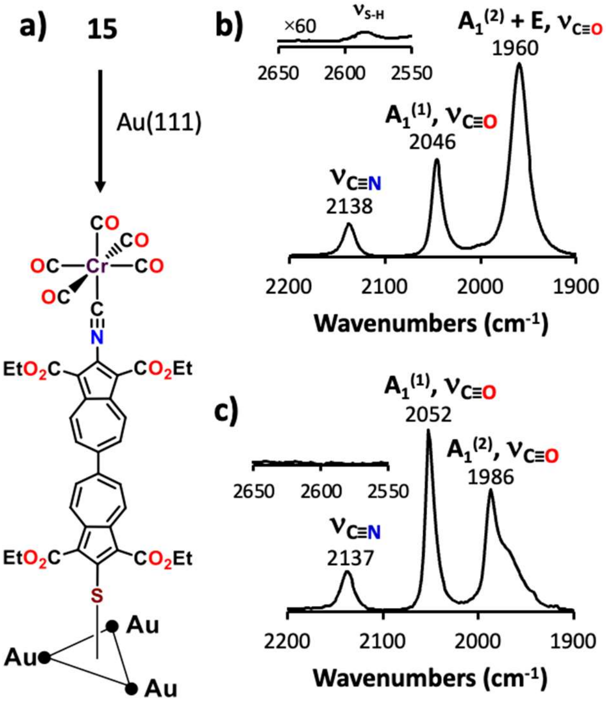

Figure S60. (a) Direct self-assembly of mercaptobiazulene **15** on the Au(111) surface from its solution in $CHCl_3$. (b) FTIR spectrum of **15** in $CHCl_3$. (c) RAIR spectrum of the resulting S-anchored SAM.

References

1. S. R. Kelsey, G. Griaznov, A. D. Spaeth, D. E. Janzen, J. T. Douglas, W. H. Thompson and M. V. Barybin, *Chem. Commun.*, 2024, **60**, 5213–5216.
2. H. Xin, C. Ge, X. Yang, H. Gao, X. Yang and X. Gao, *Chem. Sci.*, 2016, **7**, 6701–6705.
3. J. C. Applegate, M. K. Okeowo, N. R. Erickson, B. M. Neal, C. L. Berrie, N. N. Gerasimchukand and M. V. Barybin, *Chem. Sci.*, 2016, **7**, 1422–1429.
4. N. Mezailles, L. Ricard and F. Gagosz, *Org. Lett.*, 2005, **7**, 4133–4136.
5. T. R. Maher, A. D. Spaeth, B. M. Neal, C. L. Berrie, W. H. Thompson, V. W. Day and M. V. Barybin, *J. Am. Chem. Soc.*, 2010, **132**, 15924–15926.
6. B. M. Neal, A. S. Vorushilov, A. M. DeLaRosa, R. E. Robinson, C. L. Berrie and M. V. Barybin, *Chem. Commun.*, 2011, **47**, 10803–10805.
7. P. T. Connelly, J. C. Applegate, D. A. Maldonado, M. K. Okeowo, W. C. Henke, A. G. Oliver, C. L. Berrie and M. V. Barybin, *Dalton Trans.*, 2023, **52**, 11419–11426.
8. O. V. Dolomanov, L. J. Bourhis, R. J. Gildea, J. A. K. Howard and H. Puschmann, *J. Appl. Crystallogr.*, 2009, **42**, 339–341.
9. L. J. Bourhis, O. V. Dolomanov, R. J. Gildea, J. A. K. Howard and H. Puschmann, *Acta Crystallogr.*, 2015, **71**, 59-75.
10. G. M. Sheldrick, *Acta Crystallogr.*, 2015, **71**, 3–8.
11. F. Neese, *Wires. Comput. Mol. Sci.*, 2025, **15**.
12. A. D. Becke, *J. Chem. Phys.*, 1993, **98**, 5648–5652.
13. E. Epifanovsky, A. T. B. Gilbert, X. T. Feng, J. Lee, Y. Z. Mao, N. Mardirossian, P. Pokhilko, A. F. White, M. P. Coons, A. L. Dempwolff, Z. T. Gan, D. Hait, P. R. Horn, L. D. Jacobson, I. Kaliman, J. Kussmann, A. W. Lange, K. U. Lao, D. S. Levine, J. Liu, S. C. McKenzie, A. F. Morrison, K. D. Nanda, F. Plasser, D. R. Rehn, M. L. Vidal, Z. Q. You, Y. Zhu, B. Alam, B. J. Albrecht, A. Aldossary, E. Alguire, J. H. Andersen, V. Athavale, D. Barton, K. Begam, A. Behn, N. Bellonzi, Y. A. Bernard, E. J. Berquist, H. G. A. Burton, A. Carreras, K. Carter-Fenk, R. Chakraborty, A. D. Chien, K. D. Closser, V. Cofer-Shabica, S. Dasgupta, M. de Wergifosse, J. Deng, M. Diedenhofen, H. Do, S. Ehlert, P. T. Fang, S. Fatehi, Q. G. Feng, T. Friedhoff, J. Gayvert, Q. H. Ge, G. Gidofalvi, M. Goldey, J. Gomes, C. E. González-Espinoza, S. Gulania, A. O. Gunina, M. W. D. Hanson-Heine, P. H. P. Harbach, A. Hauser, M. F. Herbst, M. H. Vera, M. Hodecker, Z. C. Holden, S. Houck, X. K. Huang, K. Hui, B. C. Huynh, M. Ivanov, A. Jasz, H. Ji, H. J. Jiang, B. Kaduk, S. Kähler, K. Khistyaev, J. Kim, G. Kis, P. Klunzinger, Z. Koczor-Benda, J. H. Koh, D. Kosenkov, L. Koulias, T. Kowalczyk, C. M. Krauter, K. Kue, A. Kunitsa, T. Kus, I. Ladjánszki, A. Landau, K. Lawler, D. Lefrancois, S. Lehtola, R. R. Li, Y. P. Li, J. S. Liang, M. Liebenthal, H. H. Lin, Y. S. Lin, F. L. Liu, K. Y. Liu, M. Loipersberger, A. Luenser, A. Manjanath, P. Manohar, E. Mansoor, S. F. Manzer, S. P. Mao, A. Marenich, T. Markovich, S. Mason, S. A. Maurer, P. F. McLaughlin, M. F. S. J. Menger, J. M. Mewes, S. A. Mewes, P. Morgante, J. W. Mullinax, K. J. Oosterbaan, G. Paran, A. C. Paul, S. K. Paul, F. Pavosevic, Z. Pei, S. Prager, E. Proynov, A. Rak, E. Ramos-Cordoba, B. Rana, A. E. Rask, A. Rettig, R. M. Richard, F. Rob, E. Rossomme, T. Scheele, M. Scheurer, M. Schneider, N. Sergueev, S. M. Sharada, W. Skomorowski, D. W. Small, C. J. Stein, Y. C. Su, E. J. Sundstrom, Z. Tao, J. Thirman, G. J. Tornai, T.

Tsuchimochi, N. M. Tubman, S. P. Veccham, O. Vydrov, J. Wenzel, J. Witte, A. Yamada, K. Yao, S. Yeganeh, S. R. Yost, A. Zech, I. Y. Zhang, X. Zhang, Y. Zhang, D. Zuev, A. Aspuru-Guzik, A. T. Bell, N. A. Besley, K. B. Bravaya, B. R. Brooks, D. Casanova, J. D. Chai, S. Coriani, C. J. Cramer, G. Cserey, A. DePrince, R. DiStasio, A. Dreuw, B. D. Dunietz, T. R. Furlani, W. Goddard, S. Hammes-Schiffer, T. Head-Gordon, W. J. Hehre, C. P. Hsu, T. C. Jagau, Y. S. Jung, A. Klamt, J. Kong, D. S. Lambrecht, W. Z. Liang, N. J. Mayhall, C. W. McCurdy, J. B. Neaton, C. Ochsenfeld, J. A. Parkhill, R. Peverati, V. A. Rassolov, Y. H. Shao, L. Slipchenko, T. Stauch, R. P. Steele, J. E. Subotnik, A. J. W. Thom, A. Tkatchenko, D. G. Truhlar, T. Van Voorhis, T. A. Wesolowski, K. B. Whaley, H. Woodcock, P. M. Zimmerman, S. Faraji, P. M. W. Gill, M. Head-Gordon, J. M. Herbert and A. Krylov, *J. Chem. Phys.*, 2021, **155**.

14. E. Cances, B. Mennucci and J. Tomasi, *J. Chem. Phys.*, 1997, **107**, 3032–3041.
15. M. Aguilar, E. Anguiano, J. A. Aznárez and J. L. Sacedón, *Surf. Sci.*, 2001, **482**, 935–939.
16. S. C. Clear and P. F. Nealey, *Langmuir*, 2001, **17**, 720–732.
17. J. D. Legrange, J. L. Markham and C. R. Kurkjian, *Langmuir*, 1993, **9**, 1749–1753.
18. S. R. Wasserman, G. M. Whitesides, I. M. Tidswell, B. M. Ocko, P. S. Pershan and J. D. Axe, *J. Am. Chem. Soc.*, 1989, **111**, 5852–5861.
